# A Comprehensive Annotation of the Channel Catfish (*Ictalurus punctatus*) T Cell Receptor Alpha/Delta, Beta, and Gamma Loci

**DOI:** 10.3389/fimmu.2021.786402

**Published:** 2021-11-25

**Authors:** Jonathan Crider, Sylvie M. A. Quiniou, Kristianna L. Felch, Kurt Showmaker, Eva Bengtén, Melanie Wilson

**Affiliations:** ^1^ Department of Microbiology and Immunology, University of Mississippi Medical Center, Jackson, MS, United States; ^2^ Warmwater Aquaculture Research Unit, United States Department of Agriculture - Agricultural Research Service (USDA-ARS), Stoneville, MS, United States; ^3^ Department of Pharmacology and Toxicology, University of Mississippi Medical Center, Jackson, MS, United States; ^4^ Department of Data Science, John D. Bower School of Population Health, University of Mississippi Medical Center, Jackson, MS, United States

**Keywords:** T cell receptor repertoire; TRB locus, TRAD locus, TRG locus, teleost fish, catfish, IMGT

## Abstract

The complete germline repertoires of the channel catfish, *Ictalurus punctatus*, T cell receptor (TR) loci, TRAD, TRB, and TRG were obtained by analyzing genomic data from PacBio sequencing. The catfish TRB locus spans 214 kb, and contains 112 TRBV genes, a single TRBD gene, 31 TRBJ genes and two TRBC genes. In contrast, the TRAD locus is very large, at 1,285 kb. It consists of four TRDD genes, one TRDJ gene followed by the exons for TRDC, 125 TRAJ genes and the exons encoding the TRAC. Downstream of the TRAC, are 140 TRADV genes, and all of them are in the opposite transcriptional orientation. The catfish TRGC locus spans 151 kb and consists of four diverse V-J-C cassettes. Altogether, this locus contains 15 TRGV genes and 10 TRGJ genes. To place our data into context, we also analyzed the zebrafish TR germline gene repertoires. Overall, our findings demonstrated that catfish possesses a more restricted repertoire compared to the zebrafish. For example, the 140 TRADV genes in catfish form eight subgroups based on members sharing 75% nucleotide identity. However, the 149 TRAD genes in zebrafish form 53 subgroups. This difference in subgroup numbers between catfish and zebrafish is best explained by expansions of catfish TRADV subgroups, which likely occurred through multiple, relatively recent gene duplications. Similarly, 112 catfish TRBV genes form 30 subgroups, while the 51 zebrafish TRBV genes are placed into 36 subgroups. Notably, several catfish and zebrafish TRB subgroups share ancestor nodes. In addition, the complete catfish TR gene annotation was used to compile a TR gene segment database, which was applied in clonotype analysis of an available gynogenetic channel catfish transcriptome. Combined, the TR annotation and clonotype analysis suggested that the expressed TRA, TRB, and TRD repertoires were generated by different mechanisms. The diversity of the TRB repertoire depends on the number of TRBV subgroups and TRBJ genes, while TRA diversity relies on the many different TRAJ genes, which appear to be only minimally trimmed. In contrast, TRD diversity relies on nucleotide additions and the utilization of up to four TRDD segments.

## Introduction

T cells receptors (TR) are the primary antigen receptors of T cell lymphocytes, and TR are expressed either as an alpha-beta (αβ) or gamma-delta (γδ) heterodimer on T cells in jawed vertebrates. Conventional T helper cells (TH) and cytotoxic T lymphocytes (CTL) express αβ TR and recognize linear peptide antigens presented in the context of MHC class I or MHC class II proteins. In contrast, γδ T cells can recognize a broader range of antigens presented on butyrophilins, MHC-like molecules, MHC-related protein (MR-1), or by directly binding antigen, i.e. Ig-like binding (1, 2). In most vertebrates αβ T cells constitute the major T cell population in circulation, however, cows (*Bos taurus*), pigs (*Sus domesticu*s), sheep (*Ovis aries*) and chickens (*Gallus gallus domesticus*) are considered “γδ high” and approximately 50% of their T cells express TRG and TRD (3–8). Interestingly, Haase et al. (9), did not find any evidence of TRG genes in Pipefish (*Syngnathus typhle*), and in 2021 Mirete-Bachiller et al. (10), also did not find any evidence of TRG or TRD genes in blunt-snouted clingfish (*Gouania willdenowi*). TR cDNA sequences have been identified in multiple teleost species, beginning with the rainbow trout (*Oncorhynchus mykiss*; 11, 12), Atlantic salmon (*Salmo salar*; 13), channel catfish (*Ictalurus punctatus*; 14), Atlantic cod (*Gadus morhua;* 15), green spotted pufferfish (*Tetraodon nigroviridis*; 16) and later in sea bass (*Dicentrarchus labrax*; 17) and loach (*Misgurnus anguillicaudatus*; 18). Similarly, the genomic organization of a complete TR locus, has only been determined in a few teleosts. For example, the *TRG* and *TRAD* loci have been sequenced in the Atlantic salmon (*Salmo salar*; 19, 20), and the *TRB* and *TRAD* loci have been annotated in the zebrafish (*Danio rerio*) (21, 22). To date, the full germline TR repertoire, has only been determined in the cyprinid minifish, “*Paedocypris* sp. *Singkep*” (23).

The channel catfish (*Ictalurus punctatus*) is an economically important aquaculture species and valuable model for comparative immunology. One of the benefits of the catfish model is the availability of an *in vitro* cell culture system, which has facilitated the development of long-term leukocyte cell lines from catfish tissues and peripheral blood (24–27). Such clonal lines and mixed leukocyte cultures (MLCs) have yielded important insights into catfish T cell biology, TR and gene organization (14, 28–32). For example, early on our laboratory developed several clonal T cell lines, including alloantigen-specific cytotoxic T lymphocytes (CTLs) and more recently, channel catfish virus (CCV)-specific CTLs were also cloned (33, 34). The expressed TR repertoire in these cell lines was determined by 5’RACE and cDNA sequencing, and a total of six TRAV, eight TRBV, two TRDV and three TRGV subgroups (families) were identified (14, 33–35). In addition, Zhou et al. (36), sequenced the catfish TRB D-J-C region using overlapping λ-phage clones from a genomic library made from an outbred catfish and demonstrated that the catfish TRB locus encoded two TRBC genes (TRBC1 and TRBC2), 29 TRBJ genes, and a single TRBD gene. However, a full comprehensive annotation of the channel catfish TR loci was not feasible at the time. This lack of a database has hampered studies of catfish T cell responses to infectious diseases (37). Like many of our colleagues, we recognize that defining the full potential repertoire, i.e. the number of germline TR gene segments and their diversity, is essential for studying the expressed repertoire after vaccination or pathogen challenge. In addition, this catfish germline TR repertoire will form a basis for comparisons with other species and provide insight into TR gene evolution.

Here in this report, we present the complete TR germline repertoire of two immune model species, the channel catfish, and the zebrafish. Our goals for this annotation are to 1) report the complete and comprehensive annotation of the channel catfish TRB, TRAD, and TRG loci and their germline gene organizations, as obtained through a combination of bacterial artificial chromosome (BAC) and whole-genome sequencing, 2) compare the catfish TR germline with the zebrafish TR germline repertoire, and 3) place the earlier catfish TR studies into context. All together, we show that there are 267 TRV genes, 167 TRJ genes, and five TRD genes in the catfish genome. Combined these V genes represent 30 TRBV subgroups, six TRAV subgroups, two TRDV subgroups and three TRGV subgroups. In comparison, the zebrafish genome contains 207 TRV genes, 151 TRJ genes and seven TRD gene segments. These 207 TRV genes constitute 36 TRBV subgroups, 53 TRADV subgroups and four TRGV subgroups.

## Materials and Methods

### Channel Catfish TR Gene Identification

Amino acid sequences of previously identified catfish TRV, TRD and TRJ sequences were formatted into a single multi-fasta file. These multi-sequence files were then used as tBLASTn queries to identify gene segments in all six-frames of TRB, TRAD, and TRG loci-containing contigs from a third generation assembly of the CCBL1 channel catfish genome (38, 39, and unpublished). Output hits from the tBLASTn were sorted according to their positions on the contig, examined, and annotated using SnapGene (Insightful Science). The TR genes identified in the initial search were then recompiled into the database and used as queries in a second multi-sequence tBLASTn search. The sorting/cataloging process was then repeated. Finally, the nucleotide sequences from all previously known and newly identified catfish TR genes were used as BLASTn queries. These final searches did not yield any additional catfish TR gene segments. The TR heptamer and nonamer recombination signal sequences, and splice sites were identified manually. This same approach was also used to identify the zebrafish TRB and TRG genes. Sequences that were lacking proper splice sites and/or were encoding stop codon(s) and/or frameshift(s) were classified as pseudogenes. Partial V sequences that were lacking the sequences encoding the leader and/or the 3’ end were classified as remnants. The annotated catfish TRB, TRAD, and TRG loci have been submitted to Genbank under the accession numbers: OL314503, OL314504, and OL314505. The TR gene sequences are listed in **Supplementary Tables 1**-**3**.

### Phylogenetic Analysis and V Subgroup Determination

To determine the catfish TRV gene subgroups, multiple sequence alignments were generated using Molecular Evolutionary Genetics Analysis (MEGA) version X software (40). The TRADV genes were aligned using the CLUSTALW algorithm (41). The TRBV sequences, however, were too divergent to be aligned using CLUSTALW, and the MUSCLE algorithm was used instead (42). Prior to alignment, the sequences were trimmed to encompass the codons for amino acids 1-106 according to the IMGT unique numbering system. The trimmed sequences include 22 codons before the codon for the first cysteine that forms the disulfide bond (CYS23) and extends until six nucleotides past the second cysteine (CYS104). Percent identities were determined by pairwise alignments based on the length of the largest sequence (BLOSUM62 matrix) using the Sequence Identity and Similarity (SIAS)^1^ website (43, 44). Genes which shared 75% nucleotide identity or greater within the matrixes were placed into subgroups and assigned names according to ImMunoGeneTics (IMGT) guidelines (45, 46). For subgroups that contained more than one member, the TRV genes were named with their subgroup number followed by their order in the locus from 5’ to 3’. Phylogenetic trees were generated in MEGA version X (40) by the neighbor-joining method (47) with 10,000 bootstrap replicates (48). Evolutionary distances were computed using the p-distance method (49), and all ambiguous positions removed for each sequence pair.

### Clonotype Analysis

Sequencing reads from an RNA-seq dataset (SRX accession#: SRX113171) were downloaded from the NCBI Sequencing Read Archive (SRA). The largest run (26.7G bases) from this study (SRR accession#: SRR392744), which pooled RNA from 19 tissues isolated from a gynogenetic catfish (50), was used to identify distinct TRB, TRA, TRD, and TRG clonotypes. Briefly, FASTQ files were extracted as forward and reverse reads using fastq-dump (v2.10.8) of the SRA Toolkit software (51). The resulting reads were trimmed for quality, and sequencing adapters were removed by Trimmomatic (v0.39) (52). Overlapping paired reads were merged with FLASh (v1.2.11) allowing for a maximum overlap (-M) of 90 bp, and clonotype analysis was performed using IMSEQ (v1.1.0) (53, 54). Default IMSEQ parameters were used, except for a set minimum read length of 100 bp (-mrl) and merging of ambiguous segments (-ma). The scripts and catfish V/J gene databases, which were formatted for use with IMSEQ, are published in GitHub (55). In addition, RNA-seq reads from two unrelated catfish were also included and analyzed as above. These datasets were obtained from head kidney RNA and contained 28.9 million and 37.9 million, 2x150bp HiSeq3000, Illumina reads, respectively. The matrices for clonotype chord diagrams were constructed by plotting the number of associations between TRV gene subgroups and specific TRJ gene segments. If the TRV gene subgroup or TRJ gene of a clonotype was ambiguous, the clonotype was excluded from the analysis. Matrixes were submitted to Circos^2^ (56), and the generated figures were labeled for clarity using Adobe Illustrator 25.2.3.

### Fish

All experiments involving live catfish were approved by the Institutional Animal Care and Use Committee (IACUC) and performed at the USDA-ARS-WARU, Stoneville, MS according to relevant institutional and national guidelines and regulations.

## Results

### The Catfish TRB Locus

**Figure 1:**
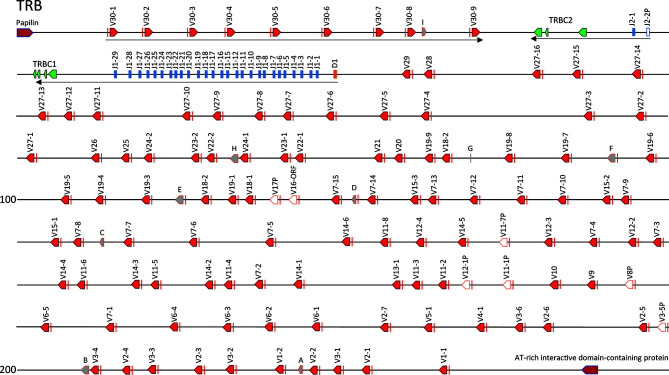
The channel catfish TRB locus. Schematic representation of the channel catfish TRB locus. Red arrows mark the location and direction of transcription for each of the TRBV genes and green arrows mark the location of the TRBC1 and TRBC2 genes. The TRBJ genes are in blue and the TRBD gene is colored orange. Pseudogenes are in white and labeled, P. The five TRB remnants are grey and labeled by letters A – E. The two genes that flank the locus, papilin and AT-rich interactive domain-containing protein (ARID-1) are labeled.

The catfish TRB locus is ~214 kb in length and is flanked by genes encoding papilin and the AT-rich interactive domain-containing protein. The TRBC genes, the TRBJ genes, the TRBD gene, and the majority of the TRBV genes are encoded on the negative strand of the DNA (**Figure 1**). Altogether there are 112 V genes, and nine V remnants present in the locus. Nine of the TRBV genes, and one V remnant, which consists of a leader exon and a V segment that is truncated before the codon for cysteine 104, are present downstream of TRBC2, in the opposite transcriptional orientation. Directly 5’ of TRBC2 are the two TRBJ genes (J2-1 and J2-2P), the TRBC1 exons, 27 functional TRBJ genes and TRBD1. This region was previously sequenced from a λ-phage genomic library made with erythrocyte DNA from outbred catfish, #32 (36). The 27 TRBJ genes 5’ of TRBC1 in the CCBL1 BAC clones match the TRBJ genes described by Zhou et al. (36), with two exceptions. First, the alleles of TRBJ16 and TRBJ17 each contain two nucleotide differences, which translates to one amino acid change in each of these TRBJs. Second, the tandem duplication of TRBJ13 and TRBJ14 identified from fish 21 is absent in the genome of the gynogenetic fish used for the CCBL1 BAC library.

The TRBV gene most proximal to the TRBD and TRBJ genes is located 2,529 bp upstream of TRBD1 and is preceded by 102 additional TRBV genes (**Figure 1**), which are divided into 29 TRBV subgroups based on the ImMunoGeneTics criterion that sequences that share 75% nucleotide identity form a subgroup. The largest subgroups are TRBV27 with 16 members, and TRBV7 with 15 members. The smallest TRBV subgroups contain from 1-3 genes, and there are 14 single member TRBV subgroups. Six TRBV genes are classified as pseudogenes due to their lack of an open reading frame, and one TRBV gene contains a frame shift in the 3’ end, however it remains an open reading frame that encodes a CYS104. This gene is designated as TRBV16-ORF (**Supplementary Figure S1**). Here it is important to note that the two single member subgroups TRBV8P and TRBV17P, which consist of pseudogenes in the channel catfish CCBL1, have homologs that are functional in the red-tailed catfish (*Hemibagrus wyckioides*; accession KAG7328538) and walking catfish (*Clarias magur;* accession KAF5887601), respectively.

**Figure 2:**
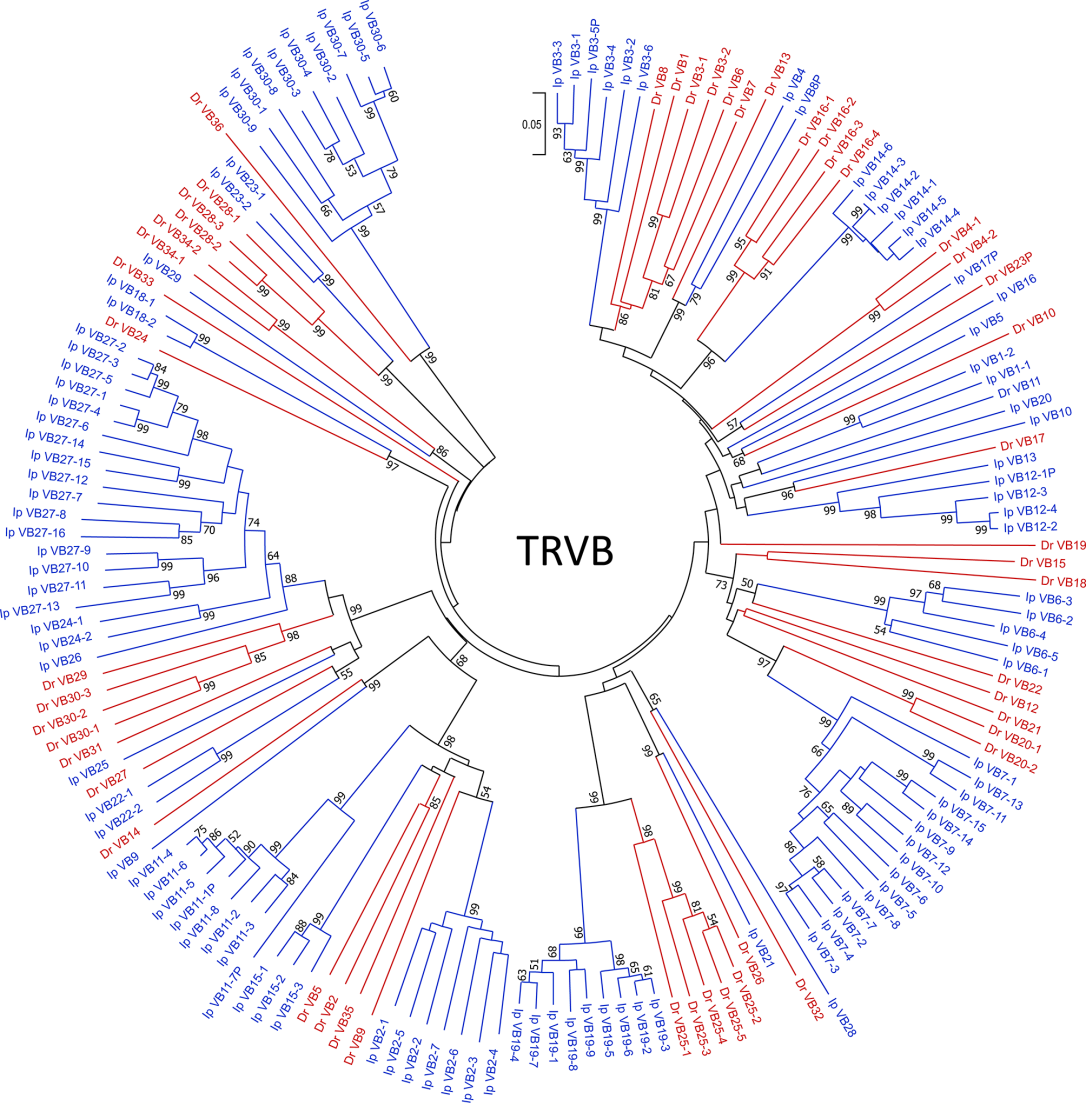
TRBV phylogenetic analysis. TRBV phylogenetic tree topology demonstrates that 10 catfish and zebrafish TRBV subgroups are closely related. The evolutionary history was inferred using the Neighbor-Joining method (47). The percentage of replicate trees in which the associated taxa clustered together in the bootstrap test (10,000 replicates) are shown next to the branches (48). The tree is drawn to scale, with branch lengths in the same units as those of the evolutionary distances used to infer the phylogenetic tree. The evolutionary distances were computed using the p-distance method (49) and are in the units of the number of base differences per site. The analysis involved 163 nucleotide sequences. Prior to analysis, sequences were trimmed to include 22 codons before the codon for the first cysteine (CYS23) to 6 nucleotides past the last cysteine (CYS104). All ambiguous positions were removed for each sequence pair (pairwise deletion option). There were a total of 382 positions in the final dataset, and evolutionary analyses were conducted in MEGA X (40). Zebrafish sequences are colored in red, and catfish are indicated in blue. Bootstrap values below a cutoff of 50 have been omitted for clarity.

**Figure 3:**
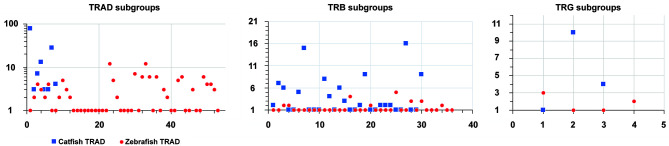
Comparison of the number of genes within each subgroup in the catfish and zebrafish TRAD, TRAB and TRG loci. The diagram depicts the number of subgroups and the number of genes within each subgroup. The subgroup numbers are indicated along the x-axis and the number of genes on the y-axis. Note that the y-axis for the TRAD locus is on a logarithmic scale.

To place the catfish TRB data in context, we annotated the TRB locus from the *Danio rerio* strain Tübingen on chromosome 17, GRCz11 Primary Assembly (accession number NC_007128.7), see **Supplementary Table 4** and built upon Meeker et al., 2010, who first annotated the zebrafish TRB locus (Zv8). In total, 51 zebrafish TRBV gene segments were identified, and these genes could be placed into 36 subgroups. Twenty-three TRBV genes form eight subgroups, and subgroup 25 is the largest with five members. The next largest subgroups are subgroup 16 with four members, and subgroups 28 and 30 with three members each. Four subgroups 3, 4, 20, and 34, each consist of two members, and the remaining 28 TRBV segments form single-member subgroups. The catfish and zebrafish TRBV genes were compared using pairwise alignments, and as expected, their subgroups were not shared. However, as shown in the phylogenetic tree (**Figure 2**) the catfish and the zebrafish TRBV subgroups are closely related, and form 10 distinct clans supported by high boot strap values, ≥ 96. For example, members of zebrafish subgroup 25 share 62.8-71.2% nucleotide identity with TRBV genes in catfish subgroup 19. Similarly, members of the catfish subgroup 7 share 60.8-67.7% nucleotide identity with TRBV genes in zebrafish subgroup 20, and the single-member subgroups catfish TRBV21 and zebrafish TRBV26, share 67.7% nucleotide identity. Therefore, even though the catfish TRB locus contains twice as many TRBV gene segments, they are less diverse and constitute fewer subgroups as compared to the TRBV gene segments encoded within the zebrafish TRB locus (**Figure 3**).

Both the catfish and zebrafish TRB loci encode for 31 TRBJ genes and a single TRBD gene and their TRBD genes are identical (GGGACAGGGGGC). Interestingly, while our phylogenic comparisons of catfish and zebrafish TRBJs did group certain catfish TRBJs with certain zebrafish TRBJs, bootstrap confidence values were not significant. The one exception was the grouping of the catfish TRBJ segments associated with TRBC2: Ip TRBJ2-1 and Ip TRBJ2-2P with zebrafish TRBJ2-1 which had a bootstrap value of 77 (**Supplementary Figure S2**).

### The Catfish TRAD Locus

**Figure 4:**
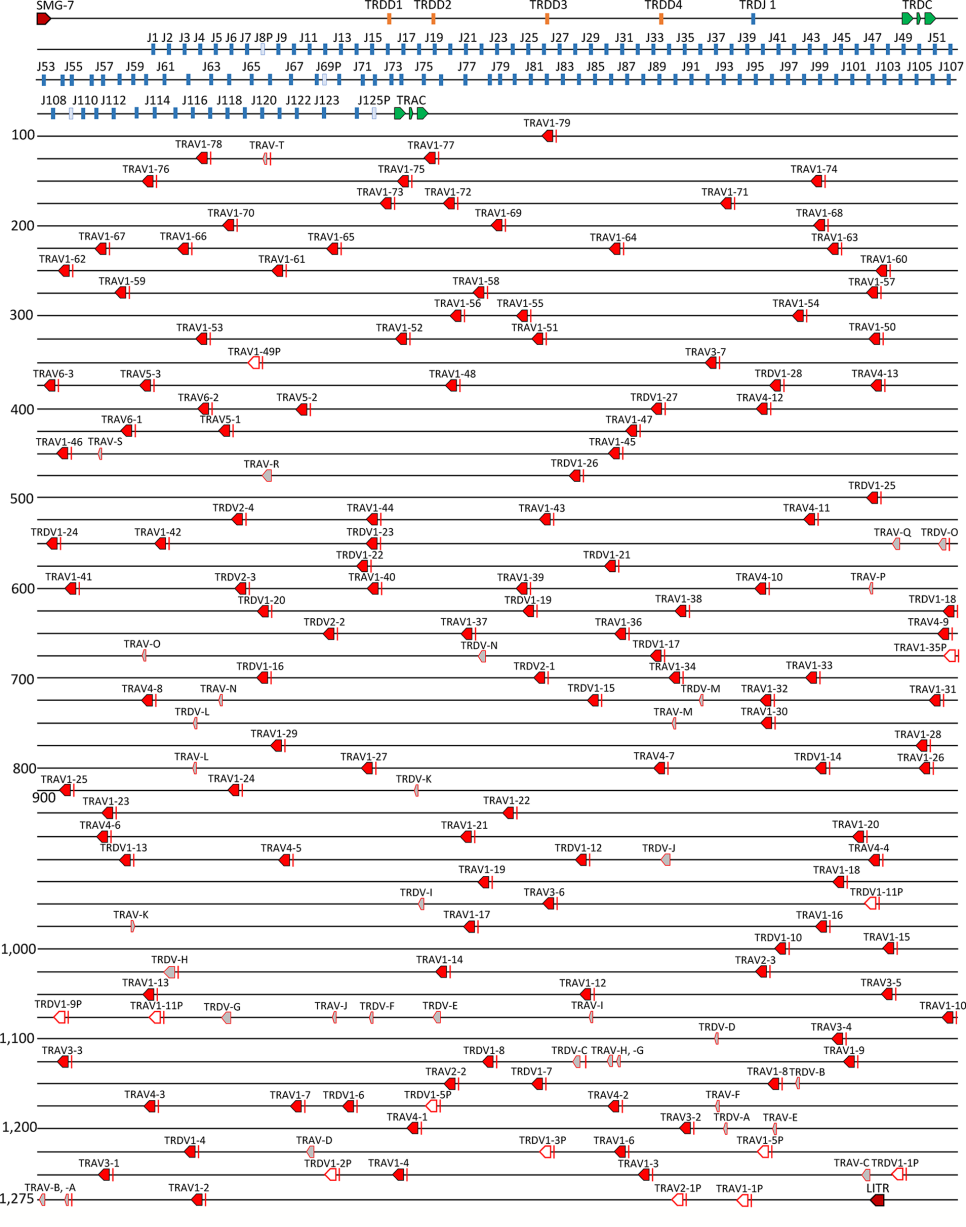
The channel catfish TRAD locus. Schematic representation of the channel catfish TRAD locus. Red arrows mark the location and direction of transcription for each of the TRAV genes and the TRDV genes. Green arrows mark the location of the TRDC and TRAC genes. The TRAJ genes are colored blue and the TRD D genes are in orange. Pseudo J genes are in pale blue, V gene remnants are in grey, and labeled with letters A-T. The two genes that flank the locus, nonsense mediated mRNA decay factor (SMG-7) and leukocyte immune type receptor (LITR) are labeled.

The catfish TRAD locus is ~1,285 kb in length and the 5’ flanking gene of the locus is *SMG-7*, which encodes nonsense mediated mRNA decay factor. The four TRDD genes and the single TRDJ gene span a region of 10,186 bp 3’ of the TRDC. Directly 3,920 bp 3’ of TRDC are 125 TRAJ genes; four of these are pseudogenes (J8P, J69P, J109P, and J125P). The TRAC gene is located 1,380 bp 3’ of J125P, and 27.7 kb downstream of TRAC is the most proximal TRAV gene, TRAV1-79 (**Figure 4**). There are a total of 140 TRADV genes distributed over 1.181 kb in this locus, and all are in the opposite transcriptional orientation to the TRDC and TRAC genes. The TRADV genes form eight subgroups, six of which are designated as TRAV subgroups and two that are designated as TRDV subgroups, based on their phylogenetics and preferential expression. TRAV genes and TRDV genes can also be distinguished by the length of their leader exons. TRAV genes have leader exons of 40-43 nucleotides, while the TRDV leader exons are 64 or 67 nucleotides long. Six of the 140 V genes are TRAV pseudogenes and six are TRDV pseudogenes. There are also 20 TRAV and 15 TRDV remnants scattered throughout the locus. These remnants consist of either the 5’ end or the 3’ end of a V gene. The largest TRAV subgroup (subgroup 1) consists of 79 members, and TRAV subgroups 4 and 3 contain 13 and seven members, respectively. The three remaining TRAV subgroups 2, 5 and 6, consist of three members each. The TRDV subgroup 1 consists of 28 members and subgroup 2 is much smaller with only four members (**Supplementary Figure S3**).

In 2016, Seelye et al. (22), annotated the zebrafish TRAD locus and determined that it encoded 149 TRADV genes, and 111 TRAJ genes, and their findings provided us with the opportunity to compare their annotated TRADV gene data with that of the catfish TRADV genes. As shown in **Supplementary Figure S4**, the catfish and zebrafish TRVAD gene segments segregate independently and do not branch together, however there are a few exceptions. For example, catfish TRAV subgroup 6 branches with zebrafish TRADV17.0 with a bootstrap level of 99, and these sequences share 68% nucleotide identity. Also, members of the zebrafish TRADV subgroup 2 form a branch with catfish TRAV subgroups 2, 3, and 4, with a bootstrap value of 84, and nucleotide identities range from 59-72%. Overall, the TRAV and TRDV genes in the catfish are dominated by a few subgroups, and are less diverse as compared to the zebrafish TRADV genes, e.g. the zebrafish TRADV genes can be placed into 53 subgroups based on sharing 75% nucleotide identity. As such, it is important to note that in the catfish TRAD locus there are three regions of duplications. The first is a 220 kb region that encodes 28 TRAV genes (TRAV1-50 to TRAV1-77) and consist of several repeats ranging in size from 5 to 12kb. These repeats originated from a series of duplication events, and each duplication contains one to three TRAV genes (**Supplementary Figure S5**). The second region is the result of two different tandem duplications. The first tandem duplication resulted in three copies of TRAV5 and TRAV6. The second tandem duplication involved both TRDV1 and TRAV4 and resulted in two tandem gene segments, TRDV1-27-TRAV4-12, and TRDV1-28-TRAV4-13. In contrast, the third region consists of four copies of a TRDV2-TRAV1-TRAV1-TRAV4 array interspersed among copies of the TRDV1 and TRAV1 subgroups. These duplications are supported by phylogenetic analyses (**Supplementary Figure S4**).

The 125 catfish TRAJ genes are distributed over 55,465 bp, and they are quite diverse. The catfish TRAJ genes encode from 17 to 22 amino acids, and based on sharing 75% nucleotide identity, 71 of these genes can be placed into 21 subgroups. The three largest subgroups consist of 12, seven and six TRAJ genes (25 genes), and the next largest subgroups consist of two groups of five TRAJ genes and four groups of three TRAJ genes (22 genes). In addition, there are 12 groups of pairs, and 54 single TRAJ genes scattered throughout the TRAJ region (**Supplementary Figure S6**). Twenty of the TRAJ genes do not encode the canonical FGXG motif, and 10 variations were observed. The most common variants encoded FAXG and FGXA. The FAXG variant is encoded by eight of the catfish TRAJ genes and the FGXA variant is encoded by five of the TRAJ genes. The codons for the FAXG motif are also present in five out of the 111 zebrafish TRAJ genes. Similarly, the single functional TRDJ in Atlantic salmon encodes the FGKA motif (20).

### The Catfish TRG Locus

**Figure 5:**
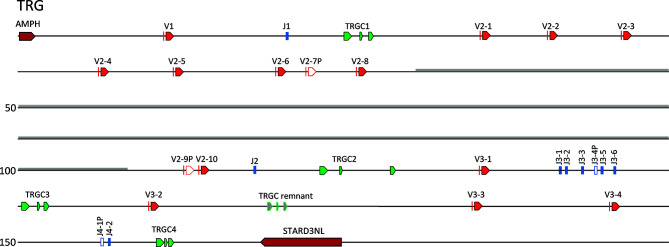
The channel catfish TRG locus. Schematic representation of the channel catfish TRG locus. Red arrows mark the location and direction of transcription for each of the TRGV genes and green arrows mark the location of the TRGC1, TRGC2, TRGC3 and TRGC4 genes. The TRGJ genes are in blue and pseudogenes are in white. The region of repeats is shaded in gray. The two genes that flank the locus, Amphiphysin (*AMPH*) and STARD3 N-terminal like (*STARD3NL*) are labeled.

The catfish TRG locus is ~151 kb in length and is flanked by the genes amphiphysin (*AMPH*) and STARD3 N-terminal like protein (*STARD3NL*). Previously, through primer walking on BACs, we predicted that the catfish TRG locus consisted of three cassettes: (Vγ-Jγ-Cγ2) – (Vγ-Vγ-Vγ-Jγ-Cγ3) – (Vγ-Jγ-Jγ-Jγ-Jγ-Jγ-Cγ1) (35). Here, with this annotation we confirmed the presence of these three cassettes and renamed them according to the recommended IMGT nomenclature. The first cassette consists of TRGV1, TRGJ1 and TRGC1 and the second cassette consists of eight functional TRGV2 genes, two TRGV pseudogenes (V2-7P and V2-9P), TRGJ2 and TRGC2 (**Supplementary Figure S7**). This cassette is interrupted by a 2 kb sequence that is repeated 30 times between TRGV2-8 and the TRGV2-9P. The repeat is flanked by two *NheI* restriction sites and begins 2,041 bp 3’ of the TRGV1-8 nonamer (**Supplementary Figure S8**). A 75 bp sequence present within this 2 kb sequence is also found repeated in other fish genomes, e.g. Atlantic cod, leopard coral grouper, *Plectropomus leopardus*; and zebrafish. The third cassette consists of TRGV3-1, five functional TRGJ genes, one TRGJ pseudogene (TRGJ3-4P) and the TRGC3 gene. In addition, we identified a fourth cassette located 3.7 kb 3’ of TRGC3. This TRGC4 cassette consists of three TRGV genes (V3-2, V3-3, and V3-4), a functional TRGJ, a TRGJ pseudogene, and a TRGC gene (TRGC4; **Figure 5**). In between TRGV3-2 and TRGV3-3 is a TRGC remnant that consist of three exons that encode a partial TRC immunoglobulin domain, a connecting peptide, and a transmembrane region. Because the two TRGJ genes J4-1P and J4-2 share the highest nucleotide identities with J3-1 and J3-2 at 68.5 and 75%, respectively, we aligned the sequence from the TRGV3-1 to TRGJ3-2 with the sequence from TRGV3-4 to TRGJ4-2. The two TRGV genes are 92% identical at the nucleotide level, however their intervening V-J introns could not be easily aligned. Even so, since the exons encoding the immunoglobulin domains of TRGC3 and TRGC4 are 81% identical at the nucleotide level, it seems likely that the fourth cassette is a duplication of the third cassette. While TRGC4 appears functional, we have not found any TRGC4 transcripts in the catfish cDNA databases.

**Figure 6:**
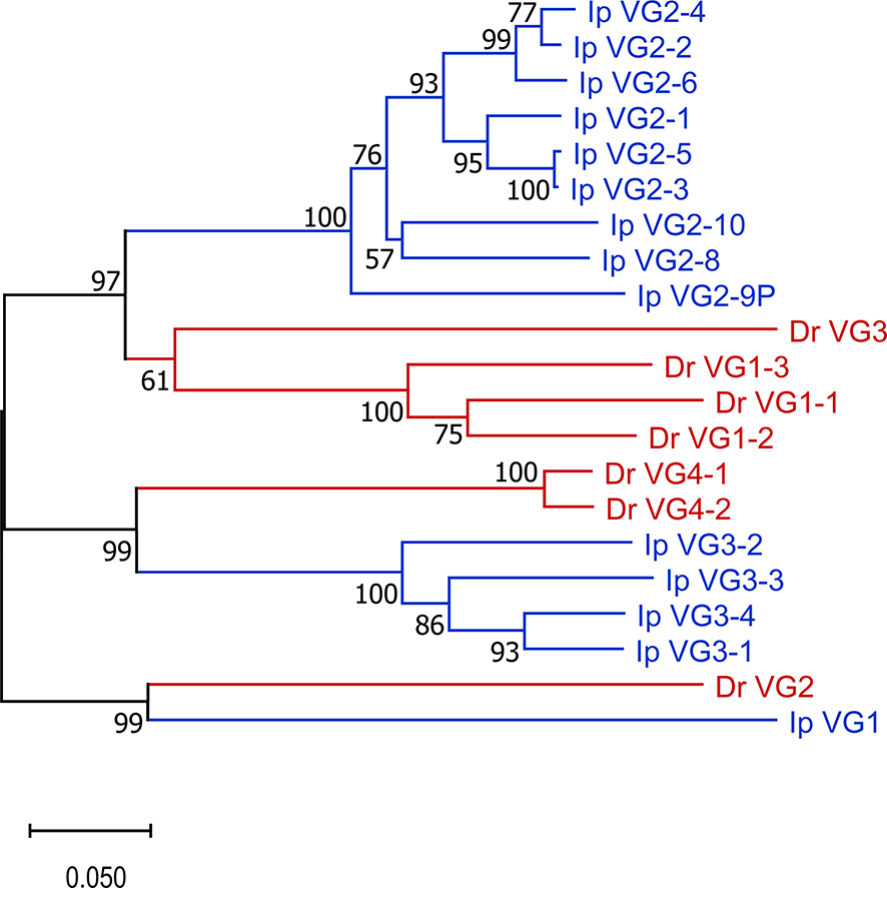
TRGV phylogenic analysis. Catfish TRGV gene subgroups are phylogenetically more closely related to zebrafish TRGV subgroups than to each other. The evolutionary history was inferred using the Neighbor-Joining method (47). The percentage of replicate trees in which the associated taxa clustered together in the bootstrap test (10,000 replicates) are shown next to the branches (48). The tree is drawn to scale, with branch lengths in the same units as those of the evolutionary distances used to infer the phylogenetic tree. The evolutionary distances were computed using the p-distance method (49) and are in the units of the number of base differences per site. This analysis involved 21 nucleotide sequences. Prior to analysis, sequences were trimmed to include 22 codons before the codon for the first cysteine (CYS23) to 6 nucleotides past the last cysteine (CYS104). All ambiguous positions were removed for each sequence pair (pairwise deletion option). There were a total of 300 positions in the final dataset, and evolutionary analyses were conducted in MEGA X (40). Species are indicated by color, *Danio rerio* (red) and *Ictalurus punctatus* (blue).

Our annotation of *Danio rerio* chromosome 2 (accession number LR812064, see **Supplementary Table 5**) demonstrated that the zebrafish TRG locus only contained one cassette consisting of seven TRGVs, seven TRGJs and one TRGC (V_7_-J_7_-C). The TRGV genes form four subgroups. Subgroup 1 consists of three members and subgroup 4 consists of two members. Subgroups 2 and 3 have only one member each. The catfish and zebrafish TRGV genes share 31-60% nucleotide identity. Also, as expected phylogenetic analyses confirmed that catfish TRGV subgroups are more similar to zebrafish TRGV subgroups than to other catfish TRGV subgroups (**Figure 6**). For example, catfish TRGV subgroup 2 branches with zebrafish subgroups 1 and 3, and members from catfish subgroup 3 branch with zebrafish subgroup 4 members. In addition, the catfish TRGJ genes share from 35-67% nucleotide identity with the zebrafish TRGJ genes.

### Clonotype Analysis

**Figure 7:**
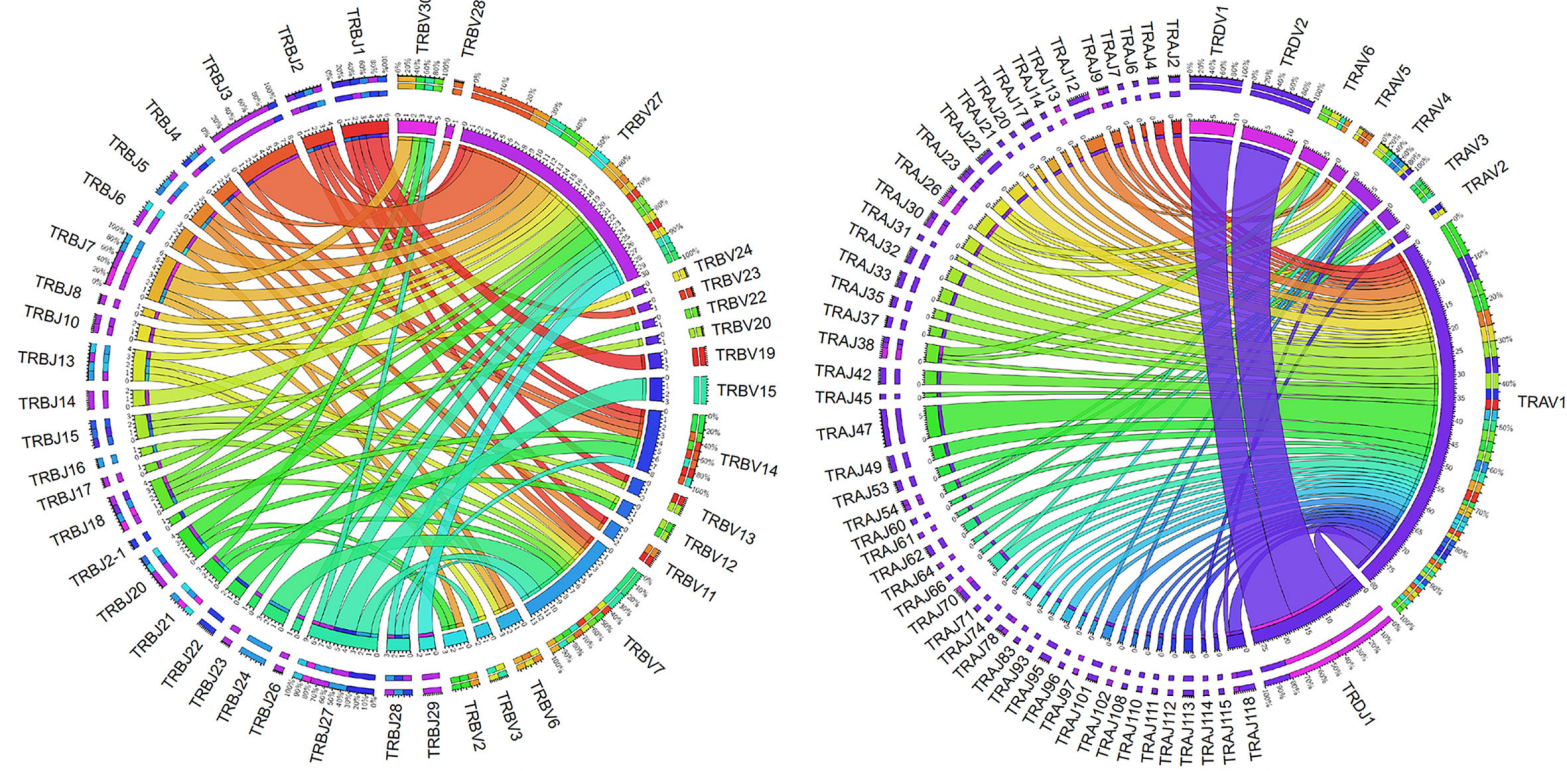
Catfish TRB and TRAD clonotype analysis. The TRB and TRAD clonotypes were mapped by V gene subgroup to their associated J gene. Chord diagrams were drawn using Circos based resolved parings of specific V gene subgroups and single J genes from TRA/D (n = 122) and TRB (n = 83) clonotypes. Labels along the edge of the inner circle provide the number of associations between any given TRJ gene and TRV subgroup pair. Tick marks represent values of 0.5 (clonotypes numbers represent integers only). Ribbons are colored according to the J gene (row in the table); and segment color interpolation was performed according to count. Outer labels of the chord diagrams indicate the relative percentages of total counts for each segment and are listed from largest to smallest.

As a proof of principle, and to begin to examine the diversity of the expressed repertoire and the usage of catfish TRV and TRJ genes, a database of the 267 TRV and 167 TRJ was created. This database was then used to analyze an available transcriptome obtained from pooled RNA from 19 tissues of a gynogenetic catfish (SRR392744; 50). Using this dataset, we identified 241 V CDR3 regions, which represented 105 TRA, 87 TRB, 31 TRD, and 18 TRG sequences. Out of 241 VDJ sequences, 90 could unambiguously be assigned to specific TRV and TRJ genes. In our analysis and with this limited dataset, we focused on the expression of subgroups rather than individual V genes and determined that 222 clonotypes could be assigned to TRV subgroups and specific TRJ genes (**Supplementary Figure S9**, **Supplementary Table 6**). For example, representative members from each of the six catfish TRA and two TRD V gene subgroups were identified. In contrast, for TRB only 17 out of 30 TRB subgroups were represented, and it is important to note that the 13 subgroups that were not found consisted of one or two members (**Figure 7**). Fifty-four TRAJ sequences (out of 120 functional TRAJ genes) and 26 TRBJ (out of 30 functional TRBJ genes) were also identified in this clonotype dataset. In addition, using NCBI searches we matched 12 TRAV cDNAs to individual TRAV germline genes from four TRAV subgroups, and matched 24 TRBV cDNAs to individual TRBV germline genes from 12 TRBV subgroups (**Supplementary Figure S10**). Interestingly, even though some of these cDNA sequences were from unrelated outbred catfish, these TRV genes only encoded very few amino acid differences.

**Figure 8:**
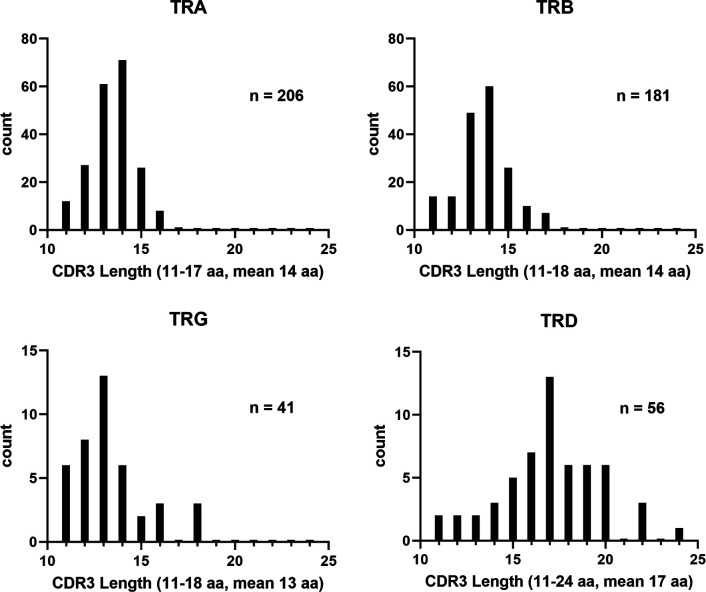
Distribution of catfish TR CDR3 lengths. Lengths of the CDR3 regions obtained from the clonotype analysis were graphed by isotype. The CDR3 regions were defined as the region spanning codon CYS104 to PHE118. N values represent the number of clonotypes included for each isotype.

In comparison, the TRD and TRG clonotypes utilized V genes from each of the two TRDV subgroups and each of the three TRGV subgroups, respectively. The single TRDJ was mainly expressed with TRDV1 and TRDV2, however five rearrangements with TRAV1 subgroup members were also identified. Previously, Moulana et al. (35), demonstrated that TRDV can rearrange to TRAJ genes, and we also found evidence for this type of TRDV to TRAJ rearrangement in this data set. However, due to sequence similarities at the 3’ end of these TRDV sequences, their subgroups could not be determined. Interestingly, only 18 TRG clonotypes were identified and five were from subgroup TRGV1; 12 were from TRGV2, and one was from TRGV3. The members from subgroup TRGV2 rearranged with TRGJ2, TRGJ3-1, and TRGJ3-6, while TRGV3 rearranged with TRGJ3-6. TRGV1 was expressed with either TRGJ1 or TRGJ3-2. We did not identify any rearrangements with TRGJ4-2. As a complement to this, we performed clonotype analysis on RNA-seq data from two unrelated outbred catfish. The results showed that TRV subgroups and individual TRJ segments could be resolved in 96% of the available sequences (**Supplementary Figure S9**). Comparisons of the clonotypes isolated from the three fish showed that only one TRA CDR3 sequence was shared among all the three fish (CALNTGGVNKIIF). From these clonotypes, we also examined the CDR3 lengths. While the TRA, TRB, and TRG CDR3 lengths were similar (11-18; mean 14), the TRD CDR3 lengths were longer and ranged from 11 to 24 (mean 17; **Figure 8**).

## Discussion

**Figure 9:**
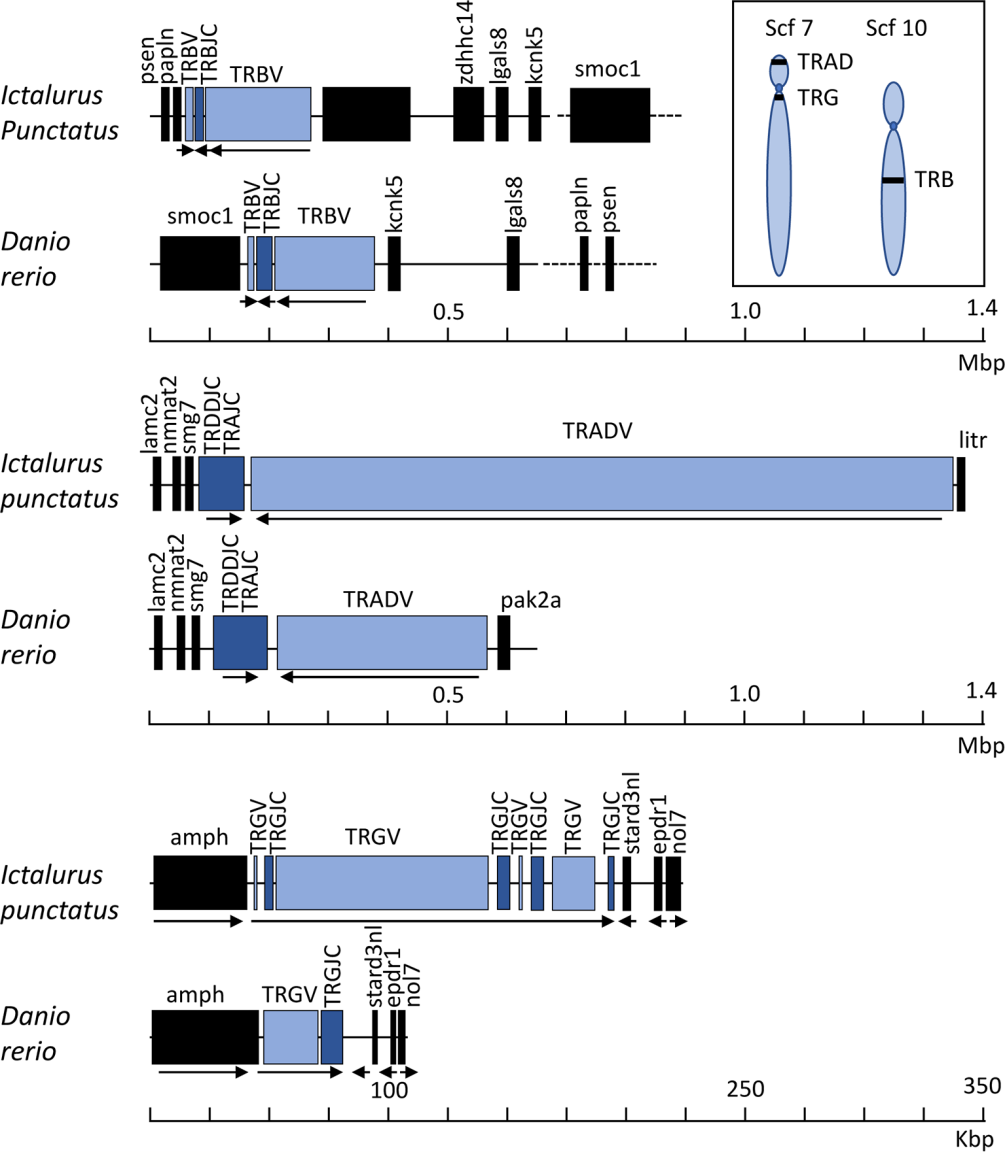
Germline representation of the channel catfish TRB, TRAD and TRG loci. Schematic arrays of the catfish TRB, TRAD, and TRG loci are shown compared with those of the zebrafish, *Danio rerio*. TRV gene segments are in blue, TRC constant region genes are in navy, and non-TR flanking genes are in black. Arrows show the direction of transcription within each of the TR arrays. Note that the scale bar for the TRB and TRAD loci span 1.4 Mbp, while the scale bar for the TRG loci spans 350 kb. A schematic of the approximate chromosomal locations of the catfish TR loci are at the upper right. In the catfish CCBL1 genome assembly, the TRAD and TRG loci are located on scaffold 7 and the TRB locus is present on scaffold 10. Zebrafish annotations were based on accession numbers: TRB: NC_007128.7; TRG: LR812064; TRAD: NC_007113.7. Schematics are drawn to scale.

Collectively, our goals for this annotation of the complete channel catfish TR germline repertoire were to create a valuable resource for catfish TR repertoire analyses after pathogenic challenge or immunization and provide a foundation for the characterization of TR genes in other teleost species. To expand our study, we chose to compare the catfish TR genes with those of the zebrafish. For this, we analyzed and annotated the TRB and TRG loci from the *Danio rerio* strain Tübingen, and reanalyzed the zebrafish TRAD data from Seelye et al., 2016 (22). Catfish and zebrafish are members of Siluriformes and Cypriniformes, respectively. These are the two largest orders within the superorder Ostariophysi, and together they represent approximately 8,300 teleosts species (57, 58). In 2018, based on time-calibrated phylogeny of 125 nuclear genes, Dai et al. estimated that the orders Siluriformes and Cypriniformes shared a common ancestor approximately 133 million years ago (59). This time frame has allowed for significant genetic drift, evolution, and selection of immune genes within polymorphic gene families such as the major histocompatibility complex (MHC), TR, IG, and leukocyte immune type receptor (LITR) families. Of these genes the TR genes seem to be the most conserved at the genomic level, however the degree of conservation shared between catfish and zebrafish differs among the three TR loci. For example, the genomic context of the TRG locus is the most conserved, i.e. the genes flanking the catfish and the zebrafish TRG, amphiphysin (*AMPH*) and STARD3 N-terminal like protein (*STARD3NL*) also flank the TRG loci in mammals, marsupials, birds, and reptiles (**Figure 9** (60–62);. Also, even though their genomic organizations are quite different, the catfish and the zebrafish TRVG subgroups display strong relatedness to each other. The zebrafish locus spans only 33 kb and contains one array consisting of seven TRGV genes, seven TRGJ genes and one C gene, while the catfish TRG locus spans ~151 kb and encodes four V-J-C cassettes. However, despite these differences, both the catfish and zebrafish TRVG subgroups form three inter-species clades linearly dispersed along the chromosome (**Figure 6**). Similarly, the catfish TRBV subgroups share 11 ancestor nodes with zebrafish TRBV subgroups (**Figure 2**). Taken together, these findings imply that at least three ancestral TRGV sequences and 11 TRBV sequences were present in otophysan fishes 133 MYA.

Interestingly, while the catfish and zebrafish TRB loci are similar in size (215 kb), the catfish TRB locus is more compact (**Figure 9**), i.e. there are 105 functional TRBV genes, 31 TRBJ genes and one TRBD encoded within the catfish TRB locus. Comparatively, in the zebrafish there are 51 TRBV genes, 31 TRBJ genes and one TRBD gene (see **Table 1**). Consequently, even though the catfish TRB locus encodes for approximately twice as many TRBV genes as the zebrafish TRBV, the catfish TRB repertoire is less diverse and consists of fewer subgroups. Thus, in catfish this less diverse repertoire is likely due to the multiple duplications and expansions of TRBV subgroups 2, 3, 7, 11, 14, 19, 27 and 30. Each of these subgroups encodes for six or more members (**Figure 3**). In contrast, the largest zebrafish TRBV subgroup consists of only five members and most of the zebrafish subgroups contain either one or two members. That both the catfish and the zebrafish TRB loci are flanked by homologous genes while their gene order is not conserved, implies that multiple translocation events have occurred in this region. Such events, catalyzed by double-stranded DNA breaks, are predicted to be influenced to a degree by both transcriptional activity and chromosomal location (63). In comparing the catfish and zebrafish TRAD loci, the genes 5’ of both the catfish and zebrafish TRDD gene segments include genes for laminin (*lamc2*), nicotinamide nucleotide adenylyl-transferase 2 (*NMNAT2*), and nonsense-mediated mRNA decay factor (*SMG-7*). These same genes are also found 5’ of the TRDD genes in Atlantic salmon (20). The gene flanking the other side of TRAD locus differ however in the three species. The 5’ TRADV gene in zebrafish is p21 protein (Cdc42/Rac)-activated kinase 2a (*pak2a*; 22), and in salmon it is cytochrome P450 family 7 subfamily B polypeptide 1 (CYP7B1, 20). Interestingly in the catfish, a gene located just 3,365 bp upstream of the first TRAV gene (TRAV1-1p) encodes a LITR, and it is important to note that this is the first documentation of linkage between one of the three catfish LITR loci and the TRAD locus. We also have determined that these loci (TRAD and LITR) are not linked in zebrafish.

**Table 1:** TCR variable (V), diversity (D) and joining (J) gene segment numbers in the channel catfish, *Ictalurus punctatus*, zebrafish, *Danio rerio*, and minifish, *Paedocypris progenetica*.

	Channel Catfish	Zebrafish*	Minifish	Humans
**TRAV**	108 (6ψ, 20 R)	149 (8 ψ)	52	54
# subgroups	6	53		
TCR alpha J	125 (4ψ)	111	61	61
**TRBV**	112 (6ψ, 1 ORF, 9 R)	51 (1 ψ)	11	64 - 67
# subgroups	30	36		
TCR beta D	1	1	8	2
TCR beta J	31 (1ψ)	31 (6 ψ)	9	61
**TRDV**	32 (6ψ, 15 R)	149 (8ψ)	52*	8
# subgroups	2			
TCR delta D	4	6	2	3
TCR delta J	1	2	2	4
**TRGV**	15 (2ψ)	7	3	14
# subgroups	3	4		
TCR gamma J	10 (2ψ)	7	2	5

*The TRAD locus is based on the reanalyzed sequences from Seelye et al. (22), which were trimmed to include 22 codons before the codon for the first cysteine that forms the disulfide bond (CYS23) and extends until 6 nucleotides past the cysteine (CYS104). The sequences were grouped based on ≥75% nucleotide identity. The TRD subgroups were not assigned, and the indicated numbers are the total number of TRV genes in the TRAD locus. The zebrafish TRB and TRG loci are annotated on the public sequences from the Danio rerio strain Tübingen, (accession # NC_007128.7 and LR812064).

ψ denotes the number of pseudogenes within the total gene number.

R denotes the number of remnants present in addition to the genes and pseudogenes.

The catfish TRAD locus is the largest TRAD locus described in any fish species to date, and it spans 1.3 Mb. In light of this, it was somewhat surprising that the 140 TRA and TRD gene segments encoded in this locus comprise only 8 subgroups. For example, Yazawa et al. (20), grouped 128 unique functional Atlantic salmon TRADV genes into 29 TRAD subgroups, and Seelye et al. (22), grouped 146 zebrafish TRAD genes into 42 subgroups. Both of these investigators, however defined their subgroups based on a less stringent definition, i.e. their subgroup members share greater than 70% nucleotide identity. It is likely that the limited diversity of the catfish TRADV genes is due to the many duplication events, and it is important to note that the catfish TRADV genes are embedded in longer gene segments of 4-12 kb that exhibit high similarity (96% nucleotide identity). Such similarity stretches of sequences are called homology units (64). Our dot plot analysis of the catfish TRAD locus demonstrated three main regions rich in homology units, and their presence suggests that in the catfish the expansion of TRAD subgroups occurred through relatively recent gene duplications (**Supplementary Figure S5**). These extensive duplications may have resulted from unequal crossover events facilitated by genomic repeats, e.g. transposons or LINE repeats, as first suggested by Glusman et al. for the murine TRAD locus (64). In comparison, homology units in the catfish TRB locus, are fewer, shorter, and not as well conserved (**Supplementary Figure S5**). The difference in the frequency of duplications events between the catfish TRAD and TRB loci may also be due to their chromosomal location. The catfish TRAD locus is located approximately 300 kb from the telomere on scaffold 7 while the TRB locus is present 1.8 Mb from the centromere on scaffold 10. In mammals, crossover events during meiosis are more likely to occur near the telomeres, and this is also likely true in fish (65). In comparison, the TRAD loci in zebrafish and Atlantic salmon are located 22 Mb and 20 Mb from the telomeres on their respective chromosomes. It is unknown if the duplication events, homogenization, and loss of TRA subgroups have any functional consequences for the TCRαβ repertoire, and cell mediated immunity in catfish.

Based on our complete annotation of the catfish TRB, TRAD, TRG loci we created a TRV and a TRJ database and examined if this database could be applied in clonotype analyses of available short-read Illumina RNA-seq datasets. The 3’ ends of catfish V genes within a subgroup are very similar, and unfortunately this precluded the assignments of most CDR3 regions to specific TRV genes. Even so from these preliminary analyses it appears that the expressed TR repertoire is based on the on the stochastic use of V, D and J gene segments, i.e. the most frequently represented TRV segments were from the largest TRV subgroups. Also, the TRA and TRB clonotypes represented 76-85% of the total number of identified clonotypes, while 15-24% were TRD and TRG clonotypes. This ratio is similar to the ratios of αβ to γδ transcripts observed in the minifish and consistent with the observation that 15% of the lymphocytes in zebrafish spleen, head kidney, and blood constitute γδ T cells (23, 66). Taken together this indicates that fishes are γδ “low” and fewer than 25% of their T cells express TRG and TRD.

Combined, our catfish TR annotation and clonotype data imply that different strategies are used for diversifying the TRA, TRB and TRD expressed repertoires. The diversity of the repertoire in TRB relies on the number of the TRBV subgroups and TRBJ genes, while TRA diversity depends on the large number of different TRAJ genes, which appear to be minimally trimmed. In contrast, TRD diversity is due to junctional diversity, i.e., nucleotide additions and the utilization of up to four TRDD segments, as previously described (35). This usage results in longer TRD CDR3 as compared to TRB, TRA, and TRG CDR3 regions. It is well known that the TRA and TRB CDR3 in human and mice are highly constrained, and of similar lengths, ranging from 6-12 amino acids (67). Recently, it was demonstrated in mice that this CDR3 length restriction results from thymic selection (68). Longer TRA and TRB CDR3 regions were found to alter the conformation of the TR and impair the contact between the TR CDR1/CDR2 and peptide bound MHC, which in turn led to reduced signaling. In this way, MHC restriction favors a TR repertoire of CDR3 that are shorter than 14 amino acids. In the future, it will be interesting to examine if similar constraints are applied to teleost TRA and TRB CDR3 through thymic selection and MHC restriction. It will also be important to perform targeted deep sequencing of the catfish TR repertoires to determine if the frequencies of shared or public clonotypes are similar to what has been observed in the zebrafish and minifish. Covacu et al. (69), defined public clonotypes as CDR3 sequences shared with at least one other individual and demonstrated that 36% of the total number of TCRβ1 CDR3 sequences are public. It was also shown that the TRBC1 repertoire in naive zebrafish consisted of a relatively low number of unique sequences (49 – 599 per individual) and that about 16% of these unique sequences were shared between different individual fish. The minifish is one of the smallest known vertebrates and its germline TRA and TRB repertoires consist of 51 TRAV and 61 TRAJ genes; and 11 TRBV, eight TRBD and nine TRBJ genes, respectively. Recently it was demonstrated that the available TRA and TRB repertoires in a single minifish range from 650-2,600 different clonotypes (n= 4; 23). In addition, the authors showed that 20% of the most highly expressed TRA clonotypes and 5% of the most frequent TRB clonotypes are shared between individuals. It is unknown if the high frequency of public clonotypes found in the zebrafish and in the minifish are adaptations to their small sizes and low numbers of lymphocytes, or if the environment and genetics influence the clonotype expression. The numbers of αβ T cells in minifish and zebrafish are estimated to be approximately 8 x 10^3^ and 1.6 x 10^5^, respectively. These small cell numbers make it possible to perform exhaustive TR repertoire analyses. Our clonotype analysis of catfish RNA-seq data was performed on total RNA and did not target TR sequences, therefore we only captured a fraction of the expressed clonotypes. For example, in our preliminary datasets we only found one TRA CDR3 sequence that was shared between all three fish and one other TRA CDR3 sequence that was shared between two individuals.

Overall, we believe that the complete annotation of the catfish TR genes presented here, will allow the catfish research community to perform in depth clonotype-based analyses, including the characterization of TR clonotypes before and after immunization or infection. Such studies will likely provide insights into the expansion of certain clonotypes associated with specific immune responses. In addition, these catfish and zebrafish TR annotations will contribute to the ongoing discussion of TR subgroup evolution and adaptations.

## Data Availability Statement

The original contributions presented in the study are included in the article/**Supplementary Material**. Further inquiries can be directed to the corresponding authors.

## Author Contributions

SQ prepared and provided the CCBL1 channel catfish genome assembly and verified the accuracy of the TR contigs. EB, MW, JC, and KF annotated the TCR loci. EB and JC created the figures. EB, MW, and JC conducted the phylogenetic analysis. JC and KS performed the clonotyping analysis. EB, MW, SQ, and JC prepared the manuscript. MW, EB and SQ supervised all aspects of the research. All authors read and approved of the final manuscript.

## Funding

This study was supported by grants NSF-IOS 1656419 through the National Science Foundation (https://www.nsf.gov/) and USDA-NIFA 2020-67034-31895 through the United States Department of Agriculture (https://www.usda.gov/). SQ is supported by funds from the USDA Agricultural Research Service in house project number 6066-31000-016-00D.

## Author Disclaimer

Mention of trade names or commercial products in this publication is solely for the purpose of providing specific information and does not imply recommendation or endorsement by the U.S. Department of Agriculture. USDA is an equal opportunity provider and employer.

## Conflict of Interest

The authors declare that the research was conducted in the absence of any commercial or financial relationships that could be construed as a potential conflict of interest.

## Publisher’s Note

All claims expressed in this article are solely those of the authors and do not necessarily represent those of their affiliated organizations, or those of the publisher, the editors and the reviewers. Any product that may be evaluated in this article, or claim that may be made by its manufacturer, is not guaranteed or endorsed by the publisher.

## Footnotes

1
http://imed.med.ucm.es/Tools/sias.html

2
http://mkweb.bcgsc.ca/tableviewer/
