# Supplementary material for: A Comprehensive Annotation of the Channel Catfish (Ictalurus punctatus) T Cell Receptor Alpha/Delta, Beta, and Gamma Loci
Source: Front Immunol. 2021 Nov 25;12:786402. doi: 10.3389/fimmu.2021.786402 (PMC8656973; doi:10.3389/fimmu.2021.786402)
Supplement: Supplementary file 1 [file DataSheet_1.pdf]

Deduced amino acid sequences of catfish TRBV subgroups

Subgroup 1
TRBV1-1 MNKLLRTFLLITSIHSLIWITVYAASTDVFQTPNDLLRKHGDSSELHCSHSISGYNTIQWYKQTRNTEIQFMGYLVGSQPQLEPEFRNMVTLTGNGYSNGTLTIKSLTPNDSAEYFCAAF
TRBV1-2 MNKLQYIFTLLITIHSLIWPAVFAASKEVFQTPHDLLRKHGESSEIQCAHKISGYDRILWYKQTQDREYLFMGYNFLSQSQLEPEFNNQMTLTGNGDSKGILTIKSLTPNDSAEYFCAAY

Subgroup 2 previously family 4
TRBV2-1 MIRILVFFQSLYWIQGVAGVNDVSQPNILWAKFGQSATINCSHTKGSSYNRMYWFRQHHGESMELIVYTGTFITADFGKFSQSKFSTIKTVAESGSFTVNDVDYNDSAVYFCAVSE
TRBV2-2 MIRVLVIFQSLYWIQGVAGENGVFQPSILWAKMGQSATINCTHTKGISYDHMYWYRQHHGESMELIVYTPTYGTHDFGKFNQSKFSAIKAVPASGSFTVKNVDYNDKAEYFCAVSK
TRBV2-3 MIRIIIVFQSLYWIQGIAGGDDVSQPNLISAKMGQSATINCSHTKGSSYYLMYWFRQYHGESMELIVLTNAFGTPDFGKFSESKFSAIQTVPESGSFTVKDVDYSDSAVYFCAVSK
TRBV2-4 MIRVLVFFQSLYWIQGVAGTKDVFQPSILWAKMGQSATINCSHSKDANHNQMYWYRQYHGESMELIVFTTSFGTQDFGKFSQSKFSAIKTVPESGSFTVKDVDYSDSAVYFCAVRE
TRBV2-5 MIRIIIVFQSLYWIQGVAGVNDVSQPSILWAKIGQSATINCSHTKGAFYNQMYWFLQYHGKSMELIVYTTSFGTLEFGNVSQSKFSAIKTVPESGSLEVKDVDYNDNAVYFCAVRE
TRBV2-6 MIRIIIIFQSLYWIQGVAGANDVLQPEILWAQFGQSVTINCSHTKGSAYREMYWFRQYQGESMELIVYTTSFGTPDFGKSDQKKFSAIKTVPENGSFTVKDVDYNDNAVYFCAVRE
TRBV2-7 MIWILMLVHSLNCIQGVVGANDVFQPNILWAEMGQSPTINCSHTKGVDYNKMYWFRQHQGESMELIVYTTSFGTPDFGKFDPNKFSAIKPNPESGSFTVKDVDNSDSAVYFCAVSK

Subgroup 3 previously family 5
TRBV3-1 MINVLIIIFPAILLCLAGSGVCSVVQNPPDFIKYPDEFAEIKCAHNVNNYDRILWYKHNQDTGFTLMGYHYTVSSMLEEEFGTKIKLSGDGSTNSSLTIKSLSVNGSAVYFCAAY
TRBV3-2 MCTVLII-VSATLLCLAGSAVFTVDQNPPDLIKYQNETVEMKCEHSVNTYDRMLWYKHSQDTGFKYMGYLNTIFPKEEAEFGTKIKLSGDGRKSGSMTINSLSVNDSAVYFCVAY
TRBV3-3 MHSVLII-FSATLLCLPGSGVCSVVQNPPDLIKYKDEFAEIKCAHTVNNYDRILWYKHNQDTGFTHMGYLYTLSSTLEEEFETKIKLSGDGRNNGSLTIKSLSVNDSAVYFCAAY
TRBV3-4 MCTVLVI-FSATLLFLPGSGVCSVVQSPPDVIKNQDELAEIKCAHTVKNYNQILWYKHSQDTGFKYMGYLSNAYPKLEEEFETKIKLSGDGSKNGSLSIKSLSVNDSAVYFCAAY
TRBV3-5P MHTGLIT-FTATLLCLPAS-LFSVVQSPPNVKK*QGEFAEIKCAHTVNSYNQILWYKHSQDTVCTLMGYLYNAFFNAGGRIWNQNQTEW*WEHKRFFDHKESFSEWQCCVFLCSLL
TRBV3-6 MVTALII-TILFYFT--DSTVSAVLQNPPDLIKNQNEFVELKCAHTVKSYNRILWYKRSQDTRFTLMGYLFNNSPRLEADIKMKINLTGDGRNNGSLTIDNVSVNDSAVYFCAAYF

Subgroup 4
TRBV4 MIRSINSASVTLLDSSGLTSGMVVEQTPPDLIKNQGESAKIRCSHKIEKYDRIMWYKETKNKEFIFMGYLISKNANPETDFKDKIRIGGDAFDGFGSLEVINLSLKSEGVYFCAAY

Subgroup 5
TRBV5 MIKVQIRFIVSLLWLTDAVFSSKVQQSPSDLIQSRGDTVELKCLHSIESYNIILWYKSNASHTFELMGYLWNKKDYVEPNFTEKIELKGDASKNGALIIKTLSSDDSTMYFCAASF

Subgroup 6
TRBV5-1 MKVHILIIILMVLHAVLGQQQNYEIHQSPSKLMTFPGENTELHCKHSVSLFYTILWYQQSLNDISMKLIGYVYYRQVNVENSFKSHFNVSGDGQKQSTLHLVKVRAAEHSAVYYCAASR
TRBV5-2 MRTHIAIILTVLHLILGQQQNYDIHQSPSEVLSFPGENTELYCKHSVSAFSMISWYRHTFKDTSMQLIAYVRYKSGNVESPFVGYFNISGDGEKHSTLHLVKLRAAEHTGVYYCAASR
TRBV5-3 MKAHTVVILTVLHLILGQQQSYDIHQSPSDVLSFPGENPELQCKHSVSSFYMISWYRQAFTDTSMQLIAYVRYESANVETPFSSQFNVSGDGAKHSTLQLVKLRAAEHTGVYYCAASR
TRBV5-4 MKSHTVVILTILHLILGQQQSYDIHQSPSDLLSFPGENTELQCTHSVSTLNMILWYQQSFNDTSMKLIAYVRYKTANVETPFSSHFNVSGDGQKHSTLHFIKLRAAEDTAVYYCAASR
TRBV5-5 MWTHVLILLTVVHSVLGQLQKYSIHQSPSDLVKFPEENTEVHCKHSVSEFYVMMWYQQSLNNTSMKLIAHLYFKQATVENSFSSHFNVSGDGEKHSTLHLIKLTEAEHSAVYYCAASR

Subgroup 7
TRBV7-1 MIKFPCKFILFLWCFSGSLICKDVNQSPPDILCLPEENVTLSCIHGIFNYETILWYQRTQGDTSLKLIGYVRLTSAIEIEKDFQGHFSVAGDGRSSVSLQIPKARQVPHNALYFCAAY
TRBV7-2 MIRFSYKFIISVCFLSGSLICKDVHQSPADVLCLPEESVTLTCNHSIPDYYTILWYQRTQEDTGLKLIGYVYTTNPKY-EGNYEGNFTVSGHGSSSVSLQIPKARQVLHSALYFCAAS
TRBV7-3 MIRFSYKFIISVCFLSGSLICKDVHQSPADVLCLPEESVTLTCNHSIPSYYTILWYQRTQDDTGLKLIAYISNTSPQY-EGNYEGNFTVSGNGRSSAFLQIPKARQGLHSALYFCAAYY
TRBV7-4 MNMIRFSYKFIISVCFLSGSLICKDVHQSPADVLCLPEESVTLTCNHSIPKYNTILWYQRKQEDTDLKLIGYVYITSPQY-EGNYEGNFTVSGDGRSSVSLQIPKARQGLHSALYFCAAY
TRBV7-5 MTRFPFNLIICVSCLSGSFFCKDVHQSPADVLCLAEESVTLTCNQSMLTYNTILWYQRTHGDTGLKLIGYVYYTITKQVEKDYEGNFTVDGDGESSASLHIPKARQVLHSALYFCAASY
TRBV7-6 MIRFPLKLIICVSCLSGSLICKDVHQSPADVMCLPEEGVTLTCKHSIPSYNTILWYQRTHGDTNLKLIGYVRFTSAQI-EKDYEGNFTVSGDGRSSASLQISKATQVLHSTLYFCAAYY
TRBV7-7 MIQVSYKFIISVCFLSGSLICKDVHQSPADVLCLSEESVTLTCNHSITNYDTILWYQRTQEDTGLKLIGYVYATSPKY-EGNYEGNFTVSGNGRSSASLQIPKARQVLHSALYFCAAYY
TRBV7-8 MIRFSYNFIISVCFLSGSLICKDVHQSPADVLCLPDESVTLTCNHSIPNYNTILWYQRKQGDTGLKLIGYVYYTKPEY-ENNYEGNFTVRGEGKSSVPLQIPKATQVLHSALYFCAAS
TRBV7-9 MIRFPFKLIFCVSYLSGSLFCKDVHQNPTDVLCLPEESVTLTCSHSIPSYDRILWYQRTRGDTSLKLIGYVYYTNPNY-EGNYEGNFTVSGNGQSSVSLQIPKARQGLHSALYFCAAYY
TRBV7-10 MIRFPFKLIICVSCLSGSLFCNDVHQSPADVLCLSDESVTLTCNHSISSYNTILWYHRTHGNTSLKLIGHVFANTPNH-EKNYEGKFTVSGDGRSSVSLQIPKARQGLHSALYFCAAYY
TRBV7-11 MIGFSFKLIICVSCLSGS-FSKDVYQSPADVLCLPGESVTLTCNHSINNYDTILWYQRTHVDTSLKLIGYASYSHTKKIEDNYNGNFTLTGNGESSVSLQIPKATQVLHSALYFCAAYY
TRBV7-12 MIRFSIKLIICVSCLSGSLFCNDVHQNPADVLCLPEESVTLTCKHSSSSYNKILWYQKTHEDTSLKLIGYVYANNPKY-EGNNEGNFTVSGDGRSSVSLQIPKARQGLHSALYFCAAS
TRBV7-13 MIGFSFKFIICVSCLSGS-FSKDVYQSPADVLCLPGESVTLTCNHSIDNYDTILWYQRTHVDTSLKLIGYVRYSSTKKIEDNYNGNFTLTGNGESSVSLQIPKATQVLHSALYFCAAYY
TRBV7-14 MITFPFKIIICVTFLTGSLVCKDVHQNPTDVLCLPEESVTLTCKHSIPNYDTILWYQRTHGDTHLKLIAYVYYGNPKY-EGNYEGNFTVSGDGRSSASLQISKATQVLHSALYFCAAYY
TRBV7-15 MITFPFKLIICVTCLTGSSICKDVHQNPTDVLCLPEESVTLTCKHSIPNYNTILWYQRTHGDTHLKLIAYVFYTTPKY-EGNYEGNFTVSGDGQSSASLQISKATQVLHSALYFCAAFY

Supplementary Figure S1

Subgroup 8

TRBV8P MFQAFLKLTSLVLIVIGKMVQQTPHDLFTSQGELAEIKCSHDIQGHDRILWYRQSHNKELTFRISSI*IRKHRTKI*RQNHNQWKSKRVLQLSELDKHYTREWWSLFLCSIL

Subgroup 9

TRBV9 MTGIILLTLFLPAGFSLRQSPDLSAYEGQNVTLQCTQIGSSYNGMYWFRQRPSESLEPIVFYYVNMGTLEDRFQQKVSAIRKDNSLDLTVKELQSTDSGIYFCAKQD

Subgroup 10

TRBV10 MMILLFLSQIMLAGLVASHRVEQSPPDMIKNYTDSAQLYCSYVVKNFEHILWYKQSKERNLIYLGYNNLKYTYSEQDTKINLDDDMTNNATLTINNLIAKDSTVYFCAIQI

Subgroup 11

TRBV11-1P MISVNIFLPFLLLWTPVLAGENGVSQNPSVAWHLKGESAEMKCSHNKGAS-YFQMYWYRQRQGESMELIVYTTPSSEPDFG**K*IFNC*KNCCKWIINCEGSGYRGQRCIFLCSK*
TRBV11-2 MISVFIFLPFLLLWTPVLTGENGVSQNPSVAWHLKGESAEMKCSHNKGGT-YNQMYWYRQRQGESMELIVYTTASSEPDFGLVDKNKFSTVKKLATNGSLTVKDLDTEDSAVYFCAVSE
TRBV11-3 MISVIIFLPFLLLWTPVLTGENGVTQSPSVAWHLKGESAEMKCSHNKGAE-YYQMYWYRQRQGESMELIVYTTASSEPDFGSVDKNKFSTVKKLATNGSLTVKDLDTEDSAVYFCAVRE
TRBV11-4 MISVIISLTFLLYWKSVLAGENGVTQNPSVAWHLKGESAEMKCSHNKGSG-YYQMYWYRQRQGESMEFIVYTTTS-ESDFGSVDKNKFSTVKKNAANGSLTVKDLDTEDSAVYFCAVKE
TRBV11-5 MISVIISLTFLLYWKSVLAGENGVTQNPSVGWHLKGESAEMKCSHNKDAG-YFQMYWYRQRQGESMELIVYTTTS-ESDFGAVDKNKFSTVKKIAANRSLTVKDLDTEDSAVYFCAVKE
TRBV11-6 MISVIISLTFLLYWKSVLAGENGVTQNPSVGWHLKGESAEMKCSHNKGSG-YYQMYWYRQRQGESMEFIVYTTTS-ESDFGTVDKNKFSTVKKIAANGSLTVKDLDTEDSAVYFCAVKE
TRBV11-7P MISVIISLTFLFYWTA-LSCFTGLQV*YNLQTEEEL*I*QSHYLK*DAGYMYYQMYWYRQRQGESMELIVYITATSEEDF-S*-KINF------LLLEN*PQKELGTEDNAVYYCAVKE
TRBV11-8 MISVIISLTFLLYWTPVLTGENGVTQSPRVAWHLKGESAVMKCSHNKDAT-YYQMYWYRQRQGESMELIVYTTPSSEPEFGSVDKNKFSTVKKIAANGSLTVKDLDTEDSAVYFCAVKE

Subgroup 12

TRBV12-1P MMMIIITFTTSVICHAGWSSAVNDVHQTPPDLIKTIQESTDINCSHAIPNNELILWYKQSDSNHVQFLGYLNLNFPYPEDSLITKIDLDGDGRNKSRNLPSRICSQMTVQFISVRHV
TRBV12-2 MNIVLITLTALFISDIGRSSAAKDVHQTPTDLIKDIQESTNLSCSHAITSHQVMLWYRRSENKQLQLLGYLNLKFPYPEDSLKAKIELHGDGNNEGKLTIKNLQPDDSAVYFCAVKQ
TRBV12-3 MSIVLLIVTALFISDIGRSSAAKDVHQTPPDLIKNIQESTDLSCSHSIPNHEFMLWYKRSENKQLQLLGYLNSKFPYPEDSLKAKIELHGDGNNEGKLTIKNLQPDDSAVYFCAVRL
TRBV12-4 MITALVIFTASFICHIGRSSAAKDVHQTPTDLIKDTQESTNLSCSHAIPSHQVMLWYRRSENKQLQLLGYLNSKFPYPEDSLKAKIELHGDGNNEGKLTIKNLQPDDSAVYFCAVRL

Subgroup 13

TRBV13 MIISLFAFTASFICHTGWSSAVKNVHQTPPDLIKNLQQSTGLNCSHSIPGYDVMLWYKQSDSDELQILGHLNMHLKNPEQSLTNKISLDGNGENTGKITIKNLQLNDSAVYYCAVRR

Subgroup 14

TRBV14-1 MIRAASILWVLMICHRGLSQSNLVFQTPPDLFGNHKQSVKIQCEHSVPNYNQINWYRETQDQGLTLIGYQYGTSSPQIENDFKLKVEIAGDGNKNVSLTIKNLSSNDSVVYFCAAS
TRBV14-2 MIRAASILWVLMICHRGLSQSNLVFQTPPDLFGNHTQSVKIQCVHSVKNYDQINWYKETQDQGLTLIGYQLRKSSPQIENDFKLKVEIAGDGNKNVSLTIKNLSSNDSVVYFCAAY
TRBV14-3 MIQAASILWVLMICHRGLSQSNLVFQTPPDLFGNHTQSVKIQCVHSVKNYDQINWYKETQDQGLTLIGYQLRKSSPQIENDFKLKVEIAGDGNKNVSLTIKNLSSNDSVVYFCAAY
TRBV14-4 MIQAASILWVLMICHRGFSQSDLVFQTPLDLFGNHKQSVKIQCEHSVPSYNQINWYRETQDQGLTLIGYQVGKSSPQIENDFKLKVEIAGDGNKNVSLTIKNLSSNDSVVYFCAAY
TRBV14-5 MIRAASIIWVLMICPRGFSQSDRVFQTPLDLFGNHKQSVKIQCVHSVPNYNRIIWYKETQDQGLTLIGYQVGKSSPQIENDFKLKVEIAGDGNKNVSLTIKNLSSNDSVVYFCAAY
TRBV14-6 MIRAASILWVLMICHRGFSQSDLVFQTPPDLFGNHKQSVKIQCEHSVPSYNQINWYRETQDQGLTLIGYQYRTSSPQIENDFKLKVEIAGDGSKNVSLTIKNLSSNDSVVHFCAAS

Subgroup 15 previously family 6

TRBV15-1 MVTALLILALCLFIGQVDGSGVFQMPDIIWGSPGNSVEMNCSHNKGINYRQMYWFKQLPGEGITLLVFTSVGGIPDYGKFSKDKYEAIKTVVESGSLTVKTLDQGDDALYFCAVSQ
TRBV15-2 MITALLILALHHFSGQVDGSGVFQMPDIIWGSLGNSAEMNCSHNKDITYRQMYWFKQLPGEGITLLVFTSVGGEPDYGKFSKDKYEAIKTVAESGSLTVKTLDQGDDALYFCAVSQ
TRBV15-3 MITALLILALHHFSGQVDGSGVFQMPDVIWGSPGSSAEMNCSHNKDINYRQMYWFKQLPGEGITLLVFTSVGVEPDYGKFSKDKYGAIKTVVESGSLTVKNLDRGDSALYFCAVSQ

Subgroup 16

TRBV16-ORF MIDFKHLILYLVLTGLSMAIHVNQSPSHMTKTTEDSAEINCSHDNPVYDR-ILWYKHSDQRFKLIGNLYATFPSIVPIYGGNIKVDRDAVKECSLTIRNLSSADTGLYFLRCQK

Subgroup 17

TRBV17P MIRTIIVIITAFVIFMTGMSLSFTVHQKPPDLLMNEHDIVKLECSQHVPNYNVILWYKQFGD*ALQLLGYLYRNNINIEHLNREIKLDASDAFLIMENVSTSDSAVYYCAART

Subgroup 18

TRBV18-1 MIFRARATYFLLHWLIESCMCLSVSQFPAVIFLQPGELLKITCSHHDKKYDQIYWYIWQVNQQSLEHIGFLNFKNPQIDKKGFNISGDAEKEGYLTAQSVTADYSAMYFCAVS
TRBV18-2 MMLRNSATYFLLHWFIGSCMCLSVSQFPTVIFLQPGESIMITCSHHDKNYDKIYWY-QQVNQQNLELIGFLNFKNPQIDKKGFNISGDAEKEGYLTAQSVTADYSALYFCAVS

```
Subgroup 19
TRBV19-1   MAKIIILSIILLCLKATAISSLNIQQSPEHLLLKPEQNEAKLNCRHGDTNYPYMYWYQQKTVGGLVELIGMLHYEKFTPEEKFKARFNISGHSKGDAFLLISSITTEDSAVYFCAASK
TRBV19-2   MAKIIVSSIILISFKAAVISSLNIQQTPQRLLMTTEQTEAKLKCHHGDASYPYMYWYQQKTVSDSIELIGMLQYGTSTPEEKFKARFNISGHATGDAFLLISSITTEDSAVYFCAASK
TRBV19-3   MAKIIVSSIILISFKAAVISSLNIQQTPQRLLMTPEQIEAKLKCRHGDTNYPYMYWYQQKTVSDSIELIGMLQYGISTPEEKFKARFNISGHATGDAFLLISSITTEDSAVYFCAASK
TRBV19-4   MAKIIILSIILMSHK--VIFSLNIQQSPKHLLLTPEQNEAKLNCRHGDTNYLNMYWYQQKAVGGLVELIGMLQYGRSTPEEKFKARFNISGQSTGDAFLLISSITTEDSAVYFCAASK
TRBV19-5   MAKIIVSSIILIGFKAAVISCLNIQQTPQRLLMTPEQIEAKLKCRHGDTNYYYMYWYQQKTVSESIELIGMLQDGRSIPEEKFKARFNISGQSTGDAFLLISSITTEDSAVYFCAASK
TRBV19-6   MAKIIVSSIILISFKAAVISSLNIQQTPQRLLMKPEQIEAKLKCRHGDTNYYYMYWYQQKTVSDSIELIGMLQYGASTPEEKFKARFNISGHSTGDAFLLISSITTEDSAVYFCAARK
TRBV19-7   MAKIIILSIILMSHKAAVIFSLNIQQSPKHLLLTPEQNEAKLNCRHGDTSYPYMYWYQQKAVGGLVELIGMLQYERSIPEEKFKARFNISGHSTGDGFLLISSTTTEDSAVYFCAASK
TRBV19-8   MAKIIVSCIILISLKATVLSSLNIQQSPKHLLLTTEQSEAKLSCRHGDTSYQYMYWYQQKAVGGAFDLIGMLLFERSTPEEKFKARFNISGHSTGDAFLLISSTTTEDSAVYFCAASK
TRBV19-9   MAKIIVSCIILISLKATAISSLNIQQSPKHLLLTPEQNEAKLKCRHGDTNYQYMYWYQQKAVGASFELIGILVYGRSTPEEKFKARFNISGQSTGDAFLLISSITTEDSAVYFCAARK

Subgroup 20
TRBV20     MLIIRLVILQPLLLMLLTASVLSKVVFQTPPDLLKTLEDTAVLHCSHNVTGYNRILWYKMSSSGSEMKYLGNLFLETPNPEESRFTLSGDGRSKGSLSIPTLSLQDRAVYFCAAYL

Subgroup 21
TRBV21     MAHYVLIVHFVLLWFTDRSLSSKIIQTTDVIENSGNNVNLSCTHTYKDFFYLFWYQQTRQDTSLKLTGYLYTTTFNKETDYEKRFHIYGDAKSEGILQISNLNSHDSAVYFCAVRE

Subgroup 22
TRBV22-1   MSDLLYGLFILLKLLSNLSECVRFEQPSDLIANLTDNVKISCKHDDKNLYVMLWYQQRRESTTMALISYNYDTATPNYEAGYT-TRFEHNRIDTVTGDLTISNLNLSDSAVYYCAARM
TRBV22-2   MLTNMCSLFKSFSLLHCLSDCVQFEQPSDLIVNLKDNVKISCKHNDNNLDVMLWYHKWRESTTMALIGYSYATVPPNNEPGYPDTRFKQTRIDTVTGDLTISNLNLSDSAVYYCAAKL

Subgroup 23
TRBV23-1   MTGTLIYTLFFILLKGSSKGVLITQWPKYISSFKSTSVDMHCYQNDTDYQYTYWYRQIEGKEPVLIATYIARSPSYETGFGKGFKVWGSETKKWSLTVDVEEDSDAVYLCAANF
TRBV23-2   MTGTLLYILLFILMKGSCMGVLITQWPKYISSFKSTSVDMHCYQNDTDYDYTYWYRQIEGKEPVLIARYVAGSPIQEKGFENGFKAWG-TKKKWSLTVDVEEDSDAVYLCAASF

Subgroup 24 previously family 3
TRBV24-1   MYANVSKLCLFLYLFTGRTNCANIQQSSSLLVKETQNVTIQCSHENNNLYVMLWYQQKTNGGMALIGYSYGMTEPKNEEDFKDRFEQSRQSIMAGKLTISKVLQSDSAVYYCAARE
TRBV24-2   MYTIVCMLCLLLLLFIGRTNCAKVQQSSSLLVNDTQNVTIHCSHDDGDLDIMLWYQQKINSEMALIGYSYGMNEPNNEDNFKDRFKQSREDTQNGKLTISNVLQPDSAVYYCAARE

Subgroup 25
TRBV25     MYCMTYALGFYLIVFSTMVDSVNFQQDPHLIVSQTAKAKISCSHNDRTLTVMLWYRQERASTHLTLISYGYSTGTPFYEPGFNERFEMTRQDALSGELSISNPGLSDSAVYYCAASA

Subgroup 26
TRBV26     MSRLFEFFLFFSLFFGRASCVKVQQIPRSLIAKEQDNIMIQCSHDDSSLLLMLWYQQKSDSTDFSLITYAYGTGQATNEDGFKDRFKLSKESGLKGNLTISNLLQSDSAVYYCAASK

Subgroup 27 previously families 1 and 2
TRBV27-1   MYGTCGLCVFFILFF-ERTNCVKFLQTPSLIENESSNVTIQCSHDDSSLPRMLWYQQNSRTVMALIGYTAGASSDPNYEDGFKDRFKQSRQGTLNGSLTISNLRQSDSAVYYCAASM
TRBV27-2   MYGACGLCVVFILLF-ERTNCVKFEQTPSILANETSEITIQCSHDDSSLNAMLWYQQNSRTVMALIGYTAGASSDPNYEDGFKDRFKQSRQGTLNGSLTISSLRQSDSAVYYCAASM
TRBV27-3   MYGACGLCVVFILLF-ERTNCVKFEQTPSILANETSEITIQCSHDDSSLYTMLWYQQNSRTVMALIGYTAGASSDPNYEDGFKDRFKQSRQGTLKGNLTISSLRQSDSAVYYCAASM
TRBV27-4   MYGTCGLCVFFILFF-ERTNCVKFLQTPSLIENESSNVTIQCSHDDSGLTRMLWYQQNSRTIMALIGYTVGASSDPNYEDGFKDRFKQSRQGTLNGSLTISNLRQSDSAVYYCAASM
TRBV27-5   MYGACGLCVVFILLF-ERTNCVKFEQTPSILATETSEITIQCSHDDSSLYYMYWYQQNSRTVMALIGYTARASSDPNYEDGFKDRFKQSRQGTLNGSLTISNLRQSDSAVYYCAASM
TRBV27-6   MYGACGLCVVFILLF-ERTNCVKFEQTLSILATEASDITIQCSHDDSNLYNIYWYQQSSLAIMALIGYTVGANGDPNYEDGFKDRFKQSRQSTLKGNLTISSLRQSDSAVYYCAASM
TRBV27-7   MDGFYELFFVIILFF-VRANCVKFEQIPSVLANETGDVTIQCSHDDSNLEVMLWYQKKISTVITLIGYTYGAAGEPNYEDGFKDRFKQSRQNTLAGTLTISKLLQSDSALYYCAARK
TRBV27-8   MDGSCELIVFFILCF-GRINCVKFQTISSLLVNETEEVTIQCSHNDNTLQTMLWYLQNSNTVMALIGYTYTATSKPEYEDGFNVRYKQSRKSITEGSLTISKLLQSDSAVYYCAARM
TRBV27-9   MNCTCGLCVVFILFF-GRTNCAKVQQTPSILVNEKENITVSCSHNDNNLDRMLWYRQNSRTVLALIGYTMTAKSDPKYEEEFNDRFTLSRQGTLAGTLTISNLRQSDSAVYYCAASQ
TRBV27-10  MNCTCGLCVIFILLF-GRTNGVTFQQTPSLLVNETENITVNCSHNDNSLDRMYWYRQNSHTVLALIGYTLSAMSDPKYEEEFNDRFKLSRQGTLAGTLTISNLRQSDSAVYYCAASK
TRBV27-11  MYGICGLCVLFILFF-GKANCVTFQQTQSTLVNETENITIDCSHDDTNLDSMLWYQQNSRTVMALIGYTFTEASVPKYEDEFTKRFKLSRQGRTKGTLTISNLHQSDSAVYYCAARK
TRBV27-12  MYGLCELCVFFILIV-GRVNCVNFQHIPPLLVNETKEVIIQCIHDGTNLNNMLWYQQKSISSMGLIGYIYGATGSPNYEDGFKDRFKMSRKSITEGSLTISKLLQSDSAVYYCAASQ
TRBV27-13  MIDTCELCVFFILFF-GNANCVTFQQSPSTVGGETENITIDCSHDDTNLDCMLWFQRNSSTVLSLIGYTFTDMSDLMYEDELNDRFNLSRQGRTKGSLTISNLHQSDSAVYYCLLL
TRBV27-14  MSFLFFFFFFFW---GKANCVTFQQTSLILANETGDITISCSHDPSTLPVMLWYQQKSHTVIALIGYTTGITSDPNYENGFKDQFNLSRKSITEGSLTISNLRQSDSAVYYCAASQ
TRBV27-15  MYGTCGLCVFFVLFLAGKANCVKVEQTPLLLANETSDVTIHCRHDDGTLPVMLWYQQNSKTVMSLIGYTSGASGDPNYEDGFKDRFNLGRKSTTEGSLTISNLGQSDSAVYYCAARK
TRBV27-16  MVGAYEHFVLFILVF-GRANCVKIEQISSLLANETGEVTIQCSHDDNNLEVMLWYVQNSRTVMALIGYTNTAIGQPNYEDEFNDRYKQSRTSMTEGSLTISKLLQSDSAVYYCAASQ
```

Subgroup 28

```
TRBV28     MKMFSVGMLVLLLLHTCETLQVLQEQSSVMAIAGSAMSFHCTIDARFRMSTYTMQWYRQAYYGAPVQFLMMEYEQATKKMNVAMLQAENKFSLHISDLTLQDNGIYYCAA*
```

Subgroup 29

```
TRBV29     MGLEYVGFALLIITKSLHGVSIIQNPTLLIKKGGESVEIVCQHDDTSHYYMYWYRQRSLGEMDMITMSFGKDMAQTVEPYNESKYSMIRTEVKHSTLQLKALEANDSAVYFCASS
```

Subgroup 30

```
TRBV30-1   MACLHVILYFVTIDWLFTNCQQLIQVQQEPDDLVLSPGSSVKVSCAITGTNNPDLFWYRWNEAAGFVLVFSSRGAGMMDPVSEGQFKSNRPTDLQMVLESEGLSEIGSAVWYCAASP
TRBV30-2   MRCL--ILYILTIQWLLTSCVQVIQVHQEPENLVLSPGSTLKISCSITGIDDPYLYWYHWNETAGFTLVFSSIATGSVNPASDGQFKSYRHEDNRIILGSDGVSEIGSAVWYCAASP
TRBV30-3   MMHLHLSLFFLTVHWFSNICMQLIQVQQKPRDLVLSKGSSLKISCSITGTANPYLYWYHWNETAGFTLVFTSLATGSVNPASDGQFKSHRPDWLQIILESDGVSEIGSAVWYCAASP
TRBV30-4   MRSLCLILYILTVQWLLTSCVQVIQVHQEPENLVLSSGSSLKISCSITGTATPYLYWYHWNETAGFTQVFTSVTTGSVNPASDGQFKSQRPDWLQIILESDAVSEIGSAVWYCAASP
TRBV30-5   MMHLHLSLFILTVHWFSNICMQLIQVQQDKRDLVLAQGSSLKVSCSITGSSIPNLYWYHWNKTAGFTLVFTSVTTGSVNPASDGQFKSHRPEQRQIILESDGVSEIGSAVWYCAASS
TRBV30-6   MMHLHLSLFILTVHWFSNICMQLIQVQQDTRDLVLAQGSSLKVSCSVTSMSIPNLYWYHWNETAGFTLVFTSVATGSVNPASDGQFKSHRPEQRQIILESDGVSEIGSAVWYCAASS
TRBV30-7   MMHLHLSLFILTVHWFSNICMQLIQVQQDTRDLVLSQGLSLKVSCSITGLSIPTLYWYHWNKTAGFTLVFTSFATGSVNPASDGQFKSHRPDWLQIILESDAVSEIGSAVWYCAASS
TRBV30-8   MRHLCLSLCILTVQWLLSGCQQIIQVHQEPEDLVLSPGSSLKISCSITGISNPNLYWYHWNESTEFTLVFTSVGTGVMNPASKGQFKSHRPKDLQMVLESEAVSEIGSAVWYCAARS
TRBV30-9   MMHLHLTLFILTTNWLSTTCLELIQVHQDLEDLVLSKGSSLKVSCAISGTNDPYLYWYRWTPAEGFTLVFYSISAGSVNPSSEGQFKSHRPDKLHIVLESEGLNEIGSAVWYCAASP
```

A.

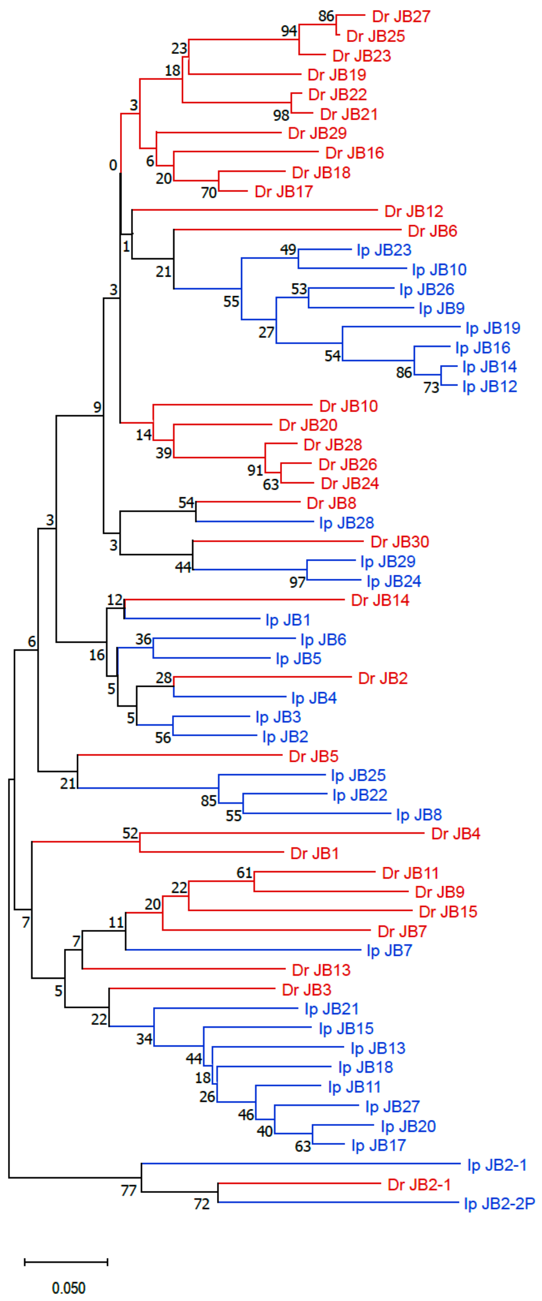

B.

IpJ2-1 **AGTTTATGT**CAGGCCTTGTGT**CAGCGTG**--TCAGCTTCGCCGACTTGAAATTTGGGCCTGGGACGAGACTAACAGTTTTAG/gt

IpJ2-2P **CATTTCTGT**GTTGAGTTAAAT**CAACGTA**TACTAGTTATGCAAAAGTGGAATTTGGACCAGGGACCAAGCTAACAGTCCCGG/ct

DrJ2-1 **GGTTTAGGT**AATGAGATAAAT**CATCGTG**TACTAGCTACGCAAAACTAGAATTTGGCGAAGGAACCAAACTGACAGTTTTGG/gt

IpJ1-28 **GTTTTCTCT**ATAGCATGATAT**CAGTGTG**-AACTACAACAACCCAGCATACTTTGGCAGTGGCACCAAACTCACAGTGATAG/gt

DrJ1-8 **GTTTTATCT**ATGGCATAGAAT**CAGTGTG**-AACAACTACGATCCAGCATACTTTGGTAGTGGCACCAAACTCACAGTCCTAG/gt

DrJ30 **GGTTTTACT**GTAACTGTTCAA**CAATGTG**----AACAACAGACCTGCATACTTTGGTGACGGGACCAAACTTACTGTTCTGG/gt

IpJ29 **TGTTTTACT**CTTATTGAAATT**CAGTGTG**----AACAACCAGCCTGCATACTTTGGCCAGGGGACCAAACTCACAGTTCTCG/gt

IpJ24 **AGTTTTCAT**CTTACTGCTGTT**CAGTGTG**----AACACTCAGCCTGCATACTTTGGCCAGGGGACCAAACTCACAGTTCTTG/gt

Supplementary Figure S3

Deduced amino acid sequences of catfish TRAV genes

Subgroup 1 previously Family 1 and Family 4

```
                            1   FR1              23     CDR1    41   FR2          CDR2                        89    FR3      104
           .................<.............><......|..>.........<.|............><........><.....................|..............|..
TRAV1-1P   MFTVIVMCLWLSLDTPWQIHSQTCR*R**CYSVLQIQNK*TERTL*--------WYKQYPKSTPEFLLYI-FQCGAPSPNIPPRMSAEINGNK-----VDLLISSAAVSASALYYCALVS
TRAV1-2    MLTIIFMCLRLSLGDSMADSVEPLSTHKIVDEDDNVILSCRYKTT-SVR-NYLHWYRQYPKSKPEFLLYIVDQSGDLSLNIPPRMSAKVNKNK---E-VDLLISSSAVSDSALYYCALAP
TRAV1-3    MFTVISVCLWLLLGDSMADSIEPLFIHKVLDEGDNVTLSCRYNSSFTGI-NYLHWYRQYPKSTPEFLLYI-SQSGDLSFNIPPRMSAEVNGVK-----VHLLISSAAVSDSALYYCALAP
TRAV1-4    MFTVIFVCLWLLLGDSMADSIEPRFIHKVLDEGDNVTLTCRYNSSFSG--NYLHWYRQYPKSTPEFLLYIVDQSGDLSLNIPPRMSAKVNKNK---E-VDLLISSSAVSDSALYYCALAP
TRAV1-5P   MFTIIFTCLWLSLGDSMADPIEPLVSHNVVDEGDNVTLSCSYKGFTENIYN-VQWYRQYPKSKPDFLLYI-TPSGELRLNIPPRMSANINGENK----WICSFSPLLYRTLHYTTVL*SX
TRAV1-6    MFTVIFVCLWLLLGDSMANSIEALFIHKVVDEDDNVTLSCSYKFSVLGT-NYLHWYRQYPKSTPEFLLYI-SQSGDLSFNIPPRMSVEVNGDK-----VHLLISFAAVSDSALYYCALAP
TRAV1-7    MFKIMFMCLWLSLGDSMADPIEPLVSHNVVDEGDNITLSCSYKGFTENIYN-VQWYRQYPKSKPDFLLYI-TPSGELSLNIPPRMSAKVNGEK----QVDLLIFSTAVSDSALYYCALAP
TRAV1-8    MFTIIFMCLWLSLGDSMADSIEPLFPHKDIGEGDNVTLSCSYKKF-SGTVNYLHWYRQYPKSTPKFLLFI-DPSGFKSNSIPPRLSADIDKNG---KQVDLLISSAAVSDSALYYCALEP
TRAV1-9    MLTIIFMCLWLSLGDSMADSVEPLSTHKVVDEDDNVTLSCRYKTT-SVT-NYLHWYRQYPKSKPEFLLYIVDQRGDLSLNIPPRMSAKVNNNE---E-VDLLISSSAVSDSALYYCALAP
TRAV1-10   MFTVIFTCLCLSLGDSMADSVQPLFTHKVVEEDDDVTLSCRYEITYPTG-NYLHWYRQYPKSTPEFLLYI-SQGGALSSNIPTRMTAKVNGDNK---QLDLLISSAAVSDSALYYCALAP
TRAV1-11P# MFTVIVMCLWLSLG/FMAGPWQIPS--KLVDEGDNVTVSCRYKT-SATER-TL*WYKQYPKSTP*FLLYI-FQCGAPSPNIPPRMSAEVNGNK-----VDLLISSAAVSASALYYCALV
TRAV1-12   MFTVIFLCLWFSLDDSMADPIEPLDTHKAVDEGDNLTLSCSYNKFT-GVVTTLQWYRQYPKSNPEFLLQI-FPSEGKSNTGPPRLSAEV--DQNNK--VDLLISSAAVSDSALYYCALQP
TRAV1-13   MFTVIFICLWLSLGDSMADSIAPIFTQKVVDEGGDVTLSCRYKTNSSVANNYLHWYRQYPKSIPKFLLYI-HQRGTLSANTPPRMSAEVHDDNNNKKQVDLLISSAAVSDSALYYCALQP
TRAV1-14   MFTVIFLCLWLLLGDSMADSIETLFIHKVLDEGDNVTLSCRYNSSY-TRNN-LHWYRQYPKSTPEFLLYI-SQSGDLSSNIPPRMSAEVKGD-----KMHLLISFAAVSDSALYYCALAP
TRAV1-15   MFTVIFTCLWLSLGDSMADSVGPRFTHKVVDEDDDVTLSCSYKTTIRTG-NYLHWYRQYPKSTPEFLLYI-SDGGALSSNIPTRMTAKVNGDN---EQVDLIISSAAVSDSALYYCALAP
TRAV1-16   MFTVIFMCLWLSLGDSMADSIEPLFTHKLVDEGDNVTLSCSYKNSSVSANSNLYWYKQYTKSIPEFLLYI-YPSGIVSPNIPQRMFAKVNG--SIK-QVDLLISFAAVSDSALYYCALRP
TRAV1-17   MFTVIFLCLWFSLGDSMADPIEPLVTHKAVDEGENLTLSCSYNKFIGTVN-MLQWYRQYPKSNPDFLLQI-FPSERKSNTGPPRLSAKV--DQNNK-QVDLLISSAAVSDSALYYCALQP
TRAV1-18   MFTVIFLCLWFSLGDSMADPIEPLDTHKAVDEGDNLTLSCSYKKFTGTVN-MLQWYRQYPKSNPEFLLQI-LPSERKSNTGPPRLSAEV--DQNNKE-VDLLISSAAVSDSALYYCALQP
TRAV1-19   MFTVIFMCLWLSLGDSMADSVEPLFTHTVVDEGDNVTLSCRYNT-SVTTS-YLHWYRQYSKSKPEFLLQI-LSSERKSNTGPPRLSAKV--DQNNK-QVDLLISSAAVSDSALYYCALQP
TRAV1-20   MFTVIFMCLWLSLGDSMANSIEPLVTHKVADEGDNVTLSCNYSG--SINN--LQWYRKYPKYNPEFLLYI-FESGDLSHNIPPQMSAKV---HKDK-QVDLLISSAAVSDSALYYCALVP
TRAV1-21   MFTVIFTCLWLSLGDSMANSVQPRFTHKVVDEDDDVTLSCRYKTTFPTG-NYLHWYRQYPKSTPEFLLYI-TQGGVLSSNIPTRMSAKV--NESNE-QVDLLISSAAVSDSALYYCALVP
TRAV1-22   MFTVIFMCLWFSLGDSMADPIEPLDTHKAVDEGDDVTLSCSYKKLIGTVN-MLQWYRQYPKSKPEFLLQI-LPSERKSNTGPPRLSAE--GDKNNKE-VHLLISSAAVSDSALYYCALEP
TRAV1-23   MSTVIFMCLWLSLGDSLADSVRPLFTHTVVDEGDNVTLSCRYNT-SATTS-YLHWYRQYSKSKPEFLLQI-LSSERKSNTGPPRLSAEV--DRNNK-QVDLLISSAAVSDSALYYCALAP
TRAV1-24   MFTVIFLFLWFSLDDSMADPIEPLDTHKAVDEGDNLTLSCSYKKFTGVVT-TLQWYRQYPKSKPEFLLQI-LPSERKSNTGPPRLSAEV--DQNNKE-VDLLISSAAVSDSALYYCALQP
TRAV1-25   MFNVIFVCLWLLLGDCMADSIEPLFIHKVVDEGDDVTLSCRYNSSASQN-NYLHWYRQYPKSTPEFLLYI-HQSGALSSNIPPRMSAEVNGD--NK-QVDLHISFTAVSDSALYYCALAP
TRAV1-26   MFTVIFMCLWLLSGDSMADPVEPHLTSKVVDEGDNVTLSCSYKNSASVP-DYLYWYKQYPKSKPEYLLFL-TPSGFKSDSIP-RLSAEV-DDNIKR--VDLLISSAAVSDSALYYCALQP
TRAV1-27   MFTVILTCMWLSLGDSMADPIRPLVSHSVVDEGDNVTLSCSYKGFTGSI-YSVQWYRQYPKSKPDFLLYI-TPSGDLSLNIPPRMSANINGEK----QVDLLIFSTAVSDSALYYCALVP
TRAV1-28   MFTVIFTCLWLSLGDSMANSVQPRFTHKVVDEDDDVTLSCRYGTTNPTG-NYLHWYRQYPKSTPEFLLYI-SDGGVLSSNIPTGMTPKVNRD--NKE-VDLLISSAVVSDSALYYCALQP
TRAV1-29   MFTVIFMCLWFSLGDSMADPIEPLDTHKAVDEGDDVTLSCSYKKFIGTVN-MLQWYRQYPKSNPDFLLQI-LPSERKSNTGPPRLSAEV--DQNDKE-VDLLISSAAVSDSALYYCALQP
TRAV1-30   MFTVIFMCLWLSLGDSMADSVEPLFTHTVVDEGDNVTLSCRYNT-SVTTS-YLHWYRQYSKSKPEFLLQI-LPSERKSATGPPRLSAEV--DRNNK-QVDLLISSAAVSDSALYYCALKP
TRAV1-31   MFTVIFLFLWFSLGDSMADPIEPLDTHKAVDEDDNLTLSCSYKKFTGVVT-TLQWYRQYPKSKPEFLLQI-LPSERKSNTGPPRLSAEV--DQNNKE-VDLLISSAAVSDSALYYCALQP
TRAV1-32   MFNVIFVCLWLLLGDCMADSIEPLFIHKVVDEGDDVTLSCKYNSSASQN-NYLHWYRQYPKSTPEFLLYI-HQSGALSSNIPPRMSAEVNGD--NK-QVDLHISFTAVSDSALYYCALAP
TRAV1-33   MFTVILTCMWLSLGDSMADPIRPLVSHNVVDEGDNVTLSCSYKGFTENIYN-VQWYRQYPKSKPDFLLYI-TPSGELSLNIPPRMSAKVNEDK----QVDLLIFSTAVSDSALYYCALKP
TRAV1-34   MFNVIFVCLWLLLGDSMADSIESLFIHKVADEGDNVTVSCTYKFSVSGN-NYLHWYRQYPKSTPDFLLSI-HQSGALSSNIPPRMSAEVKGDK-----VDLLISSAAVSDSALYYCALAP
TRAV1-35P  MFTVIFMCLWLLSGDSMADPVEPHLTSKVVDEGDNVTLSCSYKNSASVQTTCTGTNNIQNPNPNTFFSLL--QVDLKVTPSHICLLKLMIILNEWICSSPLLLYQTLHYTTVLCSX
TRAV1-36   MFTVIFTCMWLSLGDSMADPIRPLVSHNVVDEGDNVTLSCSYKGFTENIYN-VQWYRQYPKSKPDFLLYI-TPSGELSLNIPPRMSAKVNGDK----QVDLLIFSTTVSDSALYYCALVP
TRAV1-37   MFNVIFVCLWLLLGDSMADSIESLFIHKVADEGDNVTVSCTYKFSVSGN-NYLHWYRQYPKSTPEFLLYI-SQSGALSSNIPPRMSAEVKGDK-----VDLLISSAAVSDSALYYCALAP
TRAV1-38   MFTVIFMCLWLLSGDAMADPVEPHLPSKTVDEGDNVTLSCSYKNSASVP-DYLYWYKQYPKSKPEYLLFL-APSGFKSDSIP-RLSAEV-DDNIKR--VDLLISSAAVSDSALYYCALQP
```

```
TRAV1-39    MFTVIFTCMWFSLGDSMADPIRPLVSHNVVDEGDNVTLSCNYKGFTGSIYN-VQWYRQYPKSKPDFLLYI-TPSGELSLNIPPRMSANINGEK----QVDLLIFSTAVSDSALYYCALVP
TRAV1-40    MFNVIFVCLWLLLGDSMADSIEPLFIHNVLDEGDDVTLSCTYKGSALVN-NYLHWYRQYPKSTPEFLLSI-HQSGALSSNIPARMSAKV-DVDK----VHLLISSAAVSDSALYYCALAP
TRAV1-41    MFTVIFMCLWLLSGDSMADPVEPHLTSKVVDEGDNVTLSCSYKNSASVP-DYLYWYKQYPKSKPEYLLFL-TPSGFKSDSIP-RLSAEV-DDNIKR--VDLLISSAAVSDSALYYCALQP
TRAV1-42    MFTVIFMCLWLLSGDSMADPVEPHLTSKVVDEGDNVTLSCSYKNSASVP-DYLYWYKQYPKSKPEYLLFL-TPSGFKSDSIP-RLSAEV-DDNIKR--VDLLISSAAVSDSALYYCALQP
TRAV1-43    MFTVILTCMWLSLGDSMADPIRPLVSHNVVDEGDNVTLSCNYKGFTGSIYN-VQWYRQYPKSKPDFLLYI-TPSGELSLNIPPRMSADINGEK----QVDLLIFSSAVSDSALYYCALKP
TRAV1-44    MFNVIFVCLWLLLGDSMADSIEPLFIHKVVDEGDNVTLSCTYKGSASV-NYYLHWYRQYPKSTPEFLLYI-HQSGALSSNIPARMSAEVKGDK-----VDLLISSAAVSDSALYYCALVP
TRAV1-45    MFTVIFMCLWLSLGDSMADPIEPLLSHNVVDEGDNITLSCSYKGFSGSIYN-VQWYRQYPKSKLDFLFHI-TPSGDLSLNIPPRMSANINGDK----QVDLLIFSPAVSDSALYYCALAP
TRAV1-46    MFTVILMCLWLSLGDSMADSIKALFPHMVVDEGDDVTLSCRYQTSDTAST--LQWYRQYPKSKPEFLLYI-LQSGVQSPNIPPQMSAKVHGDK-----VHLIISSAAVSDSALYYCALKP
TRAV1-47    MFTVIFTCLWLSLGDSMADSVGPRFTHKVVDEDDDVTLSCSYKTTSPTGNY-LHWYRQYPKSTPEFLLYI-SDGGALSSNIPTRMTAKVNRDNK--E-VDLLISSAVVSDSALYYCALVP
TRAV1-48    MFPIIFMCLWLSLGDSMADPIKSLFPHKDQYEGDNVTLSCSY---SGTVRT-LYWYRQYPKSKPEFLLYI-TPSGVKSASIPPRLSAEV-DDD--KKQVDLLISSAAVSDSALYYCALEP
TRAV1-49P#  MFTVIFMCLWLLL/CSMADPIEPIFTHKVVDEIDITLSCSYKDSVALYRISAGIENIQNLNQSSFFTSIQMDLKVTPSHHVCLLKIKLNKWICSSLLLLYQTLHYTTVLCS
TRAV1-50    MLSVIFMCIWLSIGDSLADPIEPLLTHKVVDEGHDVTLSCSYKDFSGTVRT-LQWYKQYPKSTPEFLLYI-TPGGVKSDSVP-RLSAKV-DD---KKQVDLLISSAAVSDSALYYCALEP
TRAV1-51    MFLVILVCLWLSLSDSMTEAIKPLLSHKVVHEDDDVTLSCSYKDFRGTVEN-LHWYRQYPKSKPEFLLYI-YPNGDKSPSIPPRLSAEVDDDDN-KKQVDLLISSAAVSDSALYYCALRP
TRAV1-52    MFPIIFMCLWLSLGDSMADPIKSLFPHKDQYEGDNVTLSCSYSGTV--SI--LYWYRQYPKSKPEFLLYI-TPSGVKSDPIP-RLSAEVDDD---KKQVDLLISSAAVSDSALYYCALQP
TRAV1-53    MFLVILVCLWLSLSDSMTEAIKPLFSHKVVHEDDDVTLSCSYKDFSGRVDN-LQWYRQYPKSKPEFLLYI-TPRGFKSPSIPPRLSAEVDDDDN-KKQVDLLISSAAVSDSALYYCALRP
TRAV1-54    MVSVIFMCLWLSIGDSLADPIEPLLTHKVVDEGDDVTLSCSYRNSASVSI--LYWYKQYPKSTPEFLLYI-TPSGGKSDPIP-RLSAEVDDDDD-KKQVDLLISSAAVSDSALYYCALRP
TRAV1-55    MFLVILVCLWLSLGDSMADPIKPLFSHKVVHEDDDVTLSCSYKDFSGTVDN-LQWYRQYPKSKPEFLLYI-YPSGVKSDPIP-RLSAEVDDDNN-KKQVDLLISSAAVSDSALYYCALQP
TRAV1-56    MFPIIFMCLWLSLGDSMADPIKSLFPHKDQYEGDNVILSCSY---SGTVRT-LYWYRQYPKSKPEFLLYI-TPSGVKSASIPPRLSAEVDDD---KKQVDLLISSAAVSDSALYYCALEP
TRAV1-57    MLSVIFMCIWLSIGDSLADPIEPLLTHKVVDEGDDVTLSCSYKDFSGTVRT-LQWYKQYPKSTPEFLLYI-TPGGVKSDPIP-RLSAKVDNN---KKQVDLLISSAAVSDSALYYCAVEP
TRAV1-58    MFLVILVCLWLSLSDSMADPIKPLLSHKVVHEDDDVTLSCSYKDFSGTVGN-LQWYRQYPKSKPEFLLYI-YPNGDKSPSIPPRLSAEVDDNN--KKQVDLLISSAAVSDSALYYCALQP
TRAV1-59    MLSVIFMCLWLSIGDSLADPIEPLSTHKVVDEGDDVTLSCSYKN-SASVNN-LQWYKQYPKSTPEFLLYI-TPSGVKSDSVP-RLSAKVDDDDD-KKQVDLLISSAAVSDSALYYCALQP
TRAV1-60    MFLVMLVCLWLSLSDSMTEAIKPLFSHKVVHEDDDVTLSCSYKDFSGSVEN-LQWYRQYPKSKPEFLLYI-TPSGVKSPSIPPRLSAKVDNN---KKQVDLLISSAAVSDSALYYCALRP
TRAV1-61    MLSVIFMCLWLSIGDSLADPIEPLLTHKVVDEGDDVTLSCSYKDFSGTVNT-LQWYKQYPKSTPEFLLYI-YPDGDTSDSVP-RLSAEVDDDD--KKQVDLLISSAAVSDSALYYCAVEP
TRAV1-62    MFLVILVCLWLSLSDSMTEAIKPLFSHKVVHEDDDVTLSCSYKDFSGRVDN-LQWYRQYPKSKPEFLLYI-TPSGVKSASIPPRLSAKVDDDNN-KKQVDLLISSAAVSDFALYYCALES
TRAV1-63    MFPIIFMCLWLSLGDSMADPIKSLFPHKDQYEGDNVILSCSY---SGTVRT-LYWYRQYPKSKPEFLLYI-TPSGVKSASIPPRLSAEVDDD---KKQVDLLISSAAVSDSALYYCALEP
TRAV1-64    MLSVIFMCIWLSIGDSLADPIEPLLTHKVVDEGHDVTLSCSYKDFSGTVNT-LQWYKQYPKSTPEFLLYI-TPEGDTSDSVP-RLSAEVDDD---KKQVDLLISSAAVSDSALYYCAVEP
TRAV1-65    MFLVILLCLWLSLSDSMTEAIKPLFSHKVVHEDDDVTLSCSYKDFSGSVNN-LQWYRQYPKSKPEFLLYI-YPSGVKSDPIP-RLSAKVDDDD--KKQVDLLISSAAVSDSALYYCALEP
TRAV1-66    MFPIIFMCLWLSLGDSMADPIKSLFPHKDQYEGDNVILSCSY---SGTVRN-LYWYRQYPKSKPEFLLYI-TPSGVKSASIPLHLSAEVDDD---KKQVDLLISSAAVSDSALYYCALEP
TRAV1-67    MLSVIFMCIWLSIGDSLADPIEPLLTHKVVDEGHDVTLSCSYKDFSGTVNT-LQWYKQYPKSTPEFLLYI-TPSGGRSNSVP-RLSAKVDNN---KKQVDVLISSAAVSDSALYYCAVEP
TRAV1-68    MFLVILVCLWLSLSDSMADPIKPLFSHKVVHEDDDVTLSCSYKDFSGSVGN-LQWYRQYPKSKPEFLLYI-TPSGVKSPSIPPRLSAEVDDDNN-KKQVDLLISSAAVSDSALYYCALRP
TRAV1-69    MLSVIFMCIWLSIGDSLADPIEPLLTHKVVDEGHDVTLSCSYKDFSGTVRT-LYWYKQYPKSTPEFLLYI-TPSGGKSDSVP-RLSTKLDNN---KKQVDLLISSAAVSDSALYYCAVDP
TRAV1-70    MFLVILVCLWLSLSDSMADPIKPLFSHKVVHEDDDVTLSCSYKDFSGTVGN-LQWYRQYPKSKPEFLLYI-TPSGVKSPSIPPRLSAEVDDGNN-KKQVDLLISSAAVSDSALYYCALRP
TRAV1-71    MLSVIFMCIWLSIGDSLADPIEPLLSHKVVDEGHDVTLSCSYKNSASVSI--LYWYKQYPKSTPEFLLYI-TPSGGKSDPIP-RLSAEIDD----KKQVDLLISSAAVSDSALYYCALQP
TRAV1-72    MFLVILVCLWLSLSDSMTEAIKPLLSHKVVHEDDDVTLSCSYKDFSGSVNN-LQWYRQYPKSKPEFLLYI-YPNGVKSDPIP-RLSAKVDDDNN-KKQVDMLISSAAVSDSALYYCALEP
TRAV1-73    MFPIIFMCLWLSLGDSMADPIKSLFPHKDQYEGDNVTLSCSY---SGTVRN-LYWYRQYPKSKPEFLLYI-YPNGDKSASIPPRLSAEVDDD---KKQVDLLISSAAVSDSALYYCALQP
TRAV1-74    MFLLMLVCLWLSLSDSMTEAIKPLFSHKVVHEDDDVTLSCSYKDFSGSVNN-LQWYRQYPKSKPEFLLYI-YPSGVKSPSIPPRLSAKVDDDNN-KKQVDLLISSAAVSDSALYYCALVP
TRAV1-75    MLSVILMCLWLSIGDSLADPIEPLSTHKVVDEGDDVTLSCSYKDFSGTVNT-LQWYKQYPKSTPEFLLYI-YPSGGRSNSVP-RLSAKVDNN---KKQVDVLISSAAVSDSALYHCAVEP
TRAV1-76    MFLVILVCLWLSLSDSMTEAIKPLFSHKVVHEDDDVTLSCSYKDFSGSVNN-LQWYRQYPKSKPEFLLYI-TPRGFKSPSIPPRLSAEVDDDNN-KKQVDLLISSAAVSDSALYYCVLVP
TRAV1-77    MFLVILVCLWLSLSDSMTEAIKPLFSHKVVHEDDDVTLSCSYKDFSGSVNN-LQWYRQYPKSKPEFLLYI-TPSGVKSPSIPPRLSAEVDDDNN-KKQVDLLISSAAVSDSALYYCALVP
TRAV1-78    MFTVLFICLWLLSGDSMADPIKPLLTSKDVDEGDNVTLSCSYKNSASVPDY-LYWYKQYPKSKPEYLLFL-TPSGFKSDSIP-RLSAEV-DDNIKR--VDLLISSAAVSDSALYYCALRP
TRAV1-79    MFLVILVCMWLLLSDSMADPIKPLFSHKVVHEDDDVTLSCSYKDFSGRVDN-LQWYRQYPKSKPEFLLYI-TPSGVKSPSIPPRLSAEVDDDNN-KKQVDLLISSAAVSDSALYYCALRP
```

```
Subgroup 2
TRAV2-1P#   MLLIHSVTPVININ/EAGDEHINPVQTIITSLEGSNTRLICKYDESALSLHWYRQKPQSGPEFLLLIQKSTEFITRDKEPHPGLSIKLHKNESIVVLDLSSASISDSSLYYCAMEP
TRAV2-2     MLIFFSVFIAVTHVAEAGDEHINPVQTIITSLEGNNTRLICKYDESAYSLHWYRQKPQSGPEFLLLIQKSTEFITRAKEPHPGLSIKLHKNESIVVLDLSSASISDSSLYYCAMEP
TRAV2-3     MLIFLSVFIAVTNVAEAGDEHINPVQTIITSAEGSNTRLICKYDESALSLHWYQQKPQSGPEFLLLIQKSTEFIIRNKEPHPGLSIKLHKNESIVVLDLSSASISDSSLYYCAMEP

Subgroup 3 previously Family 3
TRAV3-1     MVRLFLFFFTIADIAEAADNSITPDQTSMSVIEGSNITLSCTYTGSVYSLHWYRQQPGSRPEFLLLIDETSEHVTQAQPPHPQLSTKLDKKNTKVDLLISSVTVTDSALYYCALMP
TRAV3-2     MLFFILFFFTIADIAEAADNSIKPEQTTMSVIEGSNTTLSCTYTGSAYSLHWYRQKPGSRPEFLLLIDKASEHVTQAQPPHPQLSIKLDEKNTKVDLLISSVTLTDSALYYCALRP
TRAV3-3     MLFFILFFFTIADIAEAADNSIKPEQTTMSVIEGSNTTLSCTYTGSAYSLHWYRQKPGSRPEFLLLIDETSEHVTQAQPPHPHLSIKLDKKNTKVDLLISSPTVTDSALYYCALRP
TRAV3-4     MVLLFLFFFTTADIAEAADNSITPEQSSMSVIEGSNITLSCTYTGSVYSLHWYQQKPGSRPGFLLLIAETSEHVTQAQPPHPQLSIKLDKKNTKVDLLISSATVTDSALYYCALRP
TRAV3-5     MVRLFLFFFTIADIAEAAGNSITPEQSTMSVIEGSNTTLSCTYTGSVYSLHWYQQKPGSTPKFLLLITEAIEHVTQAQPPHPHLSIKLDKKNTKVDLLISSAAVTDSALYYCALGP
TRAV3-6     MVLLFLFFFTIADIAEAADNSITPEQSSMSVIEGSNTTLSCTYTGSVDSLHWYQQKPGSRPEFLLLITEASEYVRQAQPPHPQLSIKLDKKNTKVDLLISSAAVTDSALYYCALMP
TRAV3-7     MLLLFLVSFTVAEIAEAADNSINPNQTNVYSTEGSNTKLSCTYTGSAYNLYWYQQKPGSRPEFLLLIYESNEQVTKAQPPHPQLSIKLHKISKKVDLLISSAAVTDSALYYCALRP

Subgroup 4 previously Family 2
TRAV4-1     MFLFFVFICVQNI-GAVDSIITPVQTIISSSEGSNTTLTCKYDQSADYLYWYRQKPQSGPEFLLMIFVS-TDHVTEQSDPRLSTKLRKM-EKGVDLEIFPAAVSDSALYYCALKT
TRAV4-2     MLLFFIFIVVKNIAAVDSSI-TPDQTIISSSEGSITTLTCTYNQSAYSLHWYRQKPQSGPEFLLLIIVSSNDVINAKQPDPRLSIRLRE-GKKVDLEIFPAAVSDSALYYCALEP
TRAV4-3     MLLFFVFIVIKNIAGAVDNNITPDQTIIYSSEGSNTTLSCTYDASAYRLHWYRQKPQSGPEFLLMILVSSNVVTEATQPDPRLSIKLHKKGNKVDLEIVTVAVSDSALYYCALEP
TRAV4-4     MLLFFVFIVVKNIAT-VDS-ITPDQTIISSSEGSITTLTCTYDQSADSLHWYRQKPQSGPEFLLLIIVSSNDVIEAKQPDPRLSIRLRE-GKKADLEIFPAAVSDSAMYYCALQP
TRAV4-5     MLLFFVFIVIKNIAGAVDNSITPDQTIISSSEGSNTTLSCTYDASAYRLHWYRQKPQSGPEFLLMIRVSSDAVTEAKQPDPRLSTKLHKNGNKVDLEIVTVAVSDSALYYCALE
TRAV4-6     MLLFFVFIVIKNIAGAVDNSITPDQTIISSSEGSNTTLSCTYDASAYRLHWYRQKPQSGPEFLLMIRVSSDAVTEAKQPDPRLSTKLHKNGNKVDLEIVTVAISDSALYYCALEP
TRAV4-7     MLLFFIFIVVKNIAT-VDSSITPDQAIISSSEGSITTLTCTYNQSADYIHWYRQKPQSGPEFLLLIIVSSNSVINAKQPDPRLSIRLRE-GKKVDLEIFLAAVSDSALYYCALQP
TRAV4-8     MLLFFIFIVVKNFAT-VDSSITPDQAIISSSEGSITTLTCTYNQSADYIHWYRQKPQSGPEFLLLIIVSSNSVINAKQPDPRLSIRLRK-EKKVDLEIFPAAVSDSALYYCALQP
TRAV4-9     MLLFFIFIVVKNIAT-VDSSITPDQAIIFSSEGSITTLTCTYNQSADYIHWYRQKPQSGPEFLLLIIVSSNSVINAKQPDPRLSIRLRE-GKKVDLEIFPAAVSDSALYYCALEP
TRAV4-10    MLLFFIFIVVKNIAT-VDS-ITPDQTIISSNEGSITTLTCTYNQSADYIHWYRQKPQSGPEFLLLIIVSSNDVINAKQPDPRLSIRLRE-GKKVDLEIFPAAVSDSALYYCALQP
TRAV4-11    MLLFFIFIVVKNIAT-VDSSITPDQAIISSSEGSITTLTCTYNQSADYIHWYRQKPQSGPEFLLLIIVSSNSVINAKQPDPRLSIRLRE-GKKVDLEIFPAAVSDSALYYCALQP
TRAV4-12    MLLFFVFVVVKNIAAAVDSSITPDQTVISSSEGRNTTLTCTYDDSARYLHWYRQKPQSGPEFLLLIIVSSNSVTKANQ--PRLSIRLRE-GKKVDLEIFPAAVSDSALYYCALQP
TRAV4-13    MLLFFVFVIVKNIAAAVDSSITPDQTVISSSEGSNTTLTCTYDDSARYLHWYRQKPQSGPEFLLLIFVSSNSVTKAKQPDPRLSIRLRE-GKKVDLEIFPAAVSDSALYYCALQP

Subgroup 5
TRAV5-1     MFLLGMFILILLLIWSTSMQIIEHLIAEKHVLEGNDAILSCNFTTSAGDYIQWYRQYPKSRPEFHLSIYPSNTGMQSATADKKPEDLKISSAAVSDSAVYYCALKP
TRAV5-2     MFLLGMFILILLLIWSTSMQIIEHLVAEKHVLEGNDAILSCNFTTTAGDYIQWYRQYPKSRPEFHLSIYPSNTGMQSATADKKPEDLKISSAAVSDSAVYYCALKP
TRAV5-3     MFLLGMFILILLLIWSTSMQIIEHLIAEKHVLEGNDVILSCNFTTTAGDYIQWYRQYPKSRPEFHLSIYPSNTGMQSATADKKPEDLKISSAAVSDSAVYYCALKP

Subgroup 6
TRAV6-1    MMVFSTVLFFTMFMGESTQDSITPTSSAVYAKEEQAVTLSCIYEYTVSMNNLQWYRQYSNAAPDFLVLLTESGANQTGDTPHPHLSAKVHKDLKRVDLEISFSALSDSALYYCALQP
TRAV6-2     MVFSTVLFFTMFIGESTQDSITPTSSAVYAKEEQAVTLSCIYEYTVSMNNLQWYRQYSNAAPDFLVLLTESGANQTGDTPHPHLSAKVHKDLKRVDLEISFSALSDSALYYCALQP
TRAV6-3     MLVFSTVLFFTMFIGESTQDSITPTSSAVYAKEEQAVTLSCIYEYTVSMNNLQWYRQYSNAAPDFLVLLTESGANQTGHTPHPHLSAKVHKDLKRVDLEISFSALSDSALYYCALQ
```

The splice sites in the split leader exons result in an unproductive transcript.

Deduced amino acid sequences of catfish TRDV genes

TRDV subgroup 1

```
TRDV1-1P    MAQLYTQH*FSL*QQ------**GSFADKIGLTDEDDNITLKETHCYSEMFI*VKEE*NMALQVQTIS*QRTTVFTHYRSYSSHG---RKATD--SEAKTTADSAELIISDLKLSDSALYHCALTVG
TRDV1-2P    MAQLYTQH*FSL*QQ------**GSFADKIGLTDEDDNITLKETHCYSEMFI*VKEK*HMALQVQTIC*QCTTIFTH*RRKVI*LSWAQGH*HSVRGKNNLL------------*DSALSLRSYSR
TRDV1-2P    other frame             -EVLQIKLG*QMKMTTLL*RKHTVTLKCSYESKRNNIWLYRCKQYANSAPQFLLIKGGRSYSCHGHKATDTLLEAKTTS------------YEILHYHCALTVG
TRDV1-3P    MAQLCNLLYIVRYTTLILTVAT-GSFADKIGLTDEDDNITGKKTHCYSEMLI*IKEK*HVALLVQIICIMLTAHHSFYS*RRKVI*FSW------ARGH*Q------------LSDSAIYHCALTVR
TRDV1-3P    other frame             -EVLQIKLG*QMKMTTLLGRKHTATLKCSYESKRNNMWLYWYK*YALC*QRTTVFTHKGGRSYSSHG-----PEATDS------------SLILQSITVLLQ*E
TRDV1-4     MDQLYNLLYIVRYIPLILTVA-KGSFADKIGPTDEDANIVRHEKDTVTLKCSYESSSQYIYLYWYKQYPNSAPQFLLYKGARSSSSYERKATDTRLEAKTSGDSTELTIRGLKLSDSALYHCALRVG
TRDV1-5P    VAQLYNLLYIVRFSLSY*V****GRFADIIGPKDTSGMIDRKETDTVTLKCSYKSSSNYIRYLENDI-------------------*-RALLMILD*KQKQPEILLYLL*GLNLQILYSFTALLET*
TRDV1-5P    Other frame             QICRYNWAKRHKWDDRQEGNRHCYSEMFI*IKQQLHSISRERYL-------------------E-STPDDP*LEAETTRDFIVLTMR-SKPSDSVLFHCALRNL
TRDV1-6     MSQLHNLLYIVRYTSLILTVAT-GSFANTIGPKDEDVRIVGKDTDTVTLKCSYDTNSDYILLYWYKQYPNSAPQFLLYKGARSRSDE-STPTDTRLESNTNRDSTELTIRGLKLSDSALYHCALRIV
TRDV1-7     MSQLYNLLYIVRYIPLILTV-TKGSFADEIWPTDEDANIVRHEKDTVTLKCSYESSSEYIYLYWYKQYPKSSPQFLLYKGARSYSSHERKATDTRLETKTSGDSTELTIKGLKLSDSALYHCGLRVG
TRDV1-8     MDQLYNLLYIVRYLPLILTV-TKGSFADKIRPTDEDANIVRHEKDTVTLKCSYESSSENIRLYWYKQYPNSAPQFLLYKGARSISFG-STPTDTRLETKTSRNSTELTVRGLKLSDSALYHCALRVG
TRDV1-9P    MAQLCNLQN-EIHTTDSH*------SLVL*IKLVHHHQERIRHCDCDCEMFIFRQ*LHLSLLVQTIS*QHTTVFTI*RCNIKIR*-E-NHSCDLQLETKTTRVFTVLTIRGLKLSDSSLSNCTLRDA
TRDV1-10    MAQLHNLLCIVRYTPLILTVAT-GSFAEKIWPVDEHATIVRKETDTVTLKCSYESRSDYIWLYWYKQYRNSAPQFLLYKGARSESSE-YNPSD-RLQSKTTSESTELTIRGLKLSDSALYHCALRIV
TRDV1-11P   MAHLYNLL---RYIPVIVTVVT-GSFADKIWPTDEYANVVGKETNTVTLKCSYESRSNNILLYWYKQYPNSAPQFLLYKGARSQSGLGSTPTDTRLESKTTRDSTELTI*GLKLSDSLLYHCAVIVV
TRDV1-12    MAQLHNLLYIVRYTSLIFTVAT-GSFANTIGPKDEDVRIVGKETDTVTLKCSYKTNSDDIYLYWYKQYPNSTPQFLLYKGARSRSDE-STPTDTRLASNTARESTELTIRGLKLSDSALYHCALRVV
TRDV1-13    MARLYNLLYIVRYILLILTIA-ADSVADKIGPKDEDGNIIKKETDTVTLKCSYESSSEYIYLYWYKQYPNSAPQYLLYKSARSGSGQ-STPDDTRLEAKTRGNSTELTVRGLKLSDSALYHCALRVG
TRDV1-14    MSQLYNLLYIVRYIPLILTV-TKGSFADKIWPTDEDANIVRHEEDTVTLKCSYESSSENIWLYWYKQYPNSAPQFLLYKGARSYSSG-STPTDTRLETKTSRNSTELTIRGLKLSDSALYHCALRVG
TRDV1-15    MAQLHNLLYIARYTSLILTVAT-GSFANSIGPKDEDANIVGKEADTVTLKCSYETSSQNIWLYWYKQYPNSAPQFLLYKGARSSSAA-STPTDTRLESNTNRDSTELTIRGLKLSDSALYHCALEV
TRDV1-16    MAQLYNLLYIARYIPLILTVAT-GSFADNIGPLDEDANIVGKETDTVTLKCSYVAGSNFIWLYWYKQNPNSPLQFLLYKGARSSSGSERTPDDRRLEAQTTTDSTTLTIRGLKLSDSALYHCALRDV
TRDV1-17    MSQLYNLLYIVRYIPLILTV-TKGSFADKIWPTDEDANIVRHEEDTVTLKCSYETSSKYIYLYWYKQYPNSAPQFLLYKGAKSYSSG-STPTDTRLETKTSGDSTELTIRSLKLSDSALYHCALRVG
TRDV1-18    MAQLYNLLYIVRYIPLILTVTT-GSFADNIGPLDEDANIVGKETDTVTLKCAYVAGSNFILLYWYKQNPNSPLQFLLYKGARSNSGSERTPDDRRLEAQTTTDSTTLTIRGLKLSDSALYHCALRDV
TRDV1-19    MSQLYNLLYIVRYIPLILTV-TKGSFADKIWPTDEDANIVRHEEDTVTLKCSYETSSKYIYLYWYKQYPNSAPQFLLYKGAKSYSFG-NTPTDTRLETKTSRNSTELTIRGLKLSDSALYHCALRVG
TRDV1-20    MAQLHNLLYIARYASLILTVAT-GSFANTIGPKDDVFSIVGKETDTVTMKCSYKTSSDYIYLYWYKQYPNSAPQFLLYKGARSSSAA-STPTDTRLESNTNRDSTELTIRGLKLSDSALYHCALEV
TRDV1-21    MSQLYNLLYIVRYIPLILTV-TKGSFADKIWPTDEDANIVRHEEDTVTLKCSYETSSENIRLYWYKQYPNSAPQFLLYKGARSYSSG-STPTDTRLETKTSRNSTELTIRGLKLSDSALYHCALRVG
TRDV1-22    MAQLHNLLYIARYASLILTVAT-GSFANTIGPKDDVFSIVGKDTDTVTLKCSYKTSSEYIYLYWYKQYPNSVPQFLLYKGARSSSAA-STPTDTRLESNTNRDSTELTIRSLKLSDSALYHCALEV
TRDV1-23    MAQLYNLLYIVRYIPLILTVAT-GSFADNIGPLDVDANIVRKETDTATLKCSYVAGSNYILLYWYKQNPNSPLQFLLYKGARSSSDWEHTPDDRRLEAQITTDSTTLTIRGLKLSDSALYHCALRDV
TRDV1-24    MAQLYNLLYIVRYIPLILTV-TKGSFADKIWPTDEDANIVRHEEDTVNLKCSYETSSENIRLYWYKQYPNSAPQFLLYKGARSYSSG-STPTDTRLETKTSRDSTELTIRGLKLSDSALYHCALRVG
TRDV1-25    MAQLYNLLYIVRYIPLILTVAT-GSFADNIGPLDEDANIVRKETDTVTLKCSYVAGSNYILLYWYKQNPNSPLQFLLYKGARSRSDLERTPDDRRLEAQTTTDSTTLTIRGLKLSDSALYHCALRDV
TRDV1-26    MAQLHNLLYIARYTSLILTVAT-GSFADTIGPKDEDVSIVGKETDTVTLKCSYESSSNYIRLYWYKQYPNSAPQFLLYKGARSRSAE-STPTDTRLYSNTARDSTELTIRGLKLSDSALYHCALRIV
TRDV1-27    MAQLYTLLCIVRYILLILTVATAGGFAAKIWLMDKDANIVSKGADTVTLKCSYETSSEDILLYWYKQYPNSAPQFLLYKGARSRTAQ-STPDDRRFEAKTTGNSTELTIRGLKLSDSALYFCALRDL
TRDV1-28    MAQLYTLLCIVRYTLLILTVATAGGFAAKIWLMDKDANIVSKGADTVTLKCSYETSSEDILLYWYKQYPNSAPQFLLYKGARSRTAQ-STPDDRRFEAKTTGNSTELTIRGLKLSDSALYFCALRDL
```

TRDV subgroup 2

```
TRDV2-1     MDQLYNLLYIVRYIPLILTVAT-GSFANKIWPTDKNAILVGKETDTVTLKCSYESSSS-VWLYWYKQHPNSAPQFLVYVG--------GNPTDTRFQATSSSDSTELTIRGLKLSDSALYHCALRVV
TRDV2-2     MDQLHNLLYIVRYIPLILTVAT-GSFANKIWPTDKNAILVGKETDTVTLKCSYESSSS-VLPCWYKQHPNSAPQFLVYVG--------GNPTDTRFQATSSSGSTELTIRGLKLSDSALYHCALRVG
TRDV2-3     MDQLHNLLYIMRYIPLILTVAT-AVLQIKSGQRKKNAILVGKETDTVTLKCSYESSSS-VLPCWYKQHPNSAPQFLVYVG--------GNPTDTRFQATSSRGSTGLTIRGLKLSDSALYHCALRDV
TRDV2-4     MDQLHNLLYIVRYIPLILTVGS-GSFANKIWPTDKNAILVGKETDTVTLKCSYESSSS-VWLYWYKQHPNSAPQFLVYVG--------GNPTDTRFQATSSSDSTELTIRGLKLSDSALYHCALRVV
```

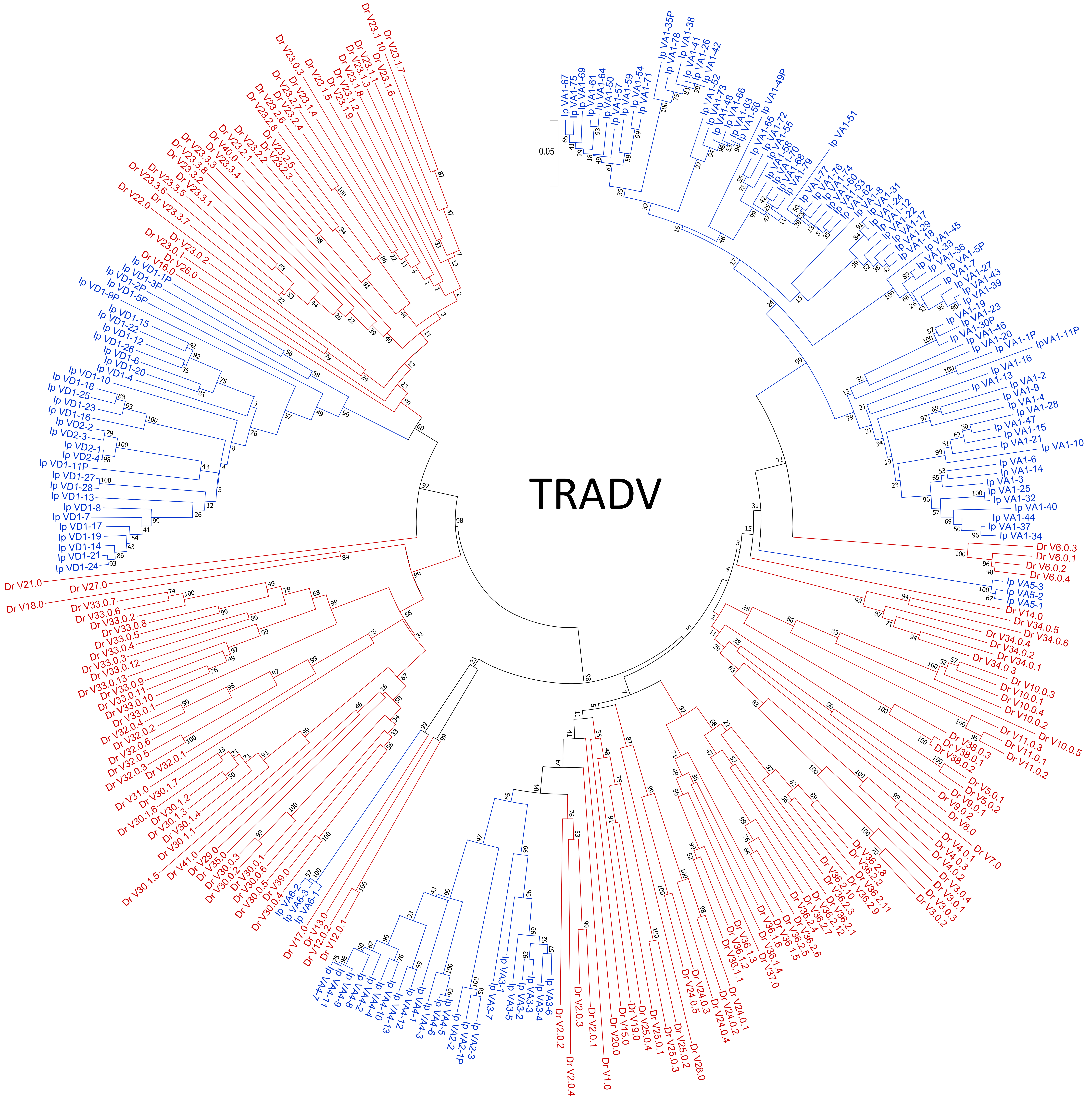
TRADV
0.05
Ip VA1-67
Ip VA1-75
Ip VA1-69
Ip VA1-61
Ip VA1-64
Ip VA1-50
Ip VA1-57
Ip VA1-59
Ip VA1-54
Ip VA1-71
Ip VA1-35P
Ip VA1-78
Ip VA1-38
Ip VA1-41
Ip VA1-26
Ip VA1-42
Ip VA1-52
Ip VA1-73
Ip VA1-48
Ip VA1-66
Ip VA1-63
Ip VA1-56
Ip VA1-49P
Ip VA1-65
Ip VA1-72
Ip VA1-55
Ip VA1-51
Ip VA1-58
Ip VA1-70
Ip VA1-68
Ip VA1-79
Ip VA1-77
Ip VA1-76
Ip VA1-74
Ip VA1-60
Ip VA1-53
Ip VA1-62
Ip VA1-8
Ip VA1-31
Ip VA1-24
Ip VA1-12
Ip VA1-22
Ip VA1-17
Ip VA1-29
Ip VA1-18
Ip VA1-45
Ip VA1-33
Ip VA1-36
Ip VA1-5P
Ip VA1-7
Ip VA1-27
Ip VA1-43
Ip VA1-39
Ip VA1-19
Ip VA1-23
Ip VA1-30P
Ip VA1-46
Ip VA1-20
Ip VA1-1P
IpVA1-11P
Ip VA1-16
Ip VA1-13
Ip VA1-2
Ip VA1-9
Ip VA1-4
Ip VA1-28
Ip VA1-47
Ip VA1-15
Ip VA1-21
Ip VA1-10
Ip VA1-6
Ip VA1-14
Ip VA1-3
Ip VA1-25
Ip VA1-32
Ip VA1-40
Ip VA1-44
Ip VA1-37
Ip VA1-34
Dr V6.0.3
Dr V6.0.1
Dr V6.0.2
Dr V6.0.4
Ip VA5-3
Ip VA5-2
Ip VA5-1
Dr V14.0
Dr V34.0.5
Dr V34.0.6
Dr V34.0.4
Dr V34.0.2
Dr V34.0.1
Dr V34.0.3
Dr V10.0.3
Dr V10.0.1
Dr V10.0.4
Dr V10.0.2
Dr V10.0.5
Dr V11.0.3
Dr V11.0.1
Dr V11.0.2
Dr V38.0.3
Dr V38.0.1
Dr V38.0.2
Dr V5.0.1
Dr V5.0.2
Dr V9.0.1
Dr V9.0.2
Dr V8.0
Dr V7.0
Dr V4.0.1
Dr V4.0.3
Dr V4.0.2
Dr V3.0.4
Dr V3.0.1
Dr V3.0.3
Dr V3.0.2
Dr V36.2.8
Dr V36.2.11
Dr V36.2.9
Dr V36.2.1
Dr V36.2.12
Dr V36.2.3
Dr V36.2.10
Dr V36.2.2
Dr V36.2.7
Dr V36.2.4
Dr V36.2.6
Dr V36.2.5
Dr V36.1.5
Dr V36.1.4
Dr V36.1.6
Dr V37.0
Dr V36.1.3
Dr V36.1.2
Dr V36.1.1
Dr V24.0.1
Dr V24.0.2
Dr V24.0.4
Dr V24.0.3
Dr V24.0.5
Dr V28.0
Dr V25.0.2
Dr V25.0.3
Dr V25.0.1
Dr V25.0.4
Dr V15.0
Dr V19.0
Dr V20.0
Dr V1.0
Dr V2.0.1
Dr V2.0.3
Dr V2.0.2
Dr V2.0.4
Ip VA3-6
Ip VA3-4
Ip VA3-3
Ip VA3-2
Ip VA3-5
Ip VA3-1
Ip VA3-7
Ip VA2-3
Ip VA2-1P
Ip VA2-2
Ip VA4-5
Ip VA4-6
Ip VA4-3
Ip VA4-1
Ip VA4-12
Ip VA4-13
Ip VA4-10
Ip VA4-4
Ip VA4-2
Ip VA4-8
Ip VA4-9
Ip VA4-11
Ip VA4-7
Ip VA6-1
Ip VA6-3
Ip VA6-2
Dr V12.0.1
Dr V12.0.2
Dr V13.0
Dr V17.0
Dr V30.0.4
Dr V39.0
Dr V30.0.5
Dr V30.0.6
Dr V30.0.1
Dr V30.0.2
Dr V30.0.3
Dr V35.0
Dr V29.0
Dr V41.0
Dr V30.1.5
Dr V30.1.1
Dr V30.1.4
Dr V30.1.3
Dr V30.1.2
Dr V30.1.6
Dr V30.1.7
Dr V31.0
Dr V32.0.1
Dr V32.0.3
Dr V32.0.5
Dr V32.0.6
Dr V32.0.2
Dr V32.0.4
Dr V33.0.1
Dr V33.0.10
Dr V33.0.11
Dr V33.0.9
Dr V33.0.13
Dr V33.0.12
Dr V33.0.3
Dr V33.0.4
Dr V33.0.5
Dr V33.0.8
Dr V33.0.2
Dr V33.0.6
Dr V33.0.7
Dr V18.0
Dr V27.0
Dr V21.0
Ip VD1-24
Ip VD1-21
Ip VD1-14
Ip VD1-19
Ip VD1-17
Ip VD1-7
Ip VD1-8
Ip VD1-13
Ip VD1-28
Ip VD1-27
Ip VD1-11P
Ip VD2-4
Ip VD2-1
Ip VD2-3
Ip VD2-2
Ip VD1-16
Ip VD1-23
Ip VD1-25
Ip VD1-18
Ip VD1-10
Ip VD1-4
Ip VD1-20
Ip VD1-6
Ip VD1-26
Ip VD1-12
Ip VD1-22
Ip VD1-15
Ip VD1-9P
Ip VD1-5P
Ip VD1-2P
Ip VD1-3P
Ip VD1-1P
Dr V16.0
Dr V26.0
Dr V23.0.1
Dr V23.0.2
Dr V23.3.7
Dr V22.0
Dr V23.3.6
Dr V23.3.5
Dr V23.3.1
Dr V23.3.2
Dr V23.3.8
Dr V23.3.3
Dr V40.0
Dr V23.3.4
Dr V23.2.1
Dr V23.2.2
Dr V23.2.5
Dr V23.2.3
Dr V23.2.8
Dr V23.2.6
Dr V23.2.7
Dr V23.2.4
Dr V23.1.4
Dr V23.0.3
Dr V23.1.5
Dr V23.1.9
Dr V23.1.2
Dr V23.1.8
Dr V23.1.3
Dr V23.1.1
Dr V23.1.10
Dr V23.1.6
Dr V23.1.7

Catfish TRAD locus

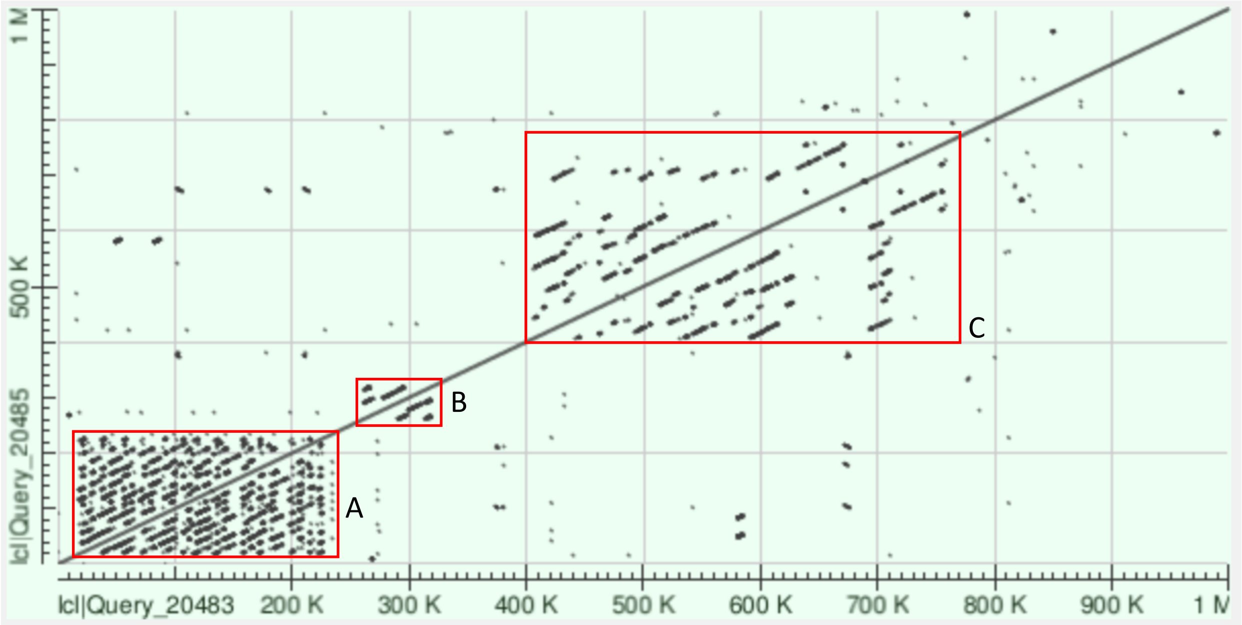

Dot plot analysis of the catfish TRAD locus from nucleotide 446,400-1,446,399. The dot plot was created using the blastn suite 2-sequences (1) using the default parameter except that the word length was set at 128. Tandem duplications are visible as diagonal lines.

A. The rectangle depicts the location of multiple 5-12 kb duplications within the 220 kb region that encodes TRAV1-50 to TRAV1-77 of the TRAV1 subgroup.

B. The rectangle depicts the location duplicated regions that contain two sets of tandem duplications. The first set involves subgroups TRAV6 and TRAV5 and resulted in TRAV6-3 and TRAV5-3; TRAV6-2 and TRAV5-2; TRAV6-1 and TRAV5-1.
The second set of duplications involves members of subgroups TRDV1 and TRAV4 and resulted in TRDV1-28 and TRAV4-13; TRDV1-27 and TRAV4-12.

C. The third region of duplications contains four copies of an array consisting of TRDV2-TRAV1-TRAV1 -TRAV4 interspersed among members of the TRDV1 and TRAV1 subgroups.

TRDV2-4 – TRAV1-44 – TRAV1-43 – TRAV4-11
TRDV2-3 – TRAV1-40 – TRAV1-39 – TRAV4-10
TRDV2-2 – TRAV1-37 – TRAV1-36 – TRAV4-9
TRDV2-1 – TRAV1-34 – TRAV1-33 – TRAV4-8

Catfish TRB locus

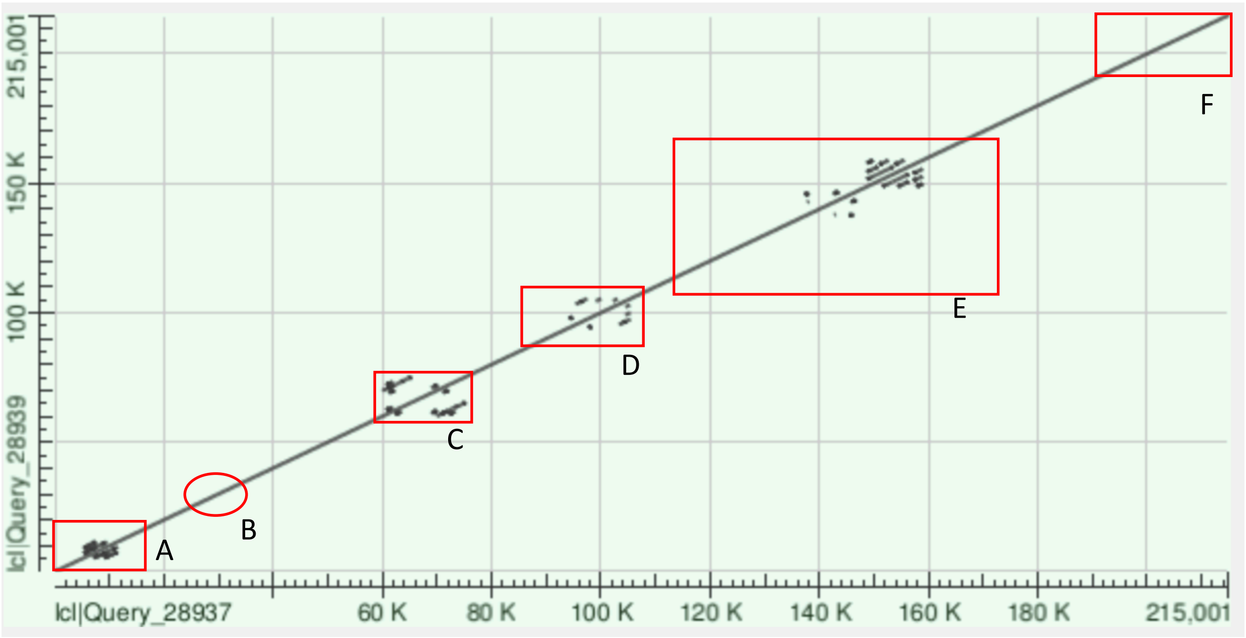

Dot plot analysis of the catfish TRB locus from nucleotide 610,000-825,000. The dot plot was created using the blastn suite 2-sequences (1) using the default parameter except that the word length was set at 128. Tandem duplications are visible as diagonal lines.

A. The rectangle depicts the location of duplications in the region, which encodes members of the TRBV30 subgroup.
B. As a reference the oval depicts the location of the TRBJ genes.
C. The rectangle depicts the location of duplications within subgroup TRBV27.
D. The rectangle depicts the location of duplications within subgroup TRBV19.
E. The rectangle depicts the location of the TRBV7 subgroup and its 15 members which are interspersed by V segments from subgroups 11, 12, 14 and 15.
F. As a reference the rectangle depicts the location of V segments from subgroups 1, 2, and 3.

Reference

1. Zheng Zhang, Scott Schwartz, Lukas Wagner, and Webb Miller (2000), "A greedy algorithm for aligning DNA sequences", J Comput Biol 2000; 7(1-2):203-14.

Deduced amino acids of catfish TRAJ genes are grouped based on >75% nucleotide identity.

The largest TRAJ subgroup consists of 12 members

TRAJ47	TQSGNYKIYFGSGIKLIVEK
TRAJ58	TGSGNLKLYFGSGIKLTVEN
TRAJ62	TDSGGWKLYFGSGIKLTVEK
TRAJ66	TDSGGRKLYFGSGIKLMVEK
TRAJ68	NDGGFKLYFGSGIKLIVQK
TRAJ73	TDGGGFKLYFGSGIKLTVEK
TRAJ76	TDTGTKKIYFGSGIKLTVET
TRAJ80	TEGFGKIYFGSGIKLTVET
TRAJ81	TNGVQKIYFGSGIKLIVET
TRAJ82	TDGVGRIYFGSGIKLIVET
TRAJ85	TTIVQRIYFGSGIKLILET
TRAJ86	TDGVRRIYFGSGIKLIVET

TRAJ 7-member group

TRAJ24	NSGSGYQTLIFGSGTKLIINS
TRAJ39	NTGSGYQKLIFGSGTKLIINC
TRAJ55	NSRSGNQKLIFGSGTKVIINS
TRAJ30	NAGGNQKLIFGSGIKLVINS
TRAJ35	NTGGNQKLILGSGIELIINS
TRAJ61	NAGTGYGKLIFGSGIKLIINS
TRAJ65	NTGGGYGKLIFGTGIALTINS

TRAJ 6-member group

TRAJ37	SNTYGEKLTFGKGIKLLLQS
TRAJ50	SNSYGGKLTFGKGIKLLVQS
TRAJ48	TNNFGGKLTFGKGIKVLVQS
TRAJ60	RSLGQKLIFGKGTKVTVIS
TRAJ64	VRSSGQKVIFGKGTKLTVIS
TRAJ75	TNSGQKIIFGKGTKLIVIS

Two TRAJ subgroups have 5 members

TRAJ93	NVAGQKVIFGKGTVLHIES
TRAJ94	NDGRQRIIFGKGTALHIES
TRAJ95	NDAGRRIIFGKGTVLHIES
TRAJ96	TDGGIRIIFGKGTVLHIES
TRAJ99	NDGARKIIFGKGTVLHIES

TRAJ97	TFGDRKIIFGTGIKLTVTA
TRAJ104	TPGNTKIIFGTGIKLTVTA
TRAJ111	TSGYQKIIFGTGTKLTVTA
TRAJ113	TSQNKKIIFGTGTKLTVTA
TRAJ117	TSGSQKIIFGTGTKLTVTA

Four TRAJ subgroups have 3 members

TRAJ16	DANRDKLIFGHGTKLTITS
TRAJ18	DAGGRNKLIFGHGTKLSITS
TRAJ20	YAGAGNKLIFASGTKLIITS

TRAJ22	YAGNKLIFGAGTQLIVDS
TRAJ29	DAVNKLIFGAGIQLIVES
TRAJ38	NDGNKLIFGAGTQLIVES

TRAJ100	TTGGAAAKLFFATGIKLIIES
TRAJ103	NTGVGVGKLLFATGMKLIIES
TRAJ115	TSGAAVGKLLFATGIKLIIES

TRAJ102	TFENKLIFGQGIMLTINY
TRAJ112	TSGYKLIFGQGIKLTVND
TRAJ114	TFGNKLIFGQGIKLTVNY

Tweleve TRAJ subgroups consists of pairs (2 members)

TRAJ3	TVGEYKIIFGRGTWLYVAA
TRAJ10	TAGEYKLIFGKGTWLYVAA

TRAJ9	TREGNKIYFGSGIKLSVES
TRAJ13	TRGDNKIIFGSGIKLSVEL

TRAJ17	ARSGDKIIFGKGTKLLVEV
TRAJ19	TRGADKIMFGKGTKLLVEV

TRAJ25	TASGGQYKLIFGEGMTLTVNS
TRAJ56	TMSGGAYKIIFGEGTTLTVNS

TRAJ26	TNTGGNRIYFGSGMKLTVEQ
TRAJ41	TNTGNNRIHFGSGMKLIVEK

TRAJ34	DNNLGKVIFGNGIKLLVQS
TRAJ54	DNNARKVIFGKGIKLLVQS

TRAJ70	INTGDRRIYFGSGIKLNTES
TRAJ78	TQTGDWRIYFGSGIKLITEP

TRAJ71	NNNRIYFGSGIKLITES
TRAJ77	NNYRIYFGSGIKLITES

TRAJ72	NAGGGYKIIFGTGIVLTVQT
TRAJ84	TAGAGYKIIFGRGIVLTVKT

TRAJ101	TTVGYDKIIFGRATKLIVQS
TRAJ110	TTVGFEKIIFGRATQLMVLS

TRAJ106	TDGVRIIFGKGTLLTVQL
TRAJ119	TDGYRIILGKGTWLTVQL

TRAJ120	NDGYGKIIFASGTKLNIHT
TRAJ122	NPSYGKIIFASGTKLIIPT

Fiftyfour TRAJ genes are SINGLE member subgroups

TRAJ1	TSDSRYKISFGAGTKLFVQS	TRAJ52	TGDAGGYKLIFGEAMKFTVIS
TRAJ2	TTQGTTKIIFGTGANLIVES	TRAJ53	YGNNRIYFGSGTNLIVEK
TRAJ4	TSGTNGKLIFGHGTRLFIGK	TRAJ57	TRDNRIYFGSGTKLVVEK
TRAJ5	SADNRRLIFGHGTKLLIHA	TRAJ59	NAQTGVDKIIFGTGTKLITIT
TRAJ6	TGGANKVIFGTSIKLTVDT	TRAJ63	TTGTGAYKLIFGTATKLTIET
TRAJ7	TNNRDKIIFGKGTQLFVQS	TRAJ67	TTGSGINKIIFGKGTKLIIET
TRAJ8P	NTIGVNKIIFGKGTSLIVKSF	TRAJ69P	TKTLY*TLRIYFVSGTKFIIET
TRAJ11	TYSGNGKLIFGHGIRLLIEK	TRAJ74	IAQGGVYKLIFGTGTKLIVET
TRAJ12	NTGGVNKIIFGQGISLIVES	TRAJ79	SDALQRIYFSSGIKLIVET
TRAJ14	TAGTEKIIFGKGTKLHVEA	TRAJ83	TGSDNRIYFGSGIKLVTES
TRAJ15	TLSNDKIIFGKGTRLHVEV	TRAJ87	TKSDWKIYFGSGIQLITET
TRAJ21	NPGGTRIIFGAGTQLIVEE	TRAJ88	TTGYKMIFGTGIVLTVQS
TRAJ23	KNNVKLTFGTGIKLLVQS	TRAJ89	TSSGNWKIYFGSATKLIVET
TRAJ27	TNNFGKMIFGEGIKLLIQS	TRAJ90	TYTGGQKLIFGKGTKLRVIS
TRAJ28	NLENTRIFFGAGIQLIVEK	TRAJ91	TTGGERKLFFGKGTKLVIET
TRAJ31	TTSAGGYKLIIGEGTKLTVNS	TRAJ92	TDGFKLIFGKGTKLIVNS
TRAJ32	TYSGANQIYFGSGTKLIIEK	TRAJ98	TTGGSFRKIFLATGIKLTVES
TRAJ33	ANTGGRIYFGSGTKLIVEK	TRAJ105	NYANTIIFGKGTTLIVQS
TRAJ36	TTTGSYRIYFGSGTKLIVDK	TRAJ107	TAGGALSKIIFATGIELRIES
TRAJ40	VTRGAEQYKLIFGEGTTLTVNS	TRAJ108	TAGTKIIFGQGIKLTLIVV
TRAJ42	NTNVDKLIFGNGIKLLVQS	TRAJ109P	VTTGGGVGKLFFVTGIK*KI
TRAJ43	NLGNARIIFGAGTRLIVEP	TRAJ116	TTGGYNVIFGKVIKLIVMA
TRAJ44	SNNFAKLTFGNGIKLLVES	TRAJ118	NYANDKLIFGNGTSLIVQS
TRAJ45	TGGASQYKVIFAEGVKLTVNS	TRAJ121	TSGSWKVIFGTGNKFTVTS
TRAJ46	TYSGGDKIYFGSGTQLIVER	TRAJ123	NTGNKIIFGHGTEVIIHT
TRAJ49	VNVNKVIFGAGTQLIVQS	TRAJ124	NDYSRIIFGSGIKVTVEK
TRAJ51	NTLGGYQKLIFGSGFYCYAFH	TRAJ125P	DTNLRVIFGHGT*LIIQL

Deduced amino acid sequences of catfish TRGV genes

Subgroup 1 previously subgroup 3

TRGV1 MTMSNMTAACFLLVLIGCISAQFSHPGPWEIINRDRSKRMSCTVDSSVTLSSTALHWYRAKPGKALQRILYFAAGASTGTSENECLSRCNGGRKGNIFTLTITKVNDDDAATYYCALWRG

Subgroup 2 previously subgroup 1

TRGV2-1 MFIAIYAVLFSLVTEAVLGLTLEQKDLSMTKEEGKTVYISCKVTGLSSSSYVHWYQKKEGEALARILYIKSGSLEPVHDANHNEAKEFGVRKQDDNYDLKIANLKKSHSAVYYCACWES
TRGV2-2 MFIAIYAVLFSLLTEAVLGVTLEQKDLSMTKGEGKSVYISCKVTGL-TTTYVHWYQQKDGEALTRILYVSSGS-KPVHDTTHPEAKDFDVRLQSDNYDLKIANLKKSHSAVYYCASWETGY
TRGV2-3 MFIAIYAVLFSLVTEAVLGLTLEQKDLSMTKEEGKSVYISCKVTGLSSSNYVHWYQKKEGEALTRILYVKSGSLEPVHDANHNEAKEFGVRTQSDNFDLKIANLKKSHAAVYYCASWDS
TRGV2-4 MFIAIYAVLFSLLTEAVLGVTLEQKDLSMTKGEGKSVYISCKVTGL-TTTYVHWYQQKDGEALTRILYVSSGS-KPVHDTNHLEAKDFDVRLQSDNYDLKIANLKKSHSAVYYCVCWTGVSSY
TRGV2-5 MFIAIYAVLFSLVTEAVLGLTLEQKDLSMTKEEGKSVYISCKVTGLSLSNYVHWYQKKEGEALTRILYVKSGSLEPVHDANHNEAKEFGVRTQSDNFDLKIANLKKSHAAVYYCASWDS
TRGV2-6 MFIAIYAVLFSLLTEAVLGVTLEQKDLSMTKEEGKSVYISCKVTGL-TTIYVHWYQQKDGEALTRILYVKRGS-KPVHDTTHLEAKEFDVRLQADNFDLKIANLKKSHSAVYYCVCWESA
TRGV2-7P MFIAIYAVLFSLVKEAVLGVTLEQKDLSM---QSYANHHEALHHEGLQCEESVKSDNYELKIACLKKIHLAGYYCTRC
TRGV2-8 MSISIYALLFSLVTEPVLGVTLEQKDLSVTKDEGKTVPINCKVTGLSSGSYVHWYQKKEGEALTRILYVKRDS--SVIRDNHDDAKDFDVRIQYDNYDLKIASLKKSHSAVYYCASWESA
TRGV2-9P# MFIAINAVLFLLVT/CCINLSGSETQTKRSLHDKRSGQNCCKVNDLSTTYLHWYQKKDDKALTRILHVKKDSP--PVQDTNYPEAKDFDVRENSGNYDVMISKLKKSHSAVYYCASWESSY
TRGV2-10 MSIGIYTVIFSLLTEAVLGVTVEQKDLSMTKGEGKSVYISCKVTEL-TTDYVHWYQKKDGEALTRILYVKKNS--PVIRDNHLEVNDFDVRTQSDNFDLKIAKLKKSHSAVYYCASWKRD

Subgroup 3 previously subgroup 2

TRGV3-1 MHLSLQLCLLMIFLIYPVHSDVELRQKFLMTKPTTKLAKIECTFPSECRFYIHWYQKKNGEPFKRVQYVDIDDQTTRNDPGFETLKSEKIGN-KFVLIIPNLKQEHSATYYCACWVW
TRGV3-2 MQLSFQLFL---FLLCAVDSDVQLHQKMLMTKPATKLAKIECTFPSDCYFYIHWYQTKESETFKMVQYVKINDGTNHNEPGFEFLKSERTANDKFALIIPNLRTEHSATYYCVCWMWELRTV
TRGV3-3 MHLILHFLLL-IFLICSVDNAVELHQKFLMTKPANKLTEIECTFPTDCNYYIHWYQKKNGEPFKRVQYVGIINAYQSNDRGFEFLQSEKIASNKVVLIIPNVKPEHSATYYCACWVDY
TRGV3-4 MHLILHFLLL-IFLICSVDNAVELHQKFLMTKPANKLTEIECTFPSNCRFYIHWYQKKNGEPFKRVQYVDIDDQSICNEPGFETLKSEKRGN-KFVLIIPNLKREHSATYYCACWVDY

Catfish TRGJ genes

TRGJ1 **ACTTTTTGT**TCTTGCCTGTCA**CATTGTG**---------------------------GGAATGAGAAAGTCTTCAGCTCCGGCATCAGACTGTATGTTACAG/GT
N E K V F S S G I R L Y V T

TRGJ2 **TGTTTTTAT**GACTGAATGTCA**CACTGTG**----------------------------TGCAGTGAAAGTCTTCGGCTCAGGGACAAGACTTTACGTAACAG/GT
A V K V F G S G T R L Y V T

TRGJ3-1 **TAATTTTTT**GTCATTGGACAT**CATGCTG**---------------------TGGGATACTGGGTGAAGGTTTTCGGCTCAGGAACAAGACTTTACGTAACAG/GT
G Y W V K V F G S G T R L Y V T

TRGJ3-2 **TATTTGATG**TAGACTGTGTCA**CAGTGTG**------------TTGCTGGATACACCTACTACTACCAAGTATTTGGATCTGGAACTCGACTTATCGTCACTG/GT
A G Y T Y Y Y Q V F G S G T R L I V T

TRGJ3-3 **TATTTGGTG**TAGTCTGAGTCG**CAGTGTG**-------------------AACTGGATACAGCTACAAAGTATTTGGATCTGGAACTCGACTTTTCGTCACTG/GT
T G Y S Y K V F G S G T R L F V T

TRGJ3-4P **TGTTTCTGA**GTTTGACACTCA**CAACTAG**-------GGCAACATATATCTTTATAAAGTATCTGGAAGTATTTGGATCTGGAACTCGGCTTATCATCACTG/GT
A T Y I F I K Y L E V F G S G T R L I I T

TRGJ3-5 **TATTTGATG**TAGACTGTGTTA**CAGTGTG**---------TCACTGGAAGCACTTACTACTCCTACAAAGTATTTGGATCTGGAACTCGGCTTATCGTCACTG/GT
T G S T Y Y S Y K V F G S G T R L I V T

TRGJ3-6 **TATTTAGTG**TAGCCTGAGTGA**CAGTGTG**-------------------AACTGGAGACGACTACAAAGTGTTTGGATCTGGAACTAGACTTATCGTCACTG/GT
T G D D Y K V F G S G T R L I V T

TRGJ4-1P **GAAGTCATA**TGGTCTATTAGA**CATCATG**------------------TTATGCAAAGCTGGAGGAAGGTTTTTGTCTCAATAACAAGACTTTACATATCAG/GT
M Q S W R K V F V S I T R L Y I S

TRGJ4-2 **TATTTGATG**TAGTCTGTGTCA**CAGTGTT**------ACTTCAATGGCTACTACTACTACTCCAAGAAAGTATTTGGATCTGGAACTCGGCTTATCGTCACTG/GT
F N G Y Y Y Y S K K V F G S G T R L I V T

TRG region between TRGV2-8 and TRGV2-9P

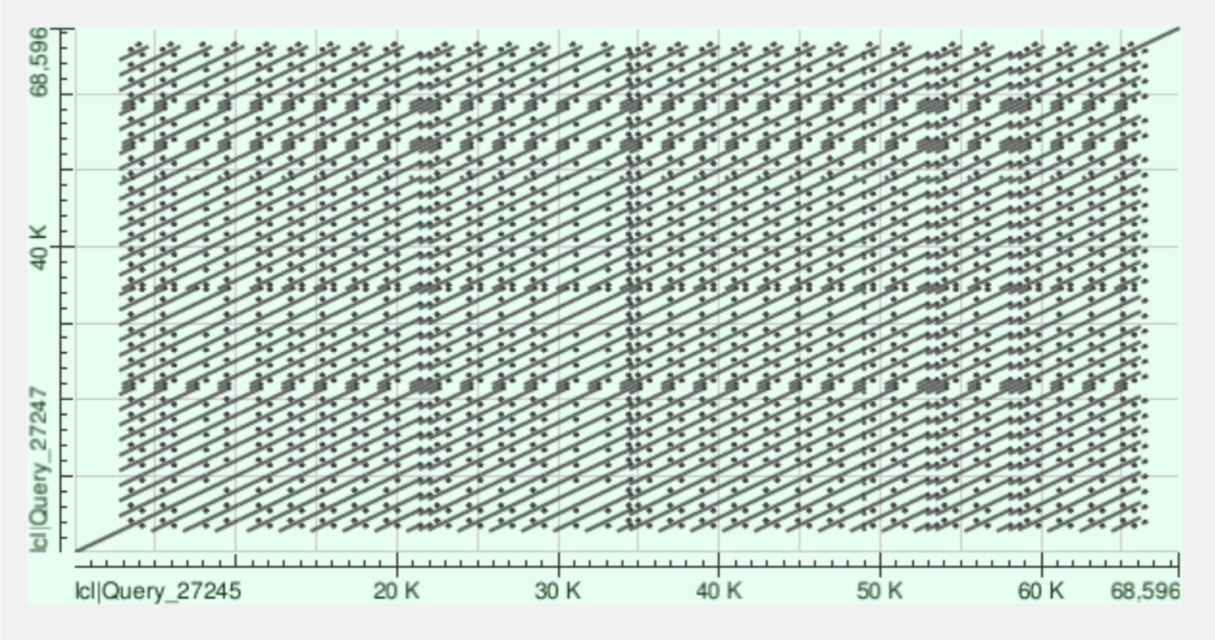

A 2,000 bp repeat is present 30 times in a 63,500 bp region between TRGV1-8 and TRGV1-9P.

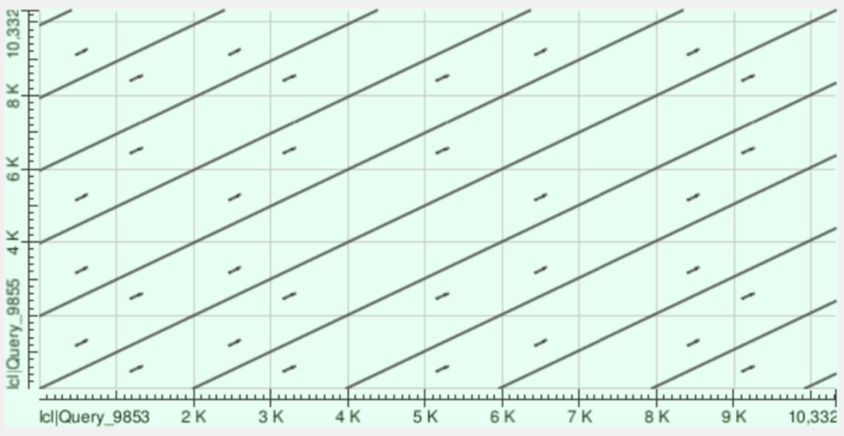

The region between 152,469-162,800.

Clonotype analyses of RNA-seq data from three individual catfish.

Fish	Total # of V-(D)-J sequences	Resolved	% Resolved	Clonotypes	# of TRAV subgroups	TRAJ segments	# of TRBV subgroups	TRBJ segments	# of TRGV subgroups	TRGJ segments	# of TRDV subgroups	TRAV/TRDJ Usage	TRDV/TRAJ Usage
SRR392744	241	222	92.12	212	**98**		**80**		**17**		**27**		
					6	54	17	26	3	5	2	5	2
2_HK	147	143	97.28	122	**61**		**59**		**12**		**11**		
					3	38	16	21	2	4	1	7	0
42_HK	97	93	95.88	81	**38**		**32**		**11**		**12**		
					4	26	15	18	1	3	1	2	0
# in the genome					6	125	30	31	3	10	2		

The total number of V-(D)-J sequences identified in each dataset are listed to the left. Resolved, indicates the number of sequences for which specific TRV subgroups and TRJ genes could be assigned using IMSEQ. Clonotypes, refers to the number of unique CDR3 sequences. In bold are the number of resolved sequences for each TR subtype. Below this are the numbers of TRV subgroups and TRJ genes for each TR subtype represented in the resolved dataset. The number of TRAV sequences rearranged to TRDJ, as well as the number of TRDV sequences rearranged to TRAJ are indicated.

Deduced amino acid sequence of catfish TRAV germline genes and cDNAs

```
TRAV1-6   MFTVIFVCLWLLLGDSMANSIEALFIHKVVDEDDNVTLSCSYKFSVLGTNYLHWYRQYPKSTPEFLLYISQSGDLSFNIPPRMSVEVNGDKVHLLISFAAVSDSALYYCALAP
AEM44666                DSMADSIEALFIHKVVDEDDNVTLSCSYKFSVSGTNYLHWYRQYPKSTPEFLLYISQSGDLSFNIPPRMSVEVNGDKVDLLISFAAVSDSALYYCALAP

TRAV1-13  MFTVIFICLWLSLGDSMADSIAPIFTQKVVDEGGDVTLSCRYKTNSSVANNYLHWYRQYPKSIPKFLLYIHQRGTLSANTPPRMSAEVHDDNNNKKQVDLLISSAAVSDSALYYCALQP
TS32.5a              SIGDSMADSIAPIFTQKVVDEGGDVTLSCRYKTNSSVANNYLHWYRQYPKSIPKFLLYIHQRGTLSANTPPRMSAEVHDDNNNKKQVDLLISSAAVSDSALYYCALQP

TRAV1-20   MFTVIFMCLWLSLGDSMANSIEPLVTHKVADEGDNVTLSCNYSGSINNLQWYRKYPKYNPEFLLYIFESGDLSHNIPPQMSAKVHKDKQVDLLISSAAVSDSALYYCALVP
TS32.17a              SIGDSMANSIEPLVTHKVADEGDNVTLSCNYSGSINNLQWYRKYPKYNPEFLLYIFESGDLSHNIPPQMSAKVHKDKQVDLLISSAAVSDSALYYCALVP

TRAV1-39  MFTVIFTCMWFSLGDSMADPIRPLVSHNVVDEGDNVTLSCNYKGFTGSIYNVQWYRQYPKSKPDFLLYITPSGELSLNIPPRMSANINGEKQVDLLIFSTAVSDSALYYCALVP
TS32.43a          MWFSLGDSMANPIEPLVSHNVVDEGDNVTLSCNYKGFTGSIYNVQWYRQYPKSKPDFLLYITPSGELSLNIPPRMSANINGEKQVDLLIFSTAVSDSALYYCALVP

TRAV1-47   MFTVIFTCLWLSLGDSMADSVGPRFTHKVVDEDDDVTLSCSYKTTSPTGNYLHWYRQYPKSTPEFLLYISDGGALSSNIPTRMTAKVNRDNKEVDLLISSAVVSDSALYYCALVP
TS32.34a              SIGDSMADSVGPRFTHKVVDEDDDVHLSCSYKTTSPTGNYLHWYRQYPKSTPEFLLYISDGGALSSNIPTRMTAKVNRDNKEVDLLISSAVVSDSALYYCALVP

TRAV1-50  MLSVIFMCIWLSIGDSLADPIEPLLTHKVVDEGHDVTLSCSYKDFSGTVRTLQWYKQYPKSTPEFLLYITPGGVKSDSVPRLSAKVDDKKQVDLLISSAAVSDSALYYCALEP
Ta4       MLSVIFMCIWLSIGDSLADPIEPLLTHKVVDEGHDVTLSCSYKDFSGTVRTLQWYKQYPKSTPEFLLYITPGGVKSDSVPRLSAKVDDKKQVDLLISSAAVSDSALYYCAL

TRAV1-77  MFLVILVCLWLSLSDSMTEAIKPLFSHKVVHEDDDVTLSCSYKDFSGSVNNLQWYRQYPKSKPEFLLYITPSGVKSPSIPPRLSAEVDDDNNKKQVDLLISSAAVSDSALYYCALVP
TS32.15   MFLVILVCLLGSLSDSMTEAIKPLFSHKVVHEDDDVTLSCSYKDFSGSVNNLQWYRQYPKSKPEFLLYITPSGVKSPSIPPRLSAEVDDDNNKKQVDLLISSAAVSDSALYYCALRP

TRAV3-1   MVRLFLFFFTIADIAEAADNSITPDQTSMSVIEGSNITLSCTYTGSVYSLHWYRQQPGSRPEFLLLIDETSEHVTQAQPPHPQLSTKLDKKNTKVDLLISSVTVTDSALYYCALMP
Ta1                                  SMSVIEGSNITLSCTYTGSVYSLHWYRQQPGARPEFLLLIDEASEHVTQAQPPHPQLSTKLDKKNTKVDLLISSVTVTDSALYYCAL

TRAV4-2   MLLFFIFIVVKNIAAVDSSITPDQTIISSSEGSITTLTCTYNQSAYSLHWYRQKPQSGPEFLLLIIVSSNDVINAKQPDPRLSIRLREGKKVDLEIFPAAVSDSALYYCALEP
Ta28      MLLFFIFIVVKNIATVDSSITPDQAIISSSEGSITTLTCTYNQSADSLHWYRQKPQSGPDFLLLIIVSSNYVIEAKQPDPRLSIRLRKDNKVDLEIFPAAVSDSALYYCAL

TRAV4-5   MLLFFVFIVIKNIAGAVDNSITPDQTIISSSEGSNTTLSCTYDASAYRLHWYRQKPQSGPEFLLMIRVSSDAVTEAKQPDPRLSTKLHKNGNKVDLEIVTVAVSDSALYYCALE
ET56.2a   MLLFFVFIVIKNIAGAVDNSITPDQTIISSSEGSNTTLSCTYDASAYRLHWYRQKPQSGPEFLLMIRVSSDAVTEAKQPDPRLSTKLHKNGNKVDLEIVTVAVSDSALYYCAL

TRAV4-6   MLLFFVFIVIKNIAGAVDNSITPDQTIISSSEGSNTTLSCTYDASAYRLHWYRQKPQSGPEFLLMIRVSSDAVTEAKQPDPRLSTKLHKNGNKVDLEIVTVAISDSALYYCALEP
ET56.13a  MLLFFVFIVIKNIAGAVDNSITPDQTIISSSEGSNTTLSCTYDASAYRLHWYRQKPQSGPEFLLMIRVSSDAVTEAKQPDPRLSTKLHKNGNKVDLEIVTVAISDSALYYCAL

TRAV6-1   MMVFSTVLFFTMFMGESTQDSITPTSSAVYAKEEQAVTLSCIYEYTVSMNNLQWYRQYSNAAPDFLVLLTESGANQTGDTPHPHLSAKVHKDLKRVDLEISFSALSDSALYYCALQP
ET35.1a   MMVFSTVLFFTMFMGESTQDSITPTSSAVYAKEEQAVTLSCIYEYTVSMNNLQWYRQYSNAAPDFLVLLTESGANQTGDTPHPHLSAKVHKDLKRVDLEISFSALSDSALYYCALQ
```

Accession numbers: TS32.5a_AAD56894, AEM44666, TS32.17a_AAD56896, TS32.43a_AAD56897, TS32.34a_AAD56895, Ta4_AAB94915, TS32.15_AAD56889, Ta1_AAB94917, Ta28_AAB02652, ET56.2a_QIP58417, ET56.13a_QIP58421, ET35.1a_MN313366

Deduced amino acid sequence of catfish TRBV germline genes and cDNAs

```
TRBV2-1    MIRILVFFQSLYWIQGVAGVNDVSQPNILWAKFGQSATINCSHTKGSSYNRMYWFRQHHGESMELIVYTGTFITADFGKFSQSKFSTIKTVAESGSFTVNDVDYNDSAVYFCAVSE
ET35.1b    MIRILVFFQSLYWIQGVAGVNDVSQPNILWAKFGQSATINCSHTKGSSYNRMYWFRQHHGESMELIVYTGTFITADFGKFSQSKFSTIKTVAESGSFTVNDVDYNDSAVYFCAVSE

TRBV2-6    MIRIIIIFQSLYWIQGVAGANDVLQPEILWAQFGQSVTINCSHTKGSAYREMYWFRQYQGESMELIVYTTSFGTPDFGKSDQKKFSAIKTVPENGSFTVKDVDYNDNAVYFCAVRE
Tb18       MIRIIIIFQSLYWIQGVAGANDVLQPDILWAQFGQSVTINCSHTKGSVYREMYWFRQYQGESMELIVYTTSFGTQDFGKSDQKKFSAIKTVPENGSFTVKDVDYNDNAVYFCA

TRBV3-2    MCTVLIIVSATLLCLAGSAVFTVDQNPPDLIKYQNETVEMKCEHSVNTYDRMLWYKHSQDTGFKYMGYLNTIFPKEEAEFGTKIKLSGDGRKSGSMTINSLSVNDSAVYFCVAY
Tb8            LIIVSATLLCLAGSRVFTVDQNPPDLIKYQNETVEMKCEHSVNTYDRMLWYKHSQDTGFKYMGYLNTIFPKEEAEFGTKIKLSGDGRKSGSMTINSLSVNDSAVYFCVA

TRBV7-5    MTRFPFNLIICVSCLSGSFFCKDVHQSPADVLCLAEESVTLTCNQSMLTYNTILWYQRTHGDTGLKLIGYVYYTITKQVEKDYEGNFTVDGDGESSASLHIPKARQVLHSALYFCAASY
ET56.13b   MTRFPFNLIICVSCLSGSFFCKDVHQSPADVLCLAEESVTLTCNQSMLTYNTILWYQRTHGDTGLKLIGYVYYTITKQVEKDYEGNFTVDGDGESSASLHIPKARQVLHSALYFCAAS

TRBV7-3    MIRFSYKFIISVCFLSGSLICKDVHQSPADVLCLPEESVTLTCNHSIPSYYTILWYQRTQDDTGLKLIAYISNTSPQYEGNYEGNFTVSGNGRSSAFLQIPKARQGLHSALYFCAAYY
FD055735   MIRFSYKFIISVCFLSGSLICKDVHQSPADVLCLPEESVTLTCNHSIPSYYTILWYQRTQDDTGLKLIAYISNTSPQYEGNYEGNFTVSGNGRSSAFLQIPKARQGLHSALYFCAAYY

TRBV7-15   MITFPFKLIICVTCLTGSSICKDVHQNPTDVLCLPEESVTLTCKHSIPNYNTILWYQRTHGDTHLKLIAYVFYTTPKYEGNYEGNFTVSGDGQSSASLQISKATQVLHSALYFCAAFY
TS32.34b   MITFPFRLIICVTCLTGSSICKDVHQNPTDVLCLPEESVTLTCKHSIPNYITILWYQRTHGDTHLKLIAYVFYTTPKYEGNYEGNFTVSGDGQSSASLQISKATQVLHSALYFCAAFF

TRBV11-4   MISVIISLTFLLYWKSVLAGENGVTQNPSVAWHLKGESAEMKCSHNKGSGYYQMYWYRQRQGESMEFIVYTTTSESDFGSVDKNKFSTVKKNAANGSLTVKDLDTEDSAVYFCAVKE
FD015772   MISVIISLTFLLYWKSVLAGENGVTQNPSVAWHLKGESAEMKCSHNKDAGYYQMYWYRQRQGESMEFIVYTTTSESDFGSVDKNKFSTVKKIAANGSLTVKDLDTEDSAVYFCAV

TRBV12-3   MSIVLLIVTALFISDIGRSSAAKDVHQTPPDLIKNIQESTDLSCSHSIPNHEFMLWYKRSENKQLQLLGYLNSKFPYPEDSLKAKIELHGDGNNEGKLTIKNLQPDDSAVYFCAVRL
TS32.43b   MSIVLLTVTALFISDIGRSSAAKDVHQTPPDLIKNIQESTDLSCSHSIPNHEFMLWYKRSENKQLQLLGYLNSKFPYPEDSLKAKIELYGDGNNEGKLTIKNLQPDDSAVYFCAVG

TRBV14-6   MIRAASILWVLMICHRGFSQSDLVFQTPPDLFGNHKQSVKIQCEHSVPSYNQINWYRETQDQGLTLIGYQYRTSSPQIENDFKLKVEIAGDGSKNVSLTIKNLSSNDSVVHFCAAS
TS32.15b   MIRAASILWVLMICHRGLSQSDLVFQTPPDLFGNHKQSVKIQCVHSVPSYNQINWYRETQDQGLTLIGYQYRTSSPQIENDFKLKVEIAGDGNKNVSLTIKNLSSNDSVVSFCAA

TRBV15-2   MITALLILALHHFSGQVDGSGVFQMPDIIWGSLGNSAEMNCSHNKDITYRQMYWFKQLPGEGITLLVFTSVGGEPDYGKFSKDKYEAIKTVAESGSLTVKTLDQGDDALYFCAVSQ
ET56.2b    MITALLILALHHFSGQVDGSGVFQMPDIIWGSLGNSAEMNCSHNKDITYRQMYWFKQLPGEGITLLVFTSVGGEPDYGKFSKDKYEAIKTVAESGSLTVKTLDQGDDALYFCAV

TRBV19-2   MAKIIVSSIILISFKAAVISSLNIQQTPQRLLMTTEQTEAKLKCHHGDASYPYMYWYQQKTVSDSIELIGMLQYGTSTPEEKFKARFNISGHATGDAFLLISSITTEDSAVYFCAASK
TS32.17b   MAKIIVSSIILISFKAAVISSLNIQQTPQRLLMTTEQTEAKLKCHHGDASYPYMYWYQQKTVSDSIELIGMLQYGTSTPEEKFKARFNISGHATGDAFLLISSITTEDSAVYFCAAS

TRBV23-2   MTGTLLYILLFILMKGSCMGVLITQWPKYISSFKSTSVDMHCYQNDTDYDYTYWYRQIEGKEPVLIARYVAGSPIQEKGFENGFKAWGTKKKWSLTVDVEEDSDAVYLCAASF
TS32.1b         LILLLFILMKGSCMGVLITQWPKYISGFKSTSVDMHCYQNDTDYDYTYWYRQIEGKEPVLIARYVAGSPIQEKGFENGFKAWGTKKKWSLTVDVEEDSDAVYLCAARD

TRBV24-1   MYANVSKLCLFLYLFTGRTNCANIQQSSSLLVKETQNVTIQCSHENNNLYVMLWYQQKTNGGMALIGYSYGMTEPKNEEDFKDRFEQSRQSIMAGKLTISKVLQSDSAVYYCAARE
Tb5        MYANVSKLCLFLYLFTGRTNCANIQQSSSLLVKETQNVTIQCSHENNNLYVMLWYQQKTNGGMALIGYSYGMTEPKNEEDFKDRFEQSRQSIMAAKLTISKVLQSDSAVYYC

TRBV27-1   MYGTCGLCVFFILFFERTNCVKFLQTPSLIENESSNVTIQCSHDDSSLPRMLWYQQNSRTVMALIGYTAGASSDPNYEDGFKDRFKQSRQGTLNGSLTISNLRQSDSAVYYCAASM
Tb4                       ERTNCVKFLQTPSLIKNESSNVTIQCSHDDSGLPRMLWYQQNSRTVMALIGYTAGASSDPSYEDGFKDRFKQSRQGTLNGSLTISNLRQSDSAVYYCAA

TRBV27-3   MYGACGLCVVFILLFERTNCVKFEQTPSILANETSEITIQCSHDDSSLYTMLWYQQNSRTVMALIGYTAGASSDPNYEDGFKDRFKQSRQGTLKGNLTISSLRQSDSAVYYCAASM
TS32.12b                  ERTNCVKFEQTPSILATETSEITIQCSHDDSSLYTMLWYQQNSHTVMALIGYTAGASGGPNYEDGFKDRFKQSRQGTLKGNLTISSLRQSDSAVYYCAAS
```

```
TRBV27-8  MDGSCELIVFFILCFGRINCVKFQTISSLLVNETEEVTIQCSHNDNTLQTMLWYLQNSNTVMALIGYTYTATSKPEYEDGFNVRYKQSRKSITEGSLTISKLLQSDSAVYYCAARM
Tb2         CSCELIVFFILCFGRINCVKFQTISSLLVNETEEVTIQCSHNDNTLQTMLWYLQNSNTVMALIGYTYTATSKPEYEDGFNDRYKQSRKSITEGSLTISKLLQSDSAVYYCAA

TRBV27-9  MNCTCGLCVVFILFFGRTNCAKVQQTPSILVNEKENITVSCSHNDNNLDRMLWYRQNSRTVLALIGYTMTAKSDPKYEEEFNDRFTLSRQGTLAGTLTISNLRQSDSAVYYCAASQ
TS32.5b               LFFGRTNCAKVQQTPSILVNEKENITVSCSHNDNNLDRMLWYRQNSRTVLALIGYTMTAKSDPKYEEEFNDRFTLSRQSTLAGTLTISNLRQSDSAVYYCAAS

TRBV27-15 MMYGTCGLCVFFVLFLAGKANCVKVEQTPLLLANETSDVTIHCRHDDGTLPVMLWYQQNSKTVMSLIGYTSGASGDPNYEDGFKDRFNLGRKSTTEGSLTISNLGQSDSAVYYCAARK
FD370658                                      TSDVTIHCRHDDGTLLVMLWYQQNSRTVMSLIGYTSGASGDPNYEDGFKDRFNLGRKSTTEGSLTISNLGQSDSAVYYCA

TRBV30-1  MACLHVILYFVTIDWLFTNCQQLIQVQQEPDDLVLSPGSSVKVSCAITGTNNPDLFWYRWNEAAGFVLVFSSRGAGMMDPVSEGQFKSNRPTDLQMVLESEGLSEIGSAVWYCAAS
TS32.49b       VILYFVTIDWLFTNCQQLIQVQQEPDDLVLTPGSSVKVSCAITGTNNPDLFWYRWNEAAGFVLVFSSRGAGMMDPVSEGQFKSSRPTDLQMVLESEGLSEIGSAVWYCAAK

TRBV30-7  MMHLHLSLFILTVHWFSNICMQLIQVQQDTRDLVLSQGLSLKVSCSITGLSIPTLYWYHWNKTAGFTLVFTSFATGSVNPASDGQFKSHRPDWLQIILESDAVSEIGSAVWYCAASS
FD028470  MIHLHLSLFILTVHWFSNICMQLIQVQQDTRDLVLSQGLSLKVSCSITGLSIPTLYWYHWNKTAGFTLVFTSFATGSVNPASDGQFKSHRPDWLQIILESDAVSEIGSAVWYCAAS
```

Accession numbers: ET35.1b_QIP58416, Tb18_AAA99773, Tb8_ AAB94920, ET56.13b_QIP58422, FD055735, TS32.34b_AAD56904, FD015772, TS32.43b_AAD56906, TS32.15b_AAD56898, ET56.2b_MN313369, TS32.17b_AAD56905, TS32.1b_AAD56902, Tb5_AAB94919, Tb4_AF038162, TS32.12b_AAD56900, Tb2_AF038159, TS32.5b_AAD56903, FD370658, TS32.49b_AAD56899, FD028470

Figure S1: The 115 TRBV genes in catfish form 29 subgroups.
Deduced amino acid sequences of catfish TRBV genes are listed according to subgroups
The two largest subgroups are TRBV27 with 16 members, and TRBV7 with 15 members. The smallest subgroups contain from 1-3 genes, and there are 14 single member TRBV subgroups. The TRB subgroups are presented in numerical order in the amino acid alignment. TRBV pseudogenes are in red and labeled P, stop codons are marked as an asterisk symbol (*), and minimal gaps were introduced to maximize the alignments. Previous family names are also provided.

Figure S2. Catfish and zebrafish TRBJ subgroups do not demonstrate strong phylogenetic relationships.
(A) The evolutionary history was inferred using the Neighbor-Joining method (Saitou and Nei, 1987). The percentage of replicate trees in which the associated taxa clustered together in the bootstrap test (10,000 replicates) are shown next to the branches (Felsenstein, 1985). The tree is drawn to scale, with branch lengths in the same units as those of the evolutionary distances used to infer the phylogenetic tree. The evolutionary distances were computed using the p-distance method (Nei and Kumar, 2000) and are in the units of the number of base differences per site. This analysis involved 62 nucleotide sequences. All ambiguous positions were removed for each sequence pair (pairwise deletion option). There were a total of 57 positions in the final dataset. Evolutionary analyses were conducted in MEGA X (Kumar et al., 2018). Species are indicated by color, *Danio rerio* (red) and *Ictalurus punctatus* (blue) **(B)** Nucleotide alignments of catfish and zebrafish TRBJ sequences sharing strongest phylogenetic topology. Nucleotide alignments display sequences for catfish J2-1 and J2-2P with zebrafish J2-1; catfish J1-28 with zebrafish J1-8; and catfish J29 and J24 with zebrafish J30, respectively.

Figure S3: The catfish TRAV and TRDV genes are dominated by 2 subgroups.
Deduced amino acid sequences of catfish TRAV and TRDV genes are listed according to subgroups. The catfish TRAV genes form TRAV six subgroups. The largest subgroups are TRAV1 (79 members), and TRAV2 (13 members). The smallest TRAV subgroups (3 and 5) consist of 3 members, and the TRA subgroups are presented in numerical order in the amino acid alignment. The catfish TRADV genes for two subgroups: subgroup 1 consist of 31 TRDV genes, nine of these are pseudogenes. Subgroup TRDV2 consists of only 4 genes. The TRAV and TRAD pseudogenes are in red and labeled P, stop codons are marked as an asterisk symbol (*), and minimal gaps were introduced to maximize the alignments. Previous family names are also provided.

Figure S4. Catfish TRAV and TRDV genes are dominated by 2 subgroups, and are less diverse than zebrafish TRAV and TRDV genes.
The evolutionary history was inferred using the Neighbor-Joining method (Saitou and Nei, 1987). The percentage of replicate trees in which the associated taxa clustered together in the bootstrap test (10,000 replicates) are shown next to the branches (Felsenstein, 1985). The tree is drawn to scale, with branch lengths in the same units as those of the evolutionary distances used to infer the phylogenetic tree. The evolutionary distances were computed using the p-distance method (Nei and Kumar, 2000) and are in the units of the number of base differences per site. This analysis involved 289 nucleotide sequences. Prior to analysis, sequences were trimmed to include 22 codons before the codon for the first cysteine (CYS23) to 6 nucleotides past the last cysteine

Figure S5. Dot plot analyses of the catfish TRAD and TRB loci.
The Dot plot analysis of the catfish TRAD locus encompasses nucleotides 446,400-1,446,399, and was created using the blastn suite 2-sequences (1) using the default parameter, except that the word length was set at 128. Tandem duplications are visible as diagonal lines. The red boxes show TR regions with duplication events

Figure S6. **Deduced amino acids of catfish TRAJ genes.**
The 125 catfish TRAJ genes are placed into groups based on members sharing 75% nucleotide identity, and 20 of them do not encode the canonical FGXG motif. Ten FGXG variations were observe and are highlighted in yellow.

Figure S7. Deduced amino acid sequences of catfish TRGV genes and TRGJ genes.
The largest TRG subgroup is TRGV2 which consists of eight functional TRVG genes and two pseudogenes. TRG subgroup 3 consists of four TRGV genes and TRGV1 forms its own subgroup. The TRGV pseudogenes are in red and labeled P. The red slash in TRGV2P indicates that splicing of the leader exon to the TRGV2P results in an unproductive transcript. Minimal gaps were introduced to maximize the alignments, and the previous subgroup names are provided. The catfish TRGJ genes are listed below, and as above TRGJ pseudogenes are in red. The 3' J gene segment splice site is marked with the / symbol.

Figure S8. Dot plot analysis of the repeat in the TRG locus.
The dot plot analysis of the 68,596 bp sequence from TRGV2-8 to TRGV2-9P was created using the blastn suite 2-sequences using the default parameters. The TRG locus is interrupted by a 2kb sequence that is repeated 30 times between TRGV2-8 and the TRGV2-9P.

Figure S9. Clonotype analyses of RNA-seq data from three individual catfish.
The total number of V-(D)-J sequences identified in each dataset are listed in the left. Resolved, indicates the number of sequences for which specific TRV subgroups and TRJ genes could be assigned using IMSEQ. Clonotypes, refers to the number of unique CDR3 sequences. In bold are the number of resolved sequences for each TR subtype. Below this are the numbers of TRV subgroups and TRJ genes for each TR subtype represented in the resolved dataset. The number of TRAV sequences rearranged to TRDJ as well as the number of TRDV sequences rearranged to TRAJ are indicated.

Figure S10. Alignment of catfish TRAV and TRBV germline genes to cDNA sequences. The alignment of the deduced amino acids demonstrates that TRA and TRB cDNA sequences from our laboratory match to catfish TRAV and TRBV genes present in the germline. Any amino acid differences are shown in red, and the cDNA accession numbers are also provided.
